# AI-Ready Multimodal Wearable Biosensors Beyond Glucose: Biofluid Sampling, Sensor Fusion, and Clinical Translation

**DOI:** 10.3390/bios16080448

**Published:** 2026-08-18

**Authors:** Ahmet Akif Kızılkurtlu, Ali Akpek

**Affiliations:** 1 Department of Biomedical Engineering, Faculty of Engineering and Natural Sciences, Istanbul Atlas University, 34403 Istanbul, Türkiye; 2 Department of Biomedical Engineering, Yıldız Technical University, 34220 Istanbul, Türkiye

**Keywords:** wearable biosensors, flexible bioelectronics, continuous biochemical monitoring, sweat sensing, microneedles, microfluidics, artificial intelligence, clinical translation

## Abstract

Continuous glucose monitoring has established that a molecular signal can be repeatedly measured in daily life and translated into clinically meaningful action. The next frontier is broader: wearable biosensors that monitor metabolites, electrolytes, hormones, drugs, nutrients, inflammatory markers, and tissue-state indicators beyond glucose. This review proposes an artificial intelligence (AI)-ready framework for multimodal wearable biochemical monitoring. We organize the field as a complete measurement chain that links biofluid access, sampling chronology, flexible biointerfaces, molecular recognition, signal conditioning, metadata capture, sensor fusion, clinical validation, and lifecycle governance. The central argument is that the clinically useful variable is rarely a raw current, potential, optical intensity, or spectrum; it is a quality-controlled, context-aware, and uncertainty-aware digital biomarker. We compare sweat, interstitial fluid, saliva, tears, wound exudate, and breath condensate; evaluate enzymatic, ion-selective, affinity, transistor, optical, and spectroscopic sensing strategies; synthesize representative high-impact studies; and define minimum metadata, validation metrics, and translation gates. The review highlights recurring gaps in biofluid validity, real-world robustness, reference-comparator alignment, subgroup evidence, and algorithmic governance. We conclude with practical design rules for converting flexible biochemical wearables from attractive prototypes into clinically credible intelligent biosensing systems.

## 1. Introduction: Why Beyond Glucose Now?

The development of wearable biochemical sensors has focused on a single application that serves as a paradigm in the field, i.e., subcutaneous continuous glucose monitoring (CGM) as a small-scale (and thus portable) and quasi-automated method for providing a molecular measurement of glucose in real time [1,2]. Advances in CGM have shown that it can provide real-time molecular measurements of glucose that can serve as an ongoing, consistent behavioral signal/therapy and support clinical workflow integration through “live” association with the subject via their daily behavior [3]. Nonetheless, it is important to note that the progression toward CGM devices does not constitute the whole of biochemistry for wearable sensors; this is also known as “the benchmark for translational success for biophysical wearable sensors”. In other words, “CGM has progressed through a clinical need for glucose, through development of interstitial fluid chemistry, through establishment of interstitial-fluid-CGM molecular measurement correlation, through an established treatment/therapeutic action (i.e., at what concentration of glucose should a specific treatment be administered), and through an established reimbursement ecosystem [4].” Most biomarkers outside glucose only possess some of these requisites to enable the establishment of QC systems, thus leading to the use of “beyond glucose” as a means to call for further development of capabilities for molecular monitoring via CGM devices, thus treating the body as a poly-analyte system [5,6]. Furthermore, disturbances (biochemical/intra-ionic/hemodynamic/behavioral/external) in physiological relationships result in significant derangement of body functions (examples include exercise intolerance and dehydration). Infection, medication response/side effects, endocrine function derangement, renal function impairment, wound healing failure, and dietary deficiency are multicollinear, and therefore, devices will not only require an accurate measurement of some physiological endpoint (e.g., glucose) to be used but also require an understanding of: where (i.e., what type of tissue or environment) in the body the physiological endpoint occurred; the means by which the physiological endpoint was assessed; whether the information obtained from assessing the physiological endpoint is reliable (i.e., is the information accurate); whether or not the body was undergoing additional physiological changes; and what physiologically based or scientifically based decisions the clinician will make as a result of using the information provided via the physiological endpoint. The trend in the scientific community has shifted from relying solely on individual flexible sensors to creating intelligent biochemical systems. New landmark platforms have been created to combine lactate sensors with electrocardiography, use droplet microfluidics to run repeated wet-chemical assays on fluids from tissues, integrate microneedles for monitoring metabolites in interstitial fluid, expand the analysis of sweat from measuring only nutrients and hormones to using chronological SERS (surface-enhanced Raman spectroscopy) for sweat fingerprint multiplexing, and develop neuromorphic or edge-processing architectures [7]. Recent studies have extended this systems-level transition from short exercise demonstrations to time-resolved and multiday sweat sampling, automated exhaled-breath-condensate analysis, early chronic-wound deployment, multiplex endocrine and pharmacokinetic sensing, and compact daily-wear biochemical formats [8,9,10,11,12,13,14]. These advances broaden molecular, anatomical, and temporal coverage. Still, they also reinforce that controlled biofluid access, matrix-specific reference comparisons, longitudinal drift assessment, and claim-specific human validation remain non-compensatory requirements. The limiting issue now is not only whether an analyte can be detected, but whether the full measurement device can provide interpretable and reproducible longitudinal patient data in a clinically actionable manner, and whether this can be conducted outside a lab.

For this review, a wearable biosensor is considered to perform a series of transformations. A biological state is first measured as a local concentration of an analyte in a biological fluid. Next, this concentration changes due to transport, secretion, dilution, evaporation, contamination, or extraction in the biological fluid. A recognition interface subsequently transforms the concentration into electrical, optical, or spectroscopic signals. The acquisition electronics, firmware, calibration, mechanical disruption, and environmental conditions also alter these signals. Ultimately, the transformed signal must be fused with other measured variables, such as temperature, pH, sweat rate, motion, skin impedance, heart rate, timing of medication administration, meal times, symptoms, and the individual’s baseline values. Each of these transformation stages can lead to the creation of new information, prevent the creation of information, and/or introduce bias into the information produced. Therefore, high-quality reviews of the literature in this field must focus on the entire series of transformations rather than on a specific sensor material(s) [15,16]. Accordingly, the term “AI-ready” is used intentionally in this review paper; this does not mean that the authors have used a trendy machine learning classifier in their manuscript; rather, it means that the measurement chain is capable of producing data that statistical or machine learning-based systems can reliably utilize. For the data produced by the measurement chain to be of sufficient quality to yield accurate measurements, it must include all components critical to the measurement process, including synchronized raw data streams, device metadata, calibration history, sampling-state labels, and indices of signal quality. Moreover, the measurement chain must also include information such as: reasons for missing data points; subject-level covariates; estimated measurement uncertainty; known conditions that alter device measurements (e.g., disqualified measurements); and diverse conditions that affect real-world measurements. If the above conditions are met, then use of AI technology will help to compensate for drift in the measurements, to align time constants between measurements, to help personalize each patient’s baseline measurements, to help identify disqualified measurements, and to convert trajectories from multiple channels of data measurement into “digital biomarkers” [17,18]. This review article accomplishes three goals. First, it provides a framework for organizing wearable biochemical measurement systems, from sampling physiological fluids to clinical translation. Second, the review includes practical tables and figures that researchers can use to design their studies, develop metadata schemas, and create validation plans to ensure AI readiness. Third, this review article aims to allow readers to delineate where: (1) analyte detection ends; (2) physiological feature extraction begins; (3) digital biomarkers begin; and (4) decision-support outputs end. This delineation is crucial for authors, as it can help them to avoid making overly extravagant claims while maintaining continuity in the development of flexible bioelectronic devices and intelligent biosensor systems.

## 2. Scope and Literature Selection Strategy

This review is more an analytical assessment grounded in the literature than a comprehensive evaluation of all the available literature. The primary purpose is to synthesize representative, high-impact research to derive design principles for multimodal biosensing devices that are compatible with artificial intelligence (AI) [19,20]. The literature reviewed was organized according to the entire data-generating process: access to biofluids, sampling methods, recognition chemistries, flexible substrate materials, electronic circuits, metadata associated with biological samples, algorithmic processes used to combine the information, and finally, the validation and/or clinical extension of the device. Studies were prioritized when they reported integrated wearable systems, human or on-body data, microfluidic or minimally invasive sampling, multianalyte capability, explicit clinical use cases, or advanced data-processing architectures. Search concepts included wearable biosensor, wearable chemical sensor, sweat sensor, interstitial fluid sensor, microneedle biosensor, microfluidic wearable, continuous biochemical monitoring, lactate wearable, cortisol wearable, aptamer wearable, molecularly imprinted polymer sensor, SERS sweat, neuromorphic wearable, AI biosensor, sensor fusion, digital biomarker, software as a medical device, and clinical validation. The emphasis was placed on reports from 2015 onward, with particular attention to recent articles, reviews, and related venues. Older work was included when it established a key concept, such as hybrid chemical–physical sensing or epidermal microfluidic collection. Because this manuscript focuses on biosensor systems, the selection also reflects the issue’s emphasis on flexible bioelectronics, intelligent sensing systems, wearable technology, AI- and machine learning-enabled biosensors, biomarker detection, and clinical applications [21,22]. Table 1 summarizes the framework-selection logic and extraction fields in this study.

**Figure 5:**
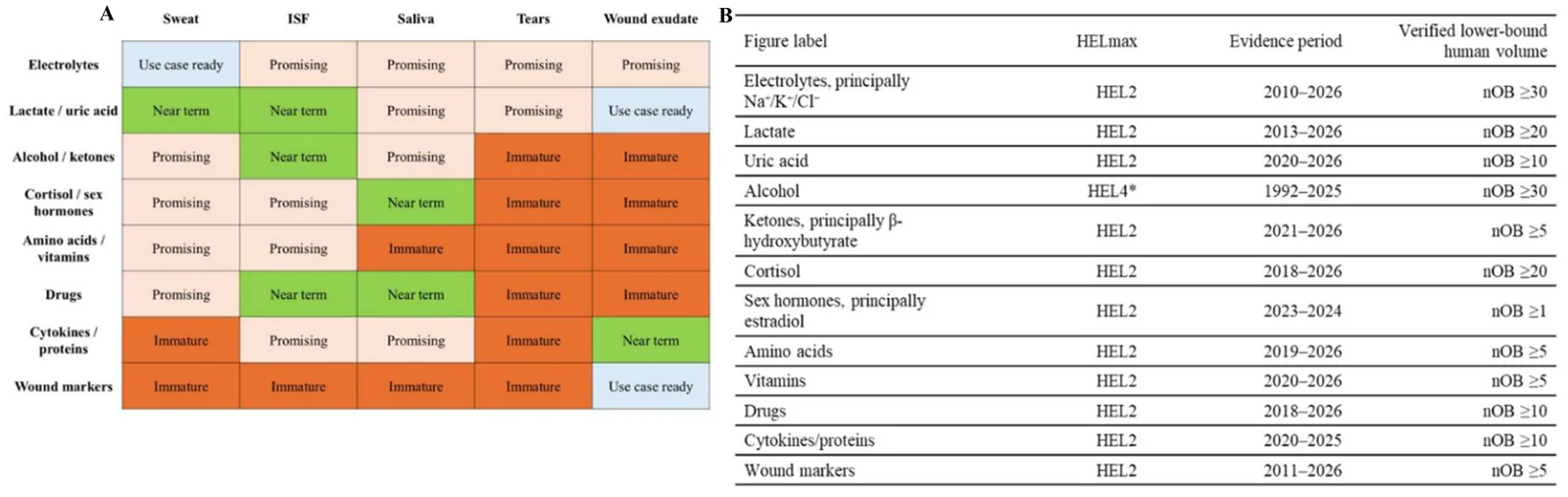
Biofluid-specific translational readiness and temporal human evidence map for beyond-glucose wearable biomarkers. (**A**) Author-developed comparative classification of biomarker families according to biofluid accessibility, sensing maturity, matrix and sampling control, and claim risk. The categories denote relative translational maturity and do not imply demonstrated clinical utility or regulatory authorization. (**B**) Temporal map of human evidence up to 31 July 2026. Each entry represents a defined analyte–biofluid–use-claim combination. Color denotes the highest Human Evidence Level achieved (HEL 0–4), symbol area represents the cumulative lower-bound class for the number of independent studies involving actual human wear or direct in situ clinical sensor contact (nOB), and horizontal lines extend from the first to the most recent qualifying study. Multiple reports derived from the same participant cohort were counted once. Paired families are shown separately when their evidence levels or study volumes differ. HEL describes the maturity of published human evidence only and does not represent complete device readiness, the AI-readiness criteria in Table 2, regulatory authorization. HEL4 for alcohol is restricted to sensor-contingent alcohol-reduction interventions and does not imply equivalence between transdermal and contemporaneous blood alcohol concentrations [5,6,18,25,26,27,96].

To add temporal and quantified human evidence dimensions to Figures 4 and 5, we conducted a targeted evidence audit up to 31 July 2026, supplementary to the narrative literature selection strategy used for the remainder of the review. PubMed searches were combined with backward and forward citation tracing of primary reports using biomarker-specific combinations of the terms wearable, on-body, in situ, sweat, interstitial fluid, saliva, tears, wound exudate, transdermal, microneedle, and human. Eligible evidence comprised peer-reviewed primary human studies in which the target analyte was measured during actual on-body or direct in situ clinical use, or a wearable-intended sensor was evaluated using the target biofluid obtained from identifiable human participants. Artificial-fluid-only, commercial or pooled human-matrix studies without an identifiable participant cohort, animal-only and in vitro studies, conventional sample-collection studies without wearable-intended sensor evaluation, reviews, editorials, abstracts, and preprint-only reports were excluded.

The unit used for evidence volume was the independent human study or participant cohort rather than the publication. Multiple reports derived from the same cohort were linked and counted once, whereas clearly non-overlapping cohorts were counted separately. A multiplexed study could contribute once to each relevant analyte but only once to an aggregate family total. Figure 5 uses nOB, the number of eligible studies involving actual human wear or direct in situ clinical sensor contact.

Human evidence was classified using an author-developed Human Evidence Level (HEL) scale informed by V3, IMDRF, and FDA fit-for-purpose principles. HEL 0 indicated no eligible human evidence. HEL 1 indicated human-matrix or on-body feasibility without a time-aligned, fit-for-purpose reference comparison. HEL 2 required quantitative on-body human measurement validation against a time-aligned orthogonal assay or appropriate reference method. HEL 3 required claim-specific clinical validity of a prospectively locked configuration or output in the intended or a closely matching population and context, supported by at least two independent participant cohorts or data sources. HEL 4 required prospective comparative or implementation evidence that use of the wearable output produced a prespecified patient, safety, behavioral, clinical-decision, or workflow benefit. HEL was assigned to the specific analyte–biofluid–use-claim combination and does not itself indicate complete device readiness, AI readiness, regulatory authorization, or passage through the stage-gate pathway [18,25,26,27].

## 3. Conceptual Framework: An AI-Ready Measurement Chain

**Figure 1:**
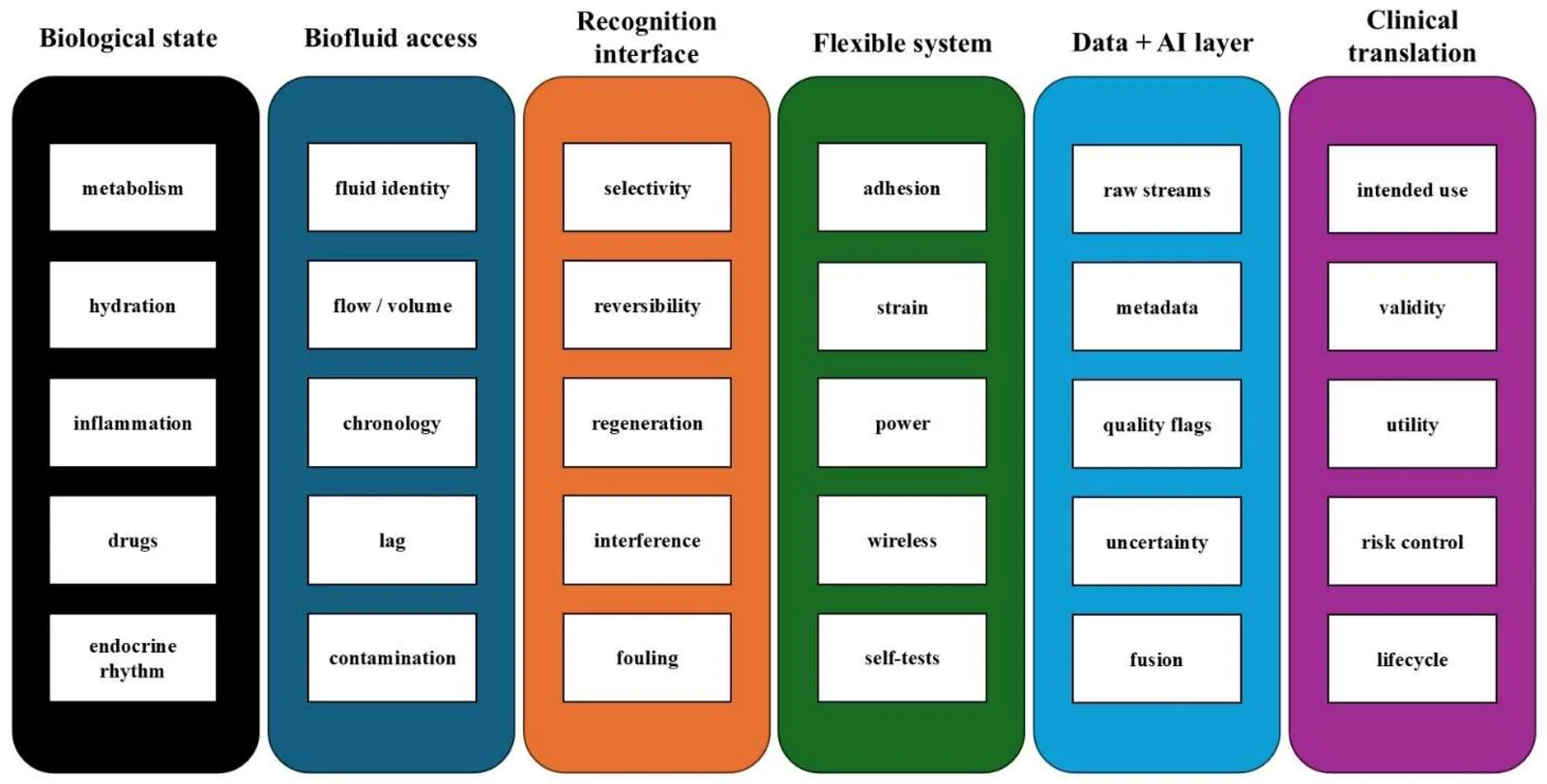
AI-ready multimodal wearable biochemical monitoring stack. The original schematic links biological state, biofluid access, recognition chemistry, flexible hardware, data governance, and clinical translation into one measurement chain. Abbreviation in figure: AI, artificial intelligence [15,21].

The primary design consideration for a multimodal wearable biosensor is not the electrode material, microcontroller, adhesive, or neural network, but rather how to define the measurement chain. An AI-ready measurement chain will originate from a clinical or biological question to produce a decision-relevant output. As such, the individuals involved in the design of the analyte panel, sample fluid, recognition mechanism, device mechanical design, metadata stream, algorithm(s), and validation study must provide input to the measurement chain to address the initial question. For instance, a lactate sensor developed for pacing purposes in athletes would not necessarily be appropriate for an individual to use to monitor for sepsis. Similarly, a cortisol sweat patch developed for stress research would not be appropriate for diagnosing an adrenal disorder in children. The same analyte target may therefore require different measurement inference methods, depending on the purpose [25,28]. The stacking diagram in Figure 1 illustrates how different aspects of the measurement chain interact. The left section of the stacking diagram indicates that access to a patient’s biofluid is considered an active participant in a measurement rather than a passive source of a sample. The center section of the stacking diagram shows how the recognition chemistry and flexible system design must be co-optimized. For example, ion-selective membranes require stable reference electrodes when measuring a target ion; enzymatic electrodes require the control of either oxygen concentration or a mediator before obtaining an accurate measurement; affinity sensors produce signals when the concentration of the target analyte or ligand is above a specific concentration and, therefore, must demonstrate a level of reversibility in their binding characteristics and have the ability to regenerate; and spectroscopic systems require reproducibility in terms of the sample being measured and apply consistent preprocessing procedures to the sample during the data collection process. Lastly, the right-hand section of the stacking diagram explains how the data and clinical layers interact to produce a decision-relevant output. For a device to be AI-ready, it should be able to capture signal quality and correction variables explicitly, define uncertainty in the measurement or device, and identify cycles in the device’s life cycle [29,30].

**Figure 2:**
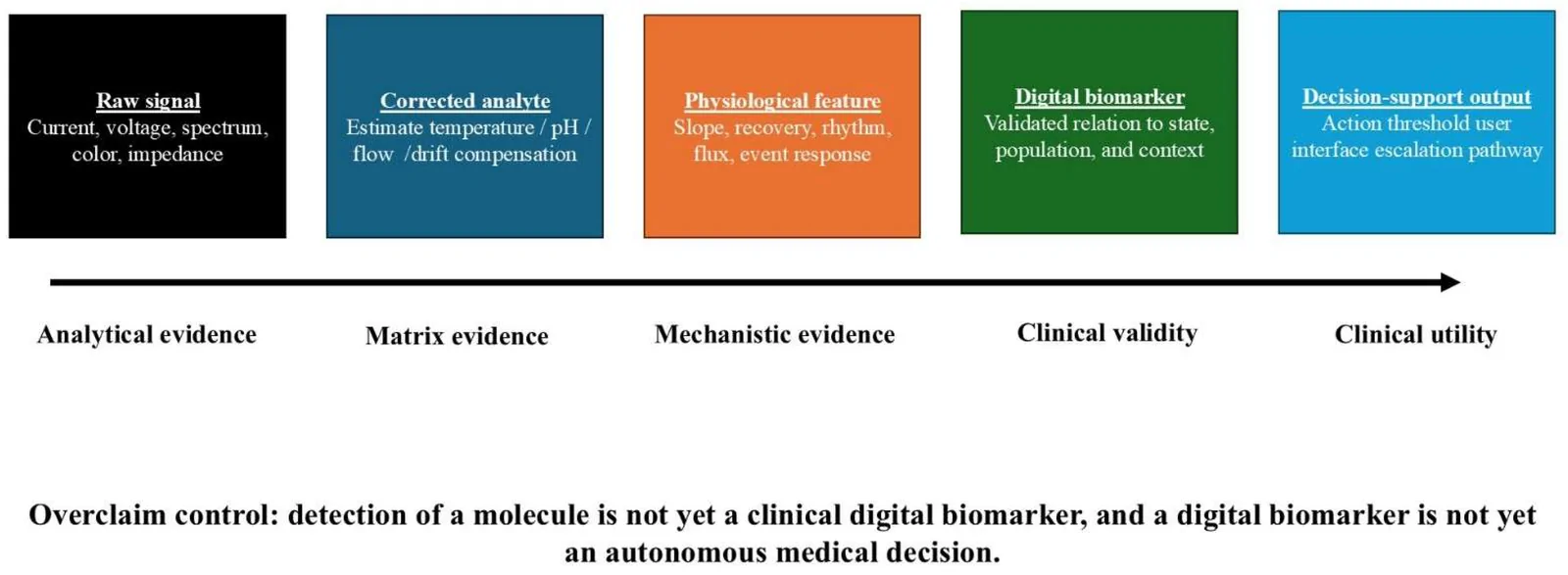
Claim hierarchy for biochemical wearables. Original schematic distinguishing raw signals, corrected analyte estimates, physiological features, digital biomarkers, and decision-support outputs [17,18].

Many studies focused on wearable biosensors contain poorly defined references to different levels of measurement. Raw current (also referred to as current trace) does not represent an estimate of analyte concentration, analyte concentration does not represent a physiological measurement, and physiological measurements do not represent a validated clinical digital biomarker [17,18]. Therefore, a digital biomarker does not represent a decision-support outcome. Figure 2 provides a procedure for applying the hierarchy of claims across the wearable biosensor field, but this is particularly important for biomarkers beyond glucose, for which specific measures of matrix concentration and action thresholds are typically less well established than for glucose. The purpose of the hierarchy is not to stifle innovation but to clearly define each area of research. For example, a collection of lactate data from sweat may represent an estimate of analyte concentration. A recovery slope from lactate data after a standard workout protocol may reflect a physiological response. Validated predictive capabilities from lactate levels, heart-rate variability, and sleep could, in combination, serve as a digital biomarker of delayed recovery. A recommendation to decrease training volume could be a decision-support outcome based on using the lactate value to reduce training load. Each claim has its own evidence base; however, publications often present the first and/or second claim as valid and then state unqualified incremental claims that the fourth and/or fifth claims are already valid. Such overclaiming has led to skepticism toward research in this area.

### What Makes a Wearable Biochemical Sensor AI-Ready?

AI readiness is evaluated here across four complementary and non-substitutable domains rather than as a single qualitative label: Technical, Contextual, Statistical, and Clinical Readiness. Technical Readiness concerns whether the sensor produces a stable and quantifiable analyte signal; Contextual Readiness determines whether that performance persists in authentic biofluid and representative on-body conditions with adequate temporal and contextual information; Statistical Readiness evaluates whether a locked processing and modeling pipeline generalizes across participants, devices, sites, and manufacturing lots; and Clinical Readiness determines whether the output agrees sufficiently with a fit-for-purpose comparator and supports the predefined intended use. Table 2 converts these domains into auditable operational pass criteria. A complete AI-readiness profile requires all four domains to pass, and failure of one claim-critical criterion cannot be compensated for by stronger performance in a later domain [18,24,28].

**Table 2:** The operational minimum criteria for the four AI-readiness domains of wearable biochemical sensing systems.

AI-Readiness Domain	Required Evaluation	Quantitative PASS Criteria	Automatic FAIL or Readiness Cap
Technical Readiness	Relevant-matrix testing at low, middle, and high quality-control concentrations for the full intended measurement duration. Raw and post-correction drift must be reported separately.	SNR_dec ≥10 at the lowest decision-relevant concentration, C_min. Raw normalized drift ≤5% of the decision-signal span per hour. After a prospectively locked correction, residual drift ≤2% of the decision span per hour and cumulative residual drift ≤10% of the decision span over T_wear.	SNR_dec <10 at C_min; raw drift >5% of the decision span per hour; failure to test the complete intended duration; or calibration/drift correction fitted after inspection of the test data. An SNR between 3 and <10 supports detection only and cannot support a quantitative AI-ready output.
Contextual Readiness	Authentic target biofluid and representative variations in flow, pH, temperature, motion, body site, contact, device age, and environmental conditions.	SNR_dec ≥10 in ≥90% of all prespecified QC-valid windows; residual drift ≤2% of the decision span per hour and ≤10% cumulatively in every claim-critical context stratum; ≥95% completeness of mandatory metadata; ≥90% usable-data yield over T_wear; and synchronization error no greater than one prespecified sampling interval. Failed devices and epochs remain in the denominator.	Selective exclusion of low-quality windows or failed devices; <95% critical-metadata completeness; <90% usable-data yield; unresolved time misalignment; or failure of the SNR or drift requirement in a claim-critical use condition.
Statistical Readiness	A frozen preprocessing, calibration, model, and operating threshold evaluated using participant-disjoint data from at least three independently manufactured lots/batches. Lot must not be confounded with participant, site, operator, day, or outcome.	The upper 95% confidence bound for residual drift is ≤1% of the decision span per hour, and cumulative residual drift is ≤5% across held-out devices/lots. For categorical outputs, LCB_95(F1_XB) ≥ F1_min^COU, UCB_95(D_lot) ≤0.05, and no lot has LCB_95(F1_b) < F1_min^COU −0.05. For continuous outputs, repeated-measures Bland–Altman point limits of agreement must lie within the prespecified allowable-difference band A(c) on the locked participant-independent test set.	Participant-, lot-, site-, or time-level leakage; model or threshold tuning on held-out data; fewer than three independent lots; failure of the cross-batch F1 or drift criteria; or treating repeated windows from the same participant as independent observations.
Clinical Readiness	Prospective and independent intended-use evaluation using production-equivalent lots, time-aligned comparator measurements, and the intended population, sites, workflow, wear duration, and decision regions.	SNR_dec ≥10 in ≥95% of intended-use windows at the clinical decision boundary. UCB_95(residual drift) ≤1% of the decision span per hour, with cumulative drift ≤min(5% of the decision span, 25% of the allowable analytical-error budget). For categorical outputs, the cross-batch F1 criteria and all safety-critical class-specific sensitivity and precision floors must be reproduced externally. For continuous outputs, LCB_95(LoA_L(c)) ≥−Δ_L(c) and UCB_95(LoA_U(c)) ≤+Δ_U(c) throughout the claim-critical range and prespecified subgroups. The prospective clinical or operational endpoint must also be met.	A post hoc clinical agreement band; correlation or R^2^ used as a substitute for agreement; only point limits of agreement reported; standard Bland–Altman analysis applied to repeated observations as if independent; a safety-critical subgroup or lot crossing its predefined floor; or failure of the intended-use endpoint.

In Table 2, the decision-level signal-to-noise ratio is defined as SNR_dec_ = |ȳ(C_2) − ȳ(C_1)|/σ_noise_, where C1 and C2 delimit the smallest decision-relevant concentration change, and σ_noise_ is estimated using a prespecified baseline or blank region. For signal types without a meaningful baseline-noise estimate, performance at C_min_ should instead be demonstrated by prospectively specified accuracy and precision criteria. Normalized drift is defined as D_rate = 100 × max_j_|β_j|/|ȳ(C_high_) − ȳ(C_low_)| and is reported as a percentage of the decision-signal span per hour, where β_j is the temporal slope measured at the low, middle, and high fixed-QC concentrations. Raw and residual post-correction drift are reported separately, and the correction is frozen before evaluation.

For B independent manufacturing lots and K output classes, F1_b^macro is the unweighted mean of the K class-specific F1 scores, and F1_XB_ = B^−1Σ^_bF1_ b^macro^ is the equally lot-weighted cross-batch macro-F1. Worst-lot degradation is D_lot_ = F1_XB_ − min_b(F1_b^macro). Confidence intervals are obtained by participant-level clustered resampling, retaining all repeated observations, failed episodes, and abstentions from each selected participant. F1_min_^COU^ is the minimum acceptable F1 score derived and locked from the context of use, class prevalence, comparator, action threshold, and consequences of false-positive and false-negative outputs.

For continuous measurements, the wearable–reference difference is d_i = W_i − R_i, bias is d^−^, and the 95% limits of agreement are LoA_L = d^−^ − 1.96s_d_ and LoA_U_ = d^−^ + 1.96s_d_. A(c) = [−ΔL(c), +ΔU(c)] is the maximum clinically allowable difference band specified before testing at concentration or decision region c. Repeated measurements are analyzed using a variance-component model or participant-level cluster bootstrap rather than being treated as independent pairs [24,28,31,32,33,34,35].

For equity-sensitive evaluation, prespecified strata and intersections should be selected from plausible measurement mechanisms, the intended population, and the consequences of error. The usable-data, performance, and agreement criteria in Table 2 are assessed both overall and within every adequately powered safety-critical subgroup. If a subgroup is too small for the prespecified confidence-bound decision, readiness remains unassigned rather than being inferred from a nonsignificant comparison. An aggregate PASS cannot compensate for a subgroup-specific FAIL or a materially higher rate of invalid input, missing data, quality-control rejection, or algorithmic abstention [24,28,29,36,37].

The SNR threshold of 10 was adapted from the ICH Q2(R2) convention for quantitative analytical performance and is used here as a lower-bound analytical anchor rather than as an established wearable-sensor standard. The normalized drift limits, coverage percentages, metadata requirements, and absolute cross-lot degradation margin of 0.05 are author-proposed operational defaults. A recognized analyte-specific standard or a prospectively justified context-of-use requirement takes precedence whenever it is more stringent. No universal absolute F1 score or fixed percentage Bland–Altman band is imposed because acceptable classification and agreement performance depend on the intended use, comparator uncertainty, prevalence, action threshold, and consequences of error. If F1_min_^COU^ or A(c) cannot be justified before testing, Statistical or Clinical Readiness cannot be assigned retrospectively [18,28,31,32,33,34,35,38].

Technical Readiness is assessed primarily at G1; Contextual Readiness at G2–G3; Statistical Readiness at G4; and Clinical Readiness at G5. G6 evaluates regulatory and lifecycle governance through documented controls rather than a single numerical performance metric. Compared with a benchtop assay, a wearable system must maintain these criteria at a continuously changing, aging, and heterogeneous biological interface [29,30].

The current approach has typically been to develop additional sensors to capture multimodal information. However, having multiple sensors alone does not equate to intelligence, since a biosensor with lactate, sodium, potassium, pH, temperature, accelerometry and heart-rate channels may still fail if the sensor data streams are not appropriately synchronized with one another, if pH compensation is not related to the analyte’s response, if motion artifacts are misidentified as metabolic transitions, or if the model was trained solely on healthy young volunteers with controlled trials. Therefore, it is necessary to plan sensor fusion before sensor construction and not to view reference electrodes, thermistors, strain gauges, impedance contacts, flow indicators, and microfluidic timing marks as accessories, but rather to consider them explanatory or descriptive variables of the sensor’s output data. A sensor designed for AI interpretation should include at least the minimum number of control signals necessary to differentiate between physiology and artifact [21,22]. This issue is critical not only for glucose sensors, because concentrations may be at or near their detection limits; secretion rates may vary considerably from one user to another; recognition layers could saturate; and blood/tissue state may have an indirect relationship to the output data.

## 4. Biofluid Sampling: The First Source of Signal and Bias

**Figure 3:**
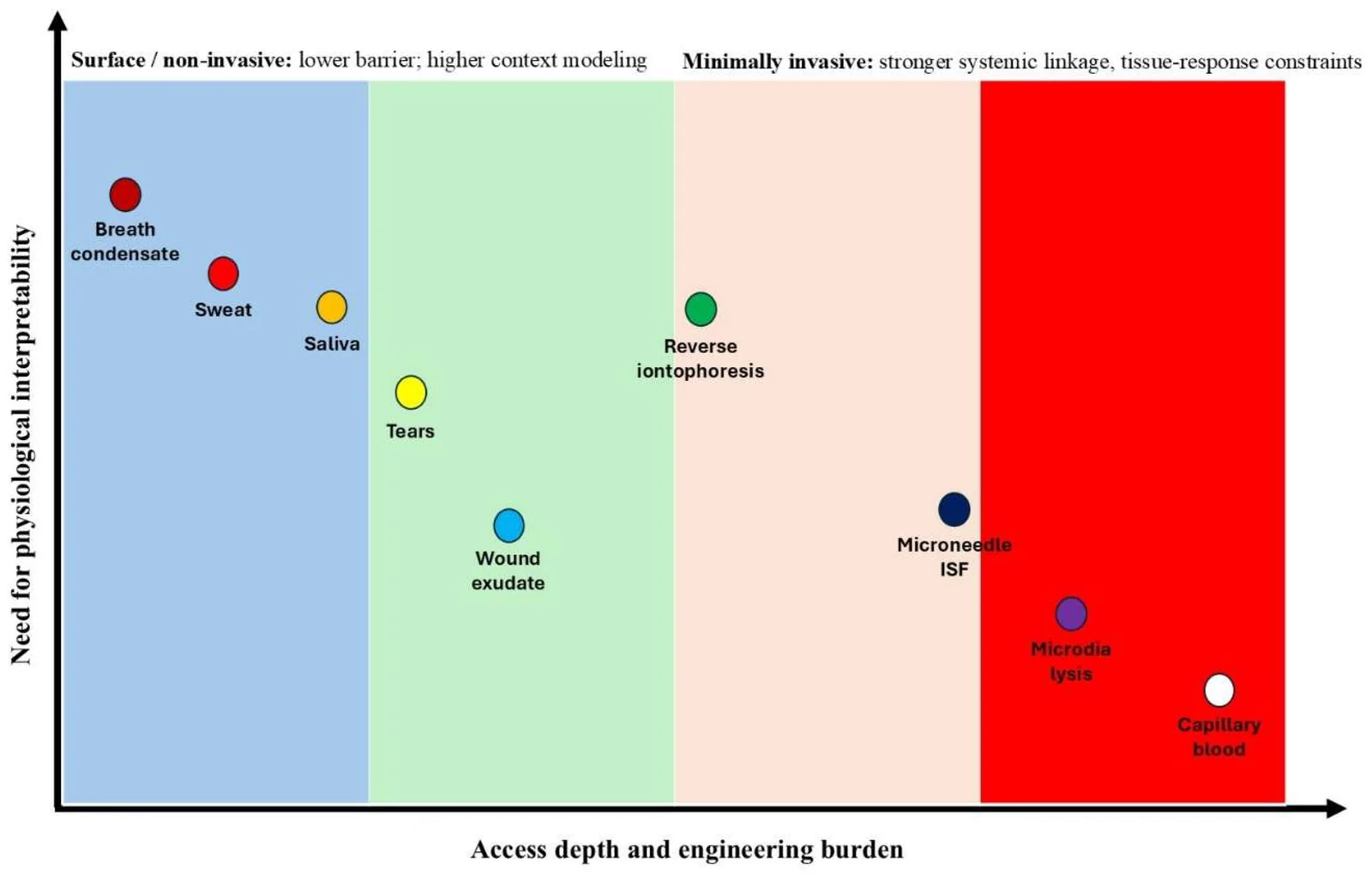
Biofluid access map for continuous biochemical monitoring. Original schematic positioning of major biofluids by access burden and physiological deconvolution requirements.

The selection of biofluids can also be determined from a biological perspective, and not all biofluids, such as sweat, interstitial fluid, saliva, tears, wound exudate, and breath condensate, can serve as direct substitutes for blood [15,39]. Each is derived from a different compartment, is transported via a different route, and exists in different states of local tissue and interaction with users. Although sweat monitoring is generally regarded as non-invasive and compatible with electronic skin interfaces, the degree of impurity in the sweat produced depends on many factors, including gland physiology, sweating rate, reabsorption, contamination, evaporation, and patch occlusion [40,41]. Interstitial fluid is similar to plasma with respect to many solutes and forms the basis for current commercial continuous glucose monitors; however, it requires barrier access and may exhibit a tissue response and a transport lag time [42,43]. Saliva and tears can be used for specific applications; however, they have non-systematic limitations involving hygiene, enzymatic activity, pH, ocular safety, and volume. Wound exudate does not circulate outside of the local tissue area; therefore, this may provide a benefit if the objective of the decision is to manage a wound. Breath condensate and volatile matrices/sampling may have utility in screening for respiratory and metabolic diseases; however, calibrating these continuous measurements would be challenging [44]. Figure 3 presents the primary biofluids on two conceptual axes—the depth of access and the extent of the need for physiological deconvolution—and Table 3 identifies the critical analytes contained in the primary biofluids for measuring the respective components. The intent of the figure is not to rank biofluids; rather, it is to demonstrate that each biofluid creates/removes previously discussed engineering and biological burdens. While surface matrices may provide convenience, the required context may necessitate additional model development. Although minimally invasive matrices may provide a closer approximation of a systematic characteristic, they require sterile, biocompatible materials and controlled mechanisms for tissue response. Blood is still the reference for most analytes, but it is generally only available intermittently, unlike the continuous daily monitoring of most parameters using blood.

### 4.1. Sampling Architectures and Chronology

**Figure 4:**
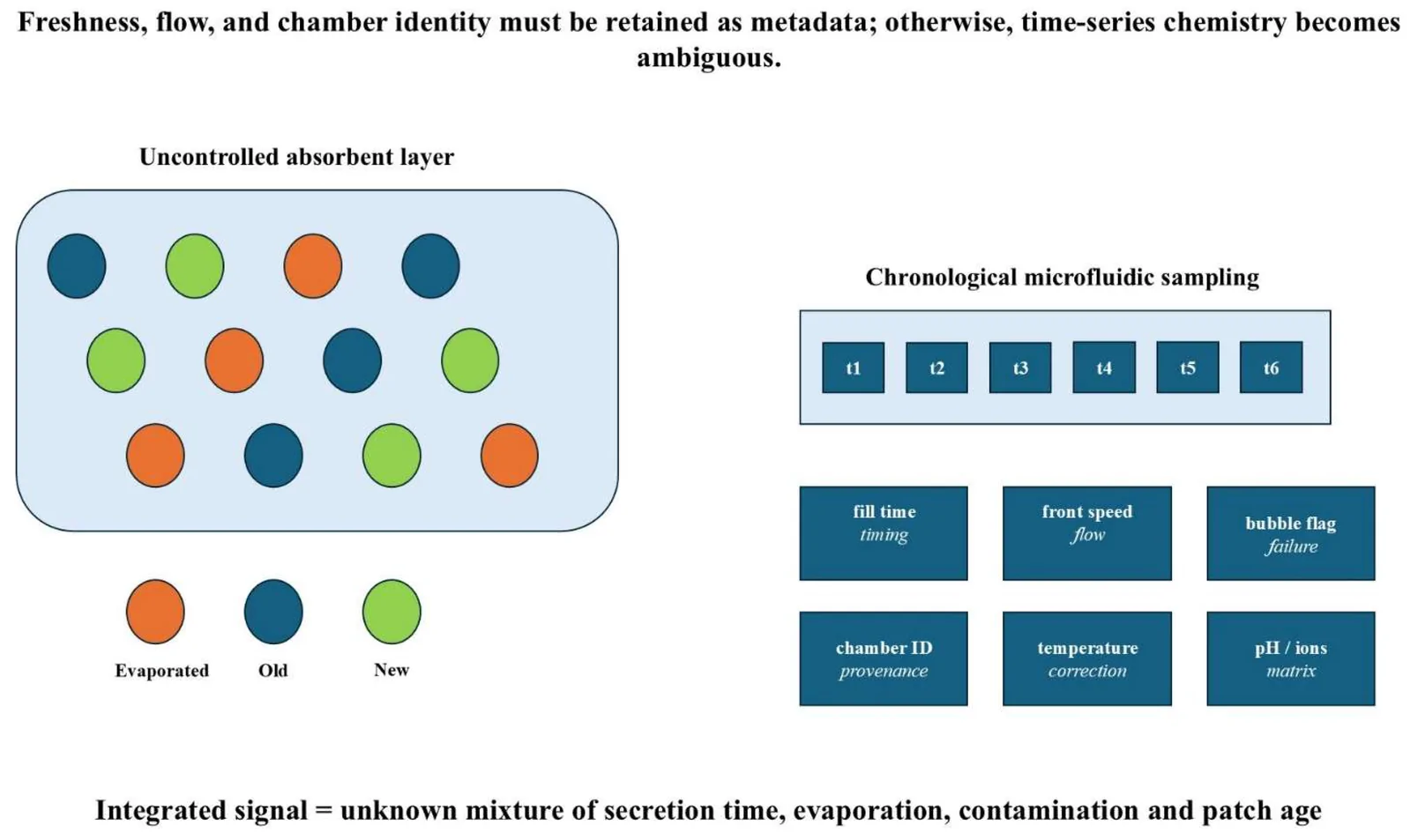
Sampling chronology as a source of signal and bias. Original schematic contrasting uncontrolled absorbent sampling with metadata-rich chronological microfluidic sampling [45,46].

The architecture of the sampling method will determine whether a time-based concentration value has meaning. Passive absorbent sampling methods are both simple and comfortable; however, they cannot separate old from new fluid, do not measure flow rate, do not allow evaporation, and do not allow the sampling time to be easily identified [45,46]. Soft microfluidics can route fresh sweat to chambers in chronological order, estimate the flow rate, and limit exterior contamination [47,48]. However, soft microfluidics introduces dead volume, air bubbles, leaks, and fabrication complexity. Iontophoretic stimulation may be used to measure resting sweat. However, it may alter local physiology, dilute the analyte, and present certain safety and power limitations. Microneedles allow direct access to interstitial adipose tissue; however, variability in microneedle length, microneedle activation, insertion pain, and the body’s response to the microneedle will become major considerations [49,50]. Droplet microfluidics and wet-chemical cartridges can be used to develop reagent-based assays that are fundamentally impossible to implement with dry electrodes; however, they will require substantial resources for reagent storage, reagent timing, and proper alignment [51]. Figure 4 illustrates why it is important to capture the sampling chronology explicitly. When a sensor is placed behind an uncontrolled absorbent sample pad, the signal will consist of an unknown mixture of fresh secretion, aged sweat, evaporated water, skin contamination, and patch age. If this signal is subsequently entered into a machine learning algorithm without including the metadata associated with the sampling event (e.g., sampling time), the algorithm may learn artifacts. Thus, chronological microfluidic sampling should not be viewed exclusively as an elegant feature of the device, but rather as a mechanism for determining data provenance. Attributes associated with sampling history, such as fill time, chamber identity, front speed, flow interruption, bubble indicators, and local temperature, should be retained in the analyte stream. Table 4 summarizes the technologies related to the sampling method and their potential implications for machine learning-ready data.

Recent human studies extend these sampling principles beyond short, exercise-dependent collection. A skin-interfaced microfluidic band combined chronological colorimetric timing with sequential sweat-pH and lactate assays, demonstrating that sampling time, anatomical site, local muscle recruitment, and fitness state are part of the biochemical interpretation [8]. Bioinspired graded microchannels and asymmetric membranes subsequently sustained low-volume sweat renewal for more than 48 h after a single carbachol-iontophoresis session and supported uric acid, xanthine, and alcohol monitoring in healthy participants and patients with gout [10]. A 2026 skin-conformal platform further used low-frequency ultrasound to deliver carbachol and generate sweat at rest, with integrated K+, pH, and uric acid sensing in a preliminary human evaluation [52]. These approaches reduce dependence on vigorous exercise but do not remove the need to report stimulation dose, collection history, local sweat rate, transit time, and the distinction between stimulated and naturally secreted sweat.

### 4.2. Matrix-Specific Interpretation

In addition to being attractive because they can be obtained via a non-invasive procedure, sweat samples are not concentrated blood specimens [39,40]. For electrolytes, sweat concentration may be primarily determined by sweat production rates and how well they are reabsorbed in the ductal glands. For lactate, the metabolism of individual sweat glands and exercise intensity can greatly affect its concentration in sweat. In addition, due to partitioning, binding, and time-lag effects, the analysis of cortisol or drugs based on sweat requires analyte-specific validation. Finally, with respect to nutrition, the low concentrations of many nutrients in sweat can pose significant challenges for collecting data on the regeneration of sweat receptors [53]. Based on this perspective, any claim associated with sweat should specify whether the output is representative of local sweat-ductal gland chemistry or the composition of total sweat loss, serves as an indicator of changes in systemic pathways, reflects a hypothetical wellness characteristic, or has been clinically validated as a marker. In addition, while interstitial fluid has a stronger correlation with systemic circulation than blood for many solutes, it does not have the same composition as blood. Lag and bias associated with molecular transport, local tissue perfusion, local tissue inflammation, and sensor tenure will be present. Subsequently, the success of glucose measurements in interstitial fluid should not be automatically generalized to lactate, alcohol, ketones, drugs, or hormones. Each analyte will require either an analytical compartment model or descriptive and quantitative data to link the interstitial traverses to the desired reference point [42,43]. Recent platforms further demonstrate why matrix-specific rather than automatically blood-equivalent claims are required. For chronic wounds, iCares used pump-free directional microfluidics to refresh exudate across NO, H_2_O_2_, O_2_, pH, and temperature sensors. It evaluated local multiparameter profiles in patients with chronic wounds and perioperative participants [11]. For tears, a 2026 wireless smart contact lens measured uric acid in participants including individuals with hyperuricemia or gout and used individualized lag profiles to construct lifestyle-informed prediction models [54]. Neither study establishes generic interchangeability with systemic concentrations; instead, each treats transport, lag, sampling history, and local physiology as part of the measurement model. Sparse blood sampling should aid the research effort; however, the reference times and label values must be accurately recorded. Other biological fluids, such as saliva, tears, and wound exudate, demonstrate that clinical utility is not dependent on an analyte having blood-like characteristics. For example, a pattern of wound pH/protease may have significant clinical utility in predicting the likelihood of local wound healing or infection but may not have any real relationship to serum. For example, sensors incorporated into mouthguards placed in the mouth may provide important information on a person’s training technique or their ability to take medication as prescribed, even though the sensors have no application in diagnostic testing or approval [55,56,57]. Similarly, the potential for a tear sensor to provide clinically important diagnostic information related to ocular disease does not require a relationship to the systemic concentrations of the respective biological substance(s) [58,59]. As such, the strongest wearable biosensor publications are those that explicitly delineate both the biological compartment being measured and the intended decision based upon the biomarker [60,61].

### 4.3. Breath Condensate and Multi-Matrix Sampling as Boundary Cases

When constructing a future framework for multi-matrix sampling, three labels should be assigned to each data stream: compartment (biological) identity, sampling state, and intended inference. The compartment identity (representing biological space) indicates which biological space the measurement most directly represents; the sampling state identifies whether the sensor is currently receiving new fluid, passively integrating old samples, receiving induced flow, or in a low-confidence period. The intended inference describes whether the measurement will be used as an indicator of tissue status, a surrogate for systemic variables, a control variable for monitoring experiments, or a quality-control variable for instrument calibration. Although these three labels are simple, they are powerful and can limit the number of invalid claims [15,44]. For example, a trend in pH measurements of local wound exudate should not be interpreted as equivalent to systemic acid–base status; sweat sodium (flux) should not be considered to be equivalent to serum sodium; and breath condensate should not be promoted as a quantitative plasma biomarker unless transport and comparator data are available to support this claim. Similar practical considerations apply to all matrices: for instance, a sweat patch that is not capable of determining whether it is sampling fresh sweat, old sweat, or evaporated residuals faces a comparable issue; similarly, a microneedle array that cannot assess the depth of insertion and quality of contact also provides a signal whose compartmental origin cannot be identified. Multi-matrix systems that may ultimately combine sweat, interstitial fluid, breath, skin temperature, motion, and symptoms must be designed not to create the false impression that multiple matrices automatically yield stronger evidence than a single matrix; each matrix must provide a specific explanatory function. For example, sweat may be used to estimate glandular secretory function; interstitial fluid may be used to provide systemic molecular context for physiological activity; breath may provide evidence of either respirable exposure/molecular metabolism; and the amount of motion may yield an estimate of physiological demand. The panel’s usefulness is realized when each of these functions is treated separately, rather than averaging them to produce an opaque score.

Breath condensate has been added to this framework, not because it is developed to the same extent as either sweat or interstitial-fluid measurement systems but because it demonstrates a critical boundary to the use of wearable systems for measuring biochemical compounds; that is, the sampled matrix will vary in the case of discontinuity and sensitivity to ambient conditions (environmental) and vary greatly in terms of the sampling-base device’s geometrical configuration. Mask-based systems and collector-based systems have the potential to provide access to volatile compounds, aerosolized particles, and condensed respiratory moisture [62,63]; the biochemical information that may be obtained from such systems includes airway inflammation, oxidative stress, metabolic state, and exposure. However, interpretation of the quantitative data from these systems may be problematic, since the ability of the device (i.e., the sampling apparatus) to condense particles will depend on the user’s breathing pattern, flow rate, humidity level, temperature, dead volume of the device, and the surface energy of the collecting surface; therefore, a concentration reported from the breath condensate sampled will be related to a characteristic of the device as well as of the airway. Thus, respiratory wearables should be defined as collection–assay systems, rather than as direct biochemical sensors [44]. EBCare provides a recent human example of this collection–assay architecture. Tandem cooling, automated microfluidics, electrochemical sensing, and wireless readout enabled continuous exhaled-breath-condensate analysis during daily activities and in participants with asthma, chronic obstructive pulmonary disease, or post-COVID-19 status [9]. This feasibility evidence does not eliminate breathing rate, condensation efficiency, ambient temperature, or dilution effects; it demonstrates that these variables must be measured or controlled as properties of the complete collection–assay system.

## 5. Recognition and Transduction: From Detection to Continuous Interpretation

The classification interface determines what types of molecules can be detected with a recognition element, how quickly the signal will be detected after the analyte has come into contact with the recognition site, and whether there will be a signal drift in the signal output, as well as whether or not the detection method allows the signal to be regenerated. Continuous glucose monitoring has greatly benefited from the development of enzymatic glucose oxidase chemistry; however, other metabolites (besides glucose) may not be as readily recognized using similar recognition methods. Many metabolites can still be detected enzymatically; these include lactate, alcohol, uric acid, glutamate, and many of the ketones. In addition, electrocardiograms (ECGs) can be recorded using ion-selective membranes. Hormones, cytokines, proteins, nucleic acids, nutrients, and drugs generally require some form of affinity recognition, molecular imprinting, immunoassay formats, or spectroscopic fingerprints for detection. All of these recognition-type methodologies serve to broaden the range of analytes that can be quantified; however, they may also introduce challenges related to reversibility, fouling control, and matrix-based validation [64,65]. Electrochemical transduction methods are the predominant technology currently used to develop biochemical wearable devices interfaced with the skin, due to their size, low power consumption, printable electrode fabrication, and suitability for electronic integration [66,67]. Amperometric sensors measure current generated in redox reactions; potentiometric sensors measure the potential difference generated across an ion-selective membrane; voltammetric methods resolve electroactive species; and impedance measures the binding process, including hydrating conditions, amount of fouling of the device, or amount of contact tissue between the sensor and/or the tissue in which it is implanted. All electrochemical signals will be adversely affected by temperature, pH, ionic strength, O_2_, interferents, electrode drift, or mechanical deformation [68,69]. Variable conditions must be measured and/or controlled, rather than treated as an afterthought. Affinity sensors also broaden the range of molecules that can be quantified. Aptamers are attractive candidates for developing affinity sensors because they undergo conformational changes upon binding to the target analyte [70,71]; these changes can be detected electrochemically [72,73,74]. Molecularly imprinted polymers can be used to develop synthetic binding sites that are long-lasting and economical to produce [75,76]. Antibodies and antibody fragments are generally highly specific; however, they lack the compatibility required for continuous, reversible measurement. Immunoassays, used with reagents to quantify proteins and cytokines, are very powerful tools; however, they are typically used only intermittently and do not provide continuous quantification. Organic electrochemical transistors and field-effect devices can amplify interfacial changes [77,78]; however, these changes reflect not only device physics but also the analyte binding to the device [79,80]. Optical and spectroscopic measurement methods, such as colorimetry, fluorescent spectrophotometry, electrochemiluminescence, or surface-enhanced Raman spectroscopy, provide multiplexing capabilities and chemical fingerprints of the sample; however, optical calibration and standardization of the substrate are required to obtain accurate results [81,82]. Regarding continuous monitoring of analytes, the critical aspect is not whether an analyte can be detected at a single point in time, but whether the recognition element provides a continuous, interpretable output. The following conditions may generate false physiological measurements: saturation of the binding site; irreversibly adsorbed analytes; degradation of enzymes used for recognition; leaching of electron mediators (in electrochemical sensors); photobleaching of the recognition element; pH shift; dehydration of the membrane; or delamination of the electrode. In the absence of sufficient control channels, no level of artificial intelligence (AI) can adequately protect against such detection errors. Devices that utilize redundant electrode arrays, internal standards, quality flags, and regeneration cycles can use modeling techniques to extend the device’s functional life and report measurement uncertainty. Table 5 outlines various transduction methods that may be used in wearable monitoring sensors.

### 5.1. Electrolytes and Enzymatic Metabolites: The Near-Term Core

Two major advances have occurred in the development of near-term core metabolic markers for non-glucose monitoring: (a) electrolytes and (b) enzymatic metabolic products. Sodium and chloride are useful in the context of sweat loss and cystic fibrosis; potassium and ammonium provide additional ionic information, while pH could serve as both a biomarker and a correction variable [68,69]. Lactate, uric acid, alcohol, and ketones have great potential, in part because there are enzymatic (or catalytic) means to measure these targets that will be relevant to the body [83]; additionally, these analytes will help educate the field regarding the integration of chemistry with context. For example, sodium in sweat without a sweat rate means little; conversely, lactate in the absence of exertion and with consideration of perfusion means even less; and uric acid in wound exudate might be more clinically relevant locally than from a systemic perspective. Care must be taken to avoid overstating the ability of electrolyte wearables to replace serum chemistry; for example, while sodium in sweat is representative of local secretion/reabsorption, it is not representative of serum sodium, and lactate in sweat does not automatically mean the same as lactate in blood when referring to sepsis or critical care [84]. An appropriate manuscript will state which of the following uses is planned: (i) hydration guidance; (ii) heat-stress risk; (iii) exercise physiology; (iv) local tissue metabolism; or (v) diagnostic replacement [66,85]. The claim boundary will determine the appropriate comparison in each example. For a hydration patch, an assessment of body mass change, whole-body sweat loss, and symptomatology (i.e., hurt vs. not hurt) is required. In contrast, for a diagnostic claim, clinical laboratory results are necessary to validate the electrolyte reading(s). For a performance patch, workload, perceived exertion, and recovery will be the performance metrics compared at baseline to determine whether the wearer is dehydrated relative to established hydration standards [40,86]. Human proof-of-concept studies have also extended wearable microneedle monitoring beyond glucose. A 2024 platform followed interstitial-fluid β-hydroxybutyrate for five hours after a ketone challenge and compared the resulting trajectory with blood measurements [87]. However, a subsequent 14-day randomized human evaluation found a progressive day-to-day decline in continuous ketone-monitor output in both ketone ester and placebo groups, consistent with a sensor-related long-wear effect [88]. In 2026, microneedle lactate biosensors were evaluated for continuous interstitial-fluid monitoring in critically ill hospitalized patients [89]. Together, these studies strengthen human and clinically aligned evidence while showing that short-duration accuracy cannot establish multiday stability, action-threshold performance, or clinical utility.

### 5.2. Affinity and Molecular Fingerprinting: High Value but Higher Risk

Conventional healthcare has little information on the scientific basis behind many hormones (e.g., cortisol, estradiol, progesterone, testosterone), drugs (e.g., L-DOPA, antibiotics), proteins (e.g., amino acids), cytokines, and nutrients (e.g., vitamins). While these targets can support physiology related to stress, fertility, pharmacokinetics, nutrition, and chronic disease management through the use of affinity or molecularly imprinted molecules, the time it takes to bind to a target may vary depending on whether binding is nonspecific, irreversible, or matrix-dependent. In addition, many tests may require continuous operation, such as those that rely on regeneration, competitive formats, microfluidic washing, periodic reagent delivery, or model-based interpretation of binding kinetics to maintain functionality [70,74]. One way to address the above-mentioned problems is to analyze samples using multiple spectroscopic techniques (e.g., SERS) rather than targeting a single receptor. By using time-stamped sweat samples combined with machine learning to measure metabolites (e.g., lactate, uric acid, tyrosine) and identify sweat-associated exercise-related metabolic profiles, tremendous potential exists to expand physiological coverage by enabling real-time sampling compared with traditional methods. Additionally, reliance on the previously mentioned measures will limit the precision of our quantitative findings to the extent that they depend on the reproducibility of the substrates, the uniformity of “hot spots,” preprocessing of the spectra, calibration transfer, sweat flow conditions during collection, and model validation. A SERS wearable, therefore, should not be viewed merely as an optical substrate or as a stand-alone assay but as an optofluidic and software-combined assay [81,82]. Recent human studies also illustrate the expanding molecular reach and continuing claim boundary of affinity and multiplex platforms. Stressomic combined controlled sweat induction with chronological microfluidic valving to profile cortisol, epinephrine, and norepinephrine during physical, psychological, and pharmacological challenges [12]. A fully integrated sedentary-sweat patch combined L-DOPA, ascorbic acid, and glucose channels with on-device processing and was evaluated in healthy volunteers and patients with Parkinson’s disease [13]. These studies support time-resolved endocrine and pharmacokinetic feasibility, but neither independently establishes endocrine diagnosis, disease-progression assessment, autonomous dose adjustment, or sensor-guided improvement in patient outcomes.

### 5.3. Dynamic Interface Validation: Stability, Regeneration, and Matrix Challenge

A practical reporting convention involves reporting three types of calibration states: “fresh”, “aged”, and “used”. Differences among these three states often determine whether a specific sensor is ready for practical use. A sensor that appears to perform well at the time of manufacture but loses 50 percent of its functionality after 1 h of exposure to moisture may retain some value as a disposable exercise patch [90,91]. However, it is still not considered a viable alternative to multiday clinical monitoring. Conversely, a sensor with moderate sensitivity, a predictable lifetime, built-in self-diagnostics, and a clearly defined replacement will be of practical use. Continuous biochemical measurements should benefit from both reliability and interpretability to the same level as analytical novelty. Regeneration is also worth consideration, as many high-value targets, similarly to glucose, will be detected based on their chemical affinity rather than their enzymatic activity. An aptamer or molecularly imprinted interface will be able to support multiple measurements only if the metabolic binding and unbinding of the analyte (i.e., target) remains fast-acting and stable under body-worn conditions. Electrocatalytic, chemical catalytic, thermal catalytic, optical, or microfluidic processes can all be utilized to regenerate sensors; however, each process adds its own set of operational burdens, e.g., energy requirements, reagent storage, safety considerations, sample dilution, and interfacial fatigue. Therefore, the relevant performance metrics of sensors comprise not just the limit of detection but also: (1) the number of repeatable binding–reset cycles, (2) recovery to baseline after each cycle, (3) time to initial response and reset, and (4) drift in calibration over the entire intended wear timeframe [92,93].

Matrix challenge experiments need to be designed with a substantial degree of plausibility, rather than merely following classical interferent panels. For example, a sweat-cortisol sensor needs to withstand variability in pH, salt content, urea, lactate, skin oils, sunscreen residues, and temperature, all of which are subject to change on a person’s skin. Likewise, a microneedle drug sensor needs to withstand protein adsorption, local inflammatory markers, ionic concentrations of tissue fluid, and mechanical micro-motion. Furthermore, when evaluating a SERS sweat patch, the evaluator should subject the sensor to variations in substrates, low-flow drying, mixed old/new sweat, and background shifts in spectra. Reporting a sensor’s selectivity across several molecules at a fixed concentration does little, if anything, to capture the full complexity of operating a wearable sensor; therefore, the best rendition of a manuscript would transparently clarify where a sensor fails, how its diagnostics disclose that failure, and how the algorithm treats those failures. One major consideration when differentiating between an attractive sensor chemistry and a commercially viable continuous sensor is dynamic stability. Many demonstrations for targets other than glucose will generate strong calibration curves when assessed in buffered solutions or through short-duration application of spiked indices of artificial sweat. While those evaluations will yield useful results, they do not establish whether the sensor interface will maintain chemical stability after multiple wet/dry cycles, mechanical deformation, biofouling, and variation in solution ionic strength. Therefore, a Q1-level assessment would consider the evaluation of stability to be a principal evaluation measure, as opposed to an incidental experiment, with the intention of developing a principal assertion. Regarding enzymatic isolation, the laboratory must demonstrate that there is no loss of enzymatic activity or retention of mediator compounds, nor can the sensor’s continued operation be dependent on the presence of oxygen, hence the need to evaluate membrane permeability and the effects of reducing-agent- or circuitry-based redox-interfering substances. For potentiometric measurements, assessors should quantify reference-electrode drift, ionophore leaching, the system’s hydration history, and junction potentials. For affinity systems, researchers should state the ability to demonstrate reversible binding, recovery of baseline values, the ability to resist the effects of nonspecific adsorption, and the capacity to regenerate the biosensor using real biological matrices or realistic matrix surrogates [75,94].

## 6. Multianalyte Panels Beyond Glucose: From Analyte Menus to Clinical Questions

A key problem associated with the development of wearable biosensors is that many studies have created multianalyte test panels without an underpinning rationale grounded in clinically relevant questions, rendering the multiplexing of analytes of little value. A lactate monitor is considerably more informative when combined with exercise, heart rate, skin temperature, and sweat flow [95]; for example, optimal hydration guidance supports the interpretation of sodium, chloride, sweat rate, thermal strain, and environmental heat together [40,86]. In endocrine profiling, data must be collected related to clock time, sleep quality, sources of stress, and matrix metadata (e.g., blood serum). Therapeutic drug monitoring will require consideration of dosing timing, dosage form, renal/hepatic status, and the number of pharmacokinetic comparators; therefore, a therapeutic drug panel is ultimately driven by the decision to be made, rather than simply by the most convenient combination of electrodes. Figure 5 separates two related but non-equivalent dimensions of translation. Panel A presents the comparative biofluid-specific readiness map based on analyte accessibility, recognition chemistry, sampling control, matrix behavior, and the plausibility of the intended claim. Panel B adds a temporal and quantified map of published human evidence. For each analyte–biofluid–use-claim combination, the color indicates the highest Human Evidence Level achieved, the symbol area represents a conservative lower-bound class for the number of independent on-body or direct in situ human studies, and the horizontal span extends from the first to the most recent qualifying report.

The resulting evidence landscape demonstrates that publication volume does not itself establish clinical maturity. Electrolytes, transdermal alcohol, lactate, and cortisol have accumulated the largest human evidence bases, but only transdermal alcohol reaches HEL4, and only for the narrow context of sensor-contingent alcohol-reduction interventions. The strongest wearable claims for lactate, uric acid, ketones, cortisol, sex hormones, amino acids, vitamins, drugs, cytokines or proteins, and wound markers remain at HEL2 because independent claim-specific validation of a locked output or prospective clinical-utility evidence is absent. The combined labels in Panel A are therefore separated in Panel B so that alcohol evidence does not elevate ketones, lactate evidence does not elevate uric acid, and cortisol evidence does not elevate the more limited sex-hormone literature. Table 6 identifies the level-setting analyte and claim, the evidence required to reach the next HEL, and the principal unresolved translation barriers.

Figure 5 was constructed using a context-of-use-specific evidence rubric rather than a universal analytical cut-off. Each biomarker-family–biofluid pairing was evaluated across five domains: (i) target-fluid availability and physiological-range characterization (F); (ii) matrix-specific analytical validity and recognition-layer stability (A); (iii) controlled sampling and integrated on-body performance (W); (iv) time-aligned human evidence linking the target-fluid signal to the stated physiological or clinical endpoint (V); and (v) a defined decision or action pathway (U). Each domain was scored as 0 (no direct evidence, evidence limited to buffer or artificial samples, or evidence derived from another biofluid), 1 (exploratory evidence in the target matrix or a pilot human setting), or 2 (advanced and replicated evidence under conditions relevant to the intended use). The component scores were summed to provide an auditable score, S = F + A + W + V + U, ranging from 0 to 10. The domains were informed by biofluid-specific sampling physiology, end-to-end wearable design, and the fit-for-purpose distinctions among verification, analytical validation, and clinical validation described in the V3 and IMDRF frameworks; however, the scoring anchors and classification gates were developed specifically for this review [5,6,7,15,18,21,25].

Because these domains represent sequential links in the measurement chain, the final category was determined using non-compensatory stage gates rather than the total score alone. A pairing was classified as Immature when direct target-fluid evidence or relevant-matrix analytical evidence was absent (F = 0 or A = 0). It was classified as Promising when target-fluid and analytical feasibility were present but integrated wearable sampling, human endpoint evidence, or a defined use pathway was absent (W = 0, V = 0, or U = 0), or when S was no greater than 5. Near-term status required evidence in all five domains, an S of at least 6, and human on-body evidence, while at least one domain remained preliminary. Use-case validation-ready status required a score of 2 in all five domains. For systemic-surrogate claims, temporally aligned reference-fluid evidence was required; for local applications, such as wound or ocular monitoring, an appropriate local biological or clinical endpoint was accepted. Evidence obtained from one biofluid was not extrapolated to another.

The classifications represent a time-dependent synthesis of the literature included in this review and not a universal technology-readiness or regulatory scale. “Use-case validation-ready” denotes sufficient evidence for a narrowly defined application and does not indicate regulatory authorization, biomarker qualification, equivalence to blood measurement, or suitability for autonomous clinical decision-making. For grouped biomarker families, analytes should be scored individually; where they cannot be separated in the figure, the more conservative tier should be displayed.

### 6.1. Representative Literature Landmarks and Quantitative Evidence Map

The following examples are not an exhaustive history of wearable biosensing. They are selected because each contributes a design lesson to the transition from a flexible sensor to an intelligent biochemical system. Imani, Bandodkar, Mohan, and co-workers showed that a biochemical lactate channel and electrocardiography could be co-fabricated and acquired simultaneously on the skin [95]. Nightingale and co-workers demonstrated wearable wet chemistry using droplet microfluidics and continuous dermal sampling [51]. Tehrani and co-workers advanced microneedle interstitial-fluid sensing toward integrated wireless monitoring of multiple metabolites [49]. Wang and co-workers expanded sweat sensing toward nutrients and metabolites using regenerated molecularly imprinted interfaces [93]. Ye and co-workers reported a wearable aptamer nanobiosensor for estradiol with autonomous sweat induction and multivariate correction [98]. Spinelli and co-workers demonstrated field-oriented hydration monitoring with haptic feedback and ground-truth correlations [86]. Jeon and co-workers introduced chronoepifluidic SERS for label-free multiplexed profiling of sweat metabolites [82]. Choi and associates presented a new advancement in multimodal physical and chemical (physicochemical) monitoring by implementing printed neuromorphic processing [101]. The design of Table 7 is intentionally based on evidence fields instead of citation quality. The variable characteristics include: 1. What biofluid was evaluated? 2. Analytes targeted? 3. Human or field-sourced data reported? 4. Which quantitative or qualitative characteristics supported the claims made above? 5. What translational lessons may be derived from the described study? To assess the effect of the sensor’s sensitivity, a mature review must assess whether the study identified sampling issues, operated outside controlled laboratory conditions, aligned with the comparator, included a diverse range of subjects, and demonstrated the potential clinical utility of the results.

**Figure 6:**
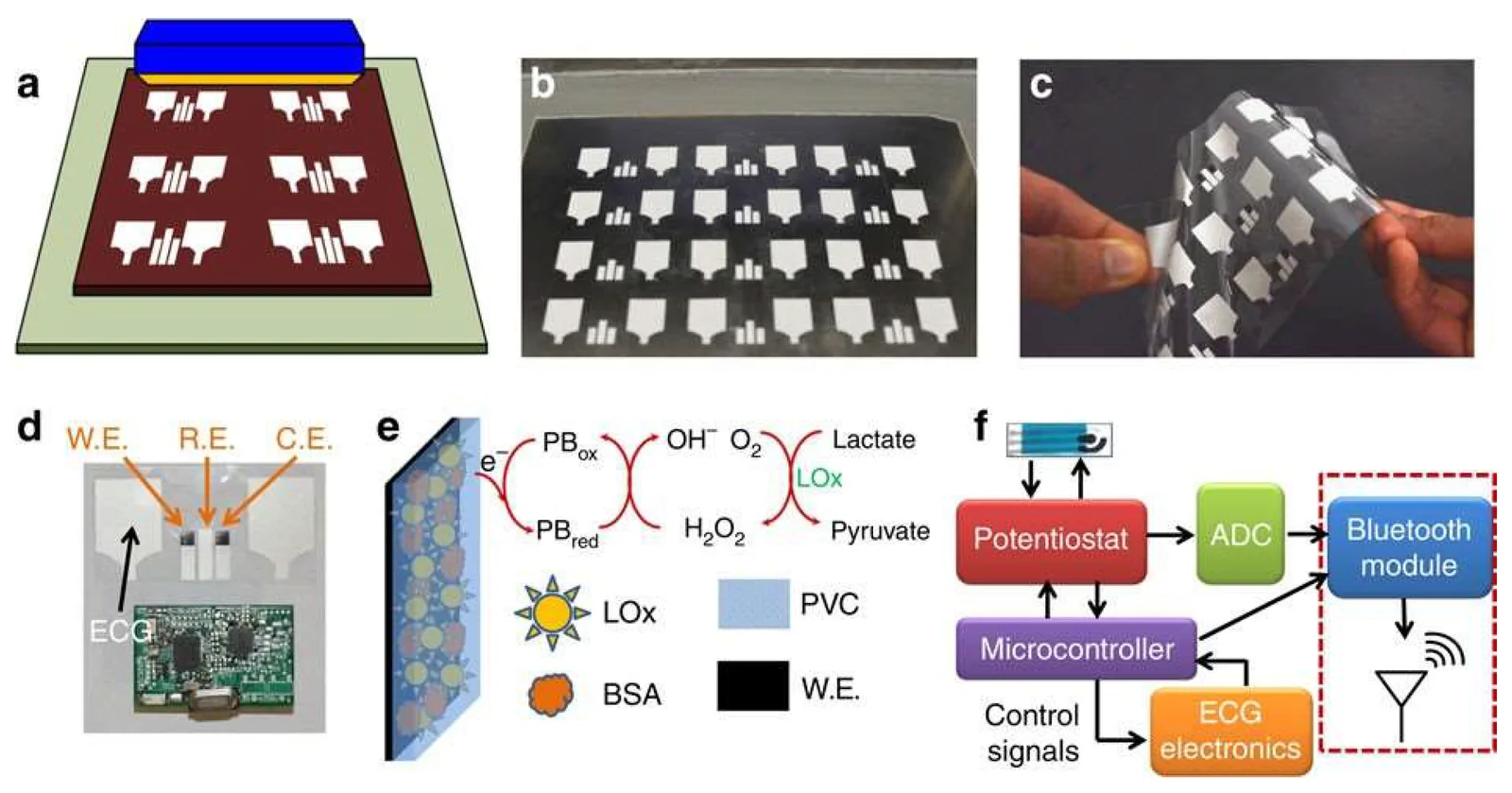
Representative hybrid biochemical–electrophysiological wearable platform for simultaneous lactate and electrocardiography monitoring. Reproduced from Imani et al., *Nature Communications*, 2016, under the Creative Commons Attribution 4.0 International License (https://creativecommons.org/licenses/by/4.0/). Displayed with resizing only; no scientific content was modified [95]. (**a**) Schematic showing the screen-printing process. (**b**) Image of the Chem–Phys printing stencil. (**c**) An array of printed Chem–Phys flexible patches. (**d**) Image of a Chem–Phys patch along with the wireless electronics. (**e**) Schematic showing the LOx-based lactate biosensor along with the enzymatic and detection reactions. (**f**) Block diagram of the wireless readout circuit.

Hybrid chemical–physical sensing is an early bridge between isolated biochemical patches and AI-ready multimodal systems. Figure 6 illustrates a skin-worn lactate–electrocardiogram platform. The main lesson is not only that two modalities can coexist but also that co-located streams allow downstream algorithms to interpret biochemical change in the context of physiology [95].

**Figure 7:**
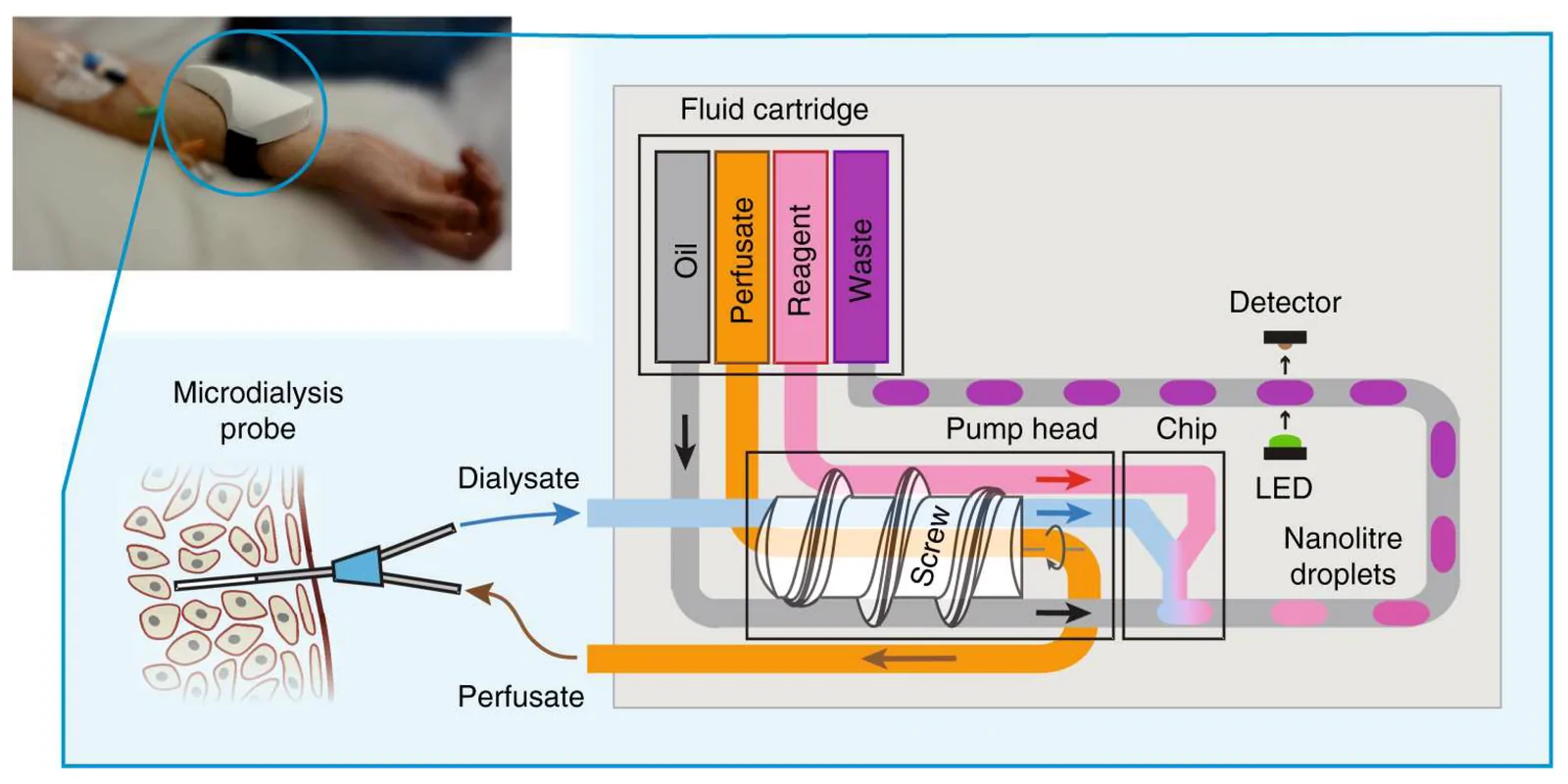
Wearable droplet microfluidic-based sensor for repeated biomolecule monitoring in dermal tissue. Reproduced from Nightingale et al., *Nature Communications*, 2019, under the Creative Commons Attribution 4.0 International License (https://creativecommons.org/licenses/by/4.0/). Displayed with resizing only; no scientific content was modified [51].

Droplet microfluidics extends wearable biosensing beyond dry electrodes, enabling repeated wet-chemical assays [51]. Figure 7 shows how controlled sampling and in situ reaction timing can be miniaturized into an autonomous wearable format. Such architectures are important for biomarkers that cannot be measured reliably by direct reagentless electrodes.

### 6.2. Readiness Tiers for Beyond-Glucose Targets

The two panels of Figure 5 answer different questions. Panel A describes comparative translational readiness based on biofluid accessibility, concentration range, recognition chemistry, sampling control, matrix robustness, and the plausibility of an actionable claim. Panel B describes only the maturity and volume of published human evidence using the HEL framework. Consequently, a biomarker may have a large body of human literature but remain at HEL2 because the studies do not independently validate a locked claim. In contrast, a technically promising biomarker may have only a few feasibility studies. Neither classification substitutes for the non-compensatory AI-readiness criteria in Table 2 . This tiering is not meant to discourage ambitious targets. It is meant to align claims with evidence and help authors decide whether a manuscript should frame a result as a sensor demonstration, a physiological study, a digital biomarker candidate, or a clinically actionable tool [17,18]. The readiness tiering of a multimodal target establishes the significance of multimodal targeting. Multimodal targeting uses multiple control variables or contextual information to improve the interpretability of high-risk biomarkers. For example, in the case of a wound cytokine output readout, it would provide greater predictive value when interpreted alongside pH, temperature, exudate volume, and dressing age. Additionally, a sweat-cortisol readout would be more interpretable when information is available on the circadian time of day, sleep quality and duration, previous exercise, current sweat flow rate, and ambient temperature. A drug sensor or pharmacometric output becomes actionable when comparable covariates are included, such as drug dosage time, drug formulation, and renal function. Therefore, when constructing a panel for a multimodal target, it is essential to pair ambitious molecular targets with robust contextual variables to prevent overinterpretation of high-risk biomarkers. The high-risk tier/research tier includes a broad panel of cytokines, multi-hormone endocrine diagnostics, low-abundance protein analytes, and many disease-specific analytes found in sweat or tears. Selecting analytes with these characteristics can provide unique predictive insights in the future. Still, most require some background removal, amplification, capture, washing, and/or periodicity [29,30]. Therefore, it is important to recognize that continuous action of a reagent-free sensing modality is not always a valid requirement for all analyte types. For example, when using a periodic microfluidic immunoassay repeated every 30 or 60 min, there is a greater likelihood of obtaining a sample value that is safer and more valid than a result from an alleged-continuous invalid trace with no surface-reaction measurements. Therefore, when measuring these targets, it is more accurate to state the greatest potential for local changes in tissue conditions or for exploratory molecular phenotyping than to state laboratory diagnostic replacements.

The intermediate tier includes cortisol, L-DOPA [102], select antibiotics, caffeine, amino acids, vitamins [103,104], and some reproductive hormones. The greatest advantage in this tier is that the relationships among these analytes may directly inform clinical or behavioral decisions regarding stress physiology, therapeutic drug monitoring, medication adherence, nutrition, and the rhythmic functioning of the endocrine system [105,106]. However, the key barriers to establishing the clinical validity of these analytes relate to the lower concentrations of these analytes; the development of stronger matrix dependence and protein-binding effects; the reversible nature of some types of receptor binding; the need for dosing or circadian datasets; and the degree of uncertainty regarding the analyte concentration thresholds [107]. Publications discussing any intermediate-tier analytes should focus only on results from on-body sensing technology, such as local continuous patches, to assess Clinical Readiness. Publications should specifically address dynamic validation, comparisons of timing with other methods, baseline personalization, and inherent uncertainty for each analyte and against other on-body comparators. The short-term tier includes electrolytes, pH, sweat rate, lactate, urine uric acid, and selected alcohol or metabolite measurements. The benefit associated with these analytes is the relative maturity of the electrochemistry or optical chemistries that will allow for quantification at concentrations that are accessible in sweat and interstitial fluids, as well as being within easily understood/explained physiological contexts, such as exercise, hydration, gout risk, oxidative stress, and/or exposure. Caution must also be considered with respect to sweat lactate not equaling blood lactate in individuals and sweat sodium not equaling free serum sodium in individuals. In addition, the physical/metabolic state of the person supplying the sweat will affect whether uric acid reflects local secretion or oxidative stress. However, when taken together, these types of analytes can support strong translational studies if the intended use of each analyte has been clearly communicated and a realistic alignment of comparative measures has been established.

## 7. Flexible Materials, Biointerfaces, and System Integration

Mechanical compliance is a vital but not sufficient requirement for a soft wearable chemical sensor to maintain a stable chemical interface while stretching, sweating, shedding skin cells, changing temperature, and hosting microbes over time [108,109]. Each material used must meet conflicting criteria: provide adhesive properties without causing irritation, direct fluid flow without contamination, encapsulate electronics while enabling access to biofluids, protect enzymes while allowing diffusion, and be comfortable for hours or days. The diversity of materials available for sensor development includes elastomers, hydrogels, fabrics, temporary tattoos, printed carbon and metal inks, liquid metals, conductive polymers, nanomaterials, and stretchable interconnects [90,91]. They do, however, create new modes of failure. Hydrogels are the most prominent example because they encompass both the biological and electronic domains and allow for the maintenance of hydration, the immobilization of enzymes, a reduction in mechanical mismatch, the storage of reagents, and the provision of ion-conducting interfaces. Their major disadvantages include swelling, dehydration, leaching, microbial growth, and batch-to-batch variation. Elastomers enable soft microfluidics for encapsulation but can absorb hydrophobic molecules, diminishing analyte recovery [110]. Fabrics provide comfort and scalability but can also lead to increased roughness, variable wicking properties, and laundering issues [111]. Temporary tattoos are both low-profile and conformal [112], but due to their limited fluid capacity and low durability, they are not suitable for long-term use. Microneedle arrays provide direct access to interstitial fluid; however, their production requires that the microneedles be mechanically strong, biocompatible, sterile, and capable of controlling the rate of insertion into the skin [49,113].

Recent platforms further clarify the distinction between mechanical novelty, integrated analytical performance, and clinical validation. A stretchable ionic–electronic bilayer hydrogel enabled in situ measurement of solid-state epidermal lactate and cholesterol in small human cohorts. Still, the output represented epidermal areal density rather than an interchangeable blood concentration [114]. A battery-free multimodal stress platform combined reusable sweat-cortisol sensing, physiological channels, NFC-powered sweat induction, rapid sample renewal, and participant-level evaluation [99]. A 2026 smart ring compressed passive osmotic sweat extraction, configurable biochemical channels, wireless electronics, and approximately 12 h of operation into a compact daily-wear format [14]. These examples constitute important integration and human-feasibility evidence, but their form factor or mechanical robustness does not itself demonstrate clinical validity or utility.

Elegant chemistry is often where the system’s integration fails. A wearable requires not only power but also communication, memory, amplification, filtering, signal digitization, firmware, packaging, and user interaction [21,22]. Near-field communication supports either battery-free or low-power operation, and limited long-distance data transfer can be accomplished using this technology. Bluetooth Low Energy supports real-time data streaming. However, it requires battery power and incorporates cybersecurity considerations. Power harvesting from sweat, motion, heat, and biofuel cells is attractive [115]; however, overall power consumption and reliability should be weighed against the benefits. In clinical use, power failure is not only a problem for an engineer but also a potential cause of missing data and a safety event. Anti-fouling and biocompatibility should be considered fundamental design criteria. If present, skin oils, proteins, salts, cells, cosmetics, and environmental debris will all foul electrodes and microchannels. Proteins and tissue reactions will also alter the diffusion of drugs into the interstitial fluid and onto electrode surfaces. Strategies to reduce fouling include zwitterionic coatings, hydrophilic barriers, size-exclusion membranes, sacrificial layers, controlled flow, periodic electrochemical cleaning, and algorithmic drift detection. Although algorithmic correction can help to address the issues associated with the use of chemical sensor materials, it should not serve as a replacement for robust materials [16,91]. Ideally, the best systems will combine design elements that physically prevent failures, redundant controls, and clearly defined methods to detect failures. Table 8 summarizes the material choices for flexible biochemical wearables.

## 8. AI-Ready Data Architecture and Sensor Fusion

**Figure 8:**
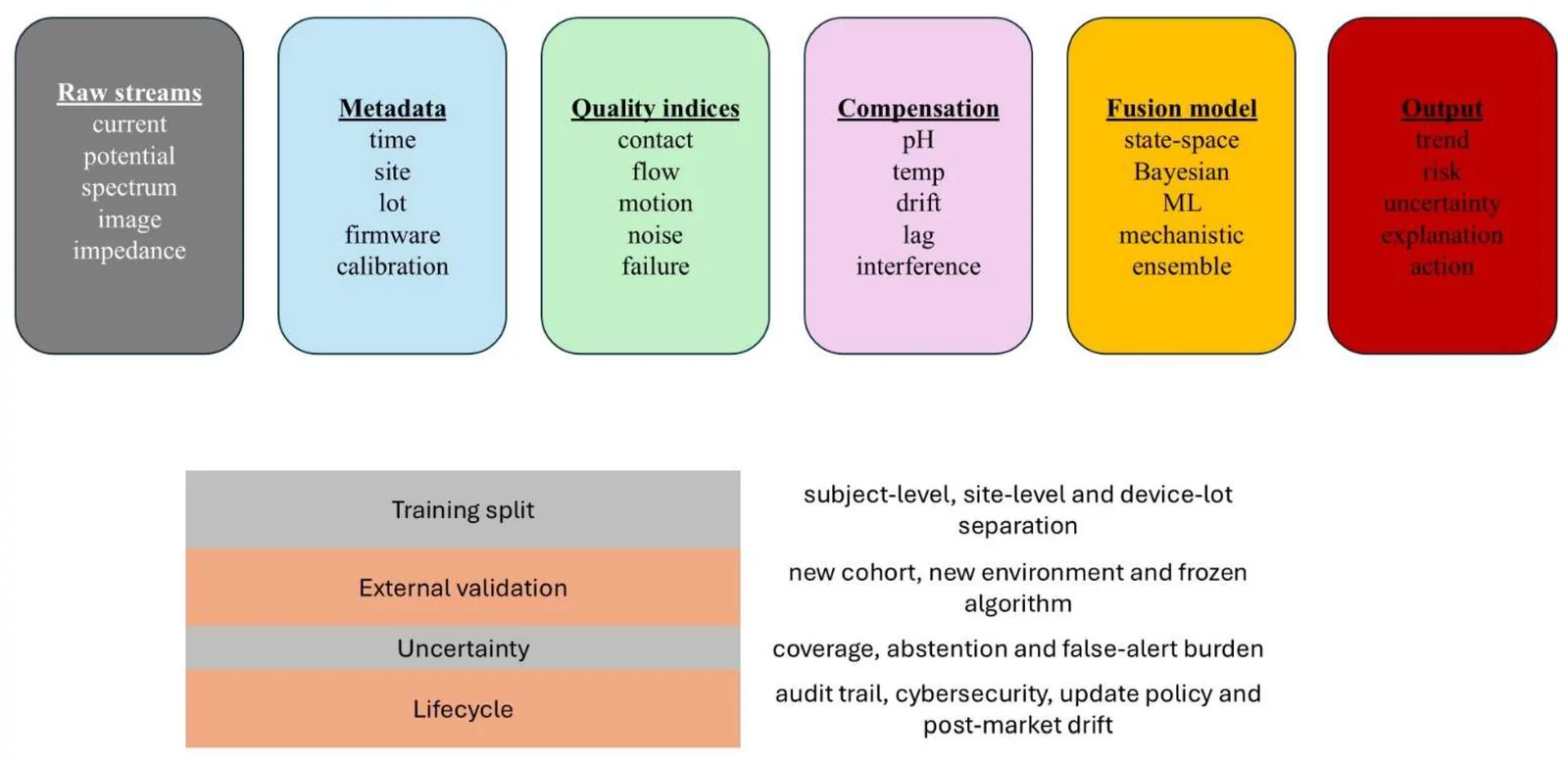
AI-ready data package and sensor-fusion pipeline. Original schematic separating raw streams, metadata, quality indices, compensation, fusion, and governed output [116,118].

Artificial intelligence is most useful in wearable biochemical sensing when it addresses problems created by continuous, noisy, heterogeneous, and context-dependent data. These problems include denoising, artifact detection, drift compensation, calibration transfer, temporal alignment, missing-data handling, personalized baseline estimation, event detection, risk prediction, and decision support [116,117]. It is important to note that there are no conditions under which the same model can be used; therefore, state-space models may outperform deep networks for lag correction when data are limited. It may be advantageous to use a Bayesian filter when uncertainty needs to be expressed. Training and validating diverse, well-labeled datasets are important for successfully using a transformer for long multimodal sequences. An AI-ready data package and fusion pipeline are shown in Figure 8. The following principles are outlined for raw data streams: First, preserve the original raw streams for as long as possible, and do not collapse them into higher-level summaries until necessary. Second, all measurements will include accompanying metadata (including device age, sampling states, temperature, pH, motion); current value, in isolation, will not be a reusable data point without device age, sampling conditions, temperature, pH, motion, calibration, and signal quality. Third, uncertainty will be first-class output. For continuous wearables, there will be instances where an analyte is rising, instances where it is stable, and instances where there is insufficient data for interpretation. Distinguishing all of these states is critical for clinical safety [17,18].

There are multiple levels of sensor fusion. In the realm of signal-level sensor fusion, raw or minimally processed signals are fused. An example would be taking an electrical lactate signal and applying corrections based on both temperature and pH. Feature-level sensor fusion combines derived descriptors (e.g., slope, variability, sweat-rate-normalized flux, heart-rate zone, and activity class). In contrast, decision-level sensor fusion combines outputs from independent models (e.g., metabolic-stress risk, dehydration risk, and sensor quality score). The best solution often uses a hybrid approach from each level, e.g., a dehydration model that combines physics-based equations to estimate sweat loss with learned relationships among sodium, skin temperature, movement, and individual learning history. A major risk is confounding; machine learning models could learn to recognize artifacts of the device (e.g., age, gender, trial protocol, example activities) instead of physiologic responses (e.g., if every high-lactate example comes from a treadmill session performed by a young man using a new device, that model will not generalize to an older man walking outdoors wearing a device that has partially dried sticky pads). Therefore, external validation, leave-one-subject-out testing, device-lot stratification, variation by body site, and real-world testing are all required [117,118]. Performance metrics must include not only average error or the percentage of correct classifications but also calibration error, affinity ranges, uncertainty by the threshold method (i.e., the first method to fail), false-positive reporting rate, and missing proportions [24,116]. Table 9 summarizes an AI-ready data package for multimodal wearable sensors, and Table 10 lists sensor-fusion tasks and validation metrics.

**Figure 9:**
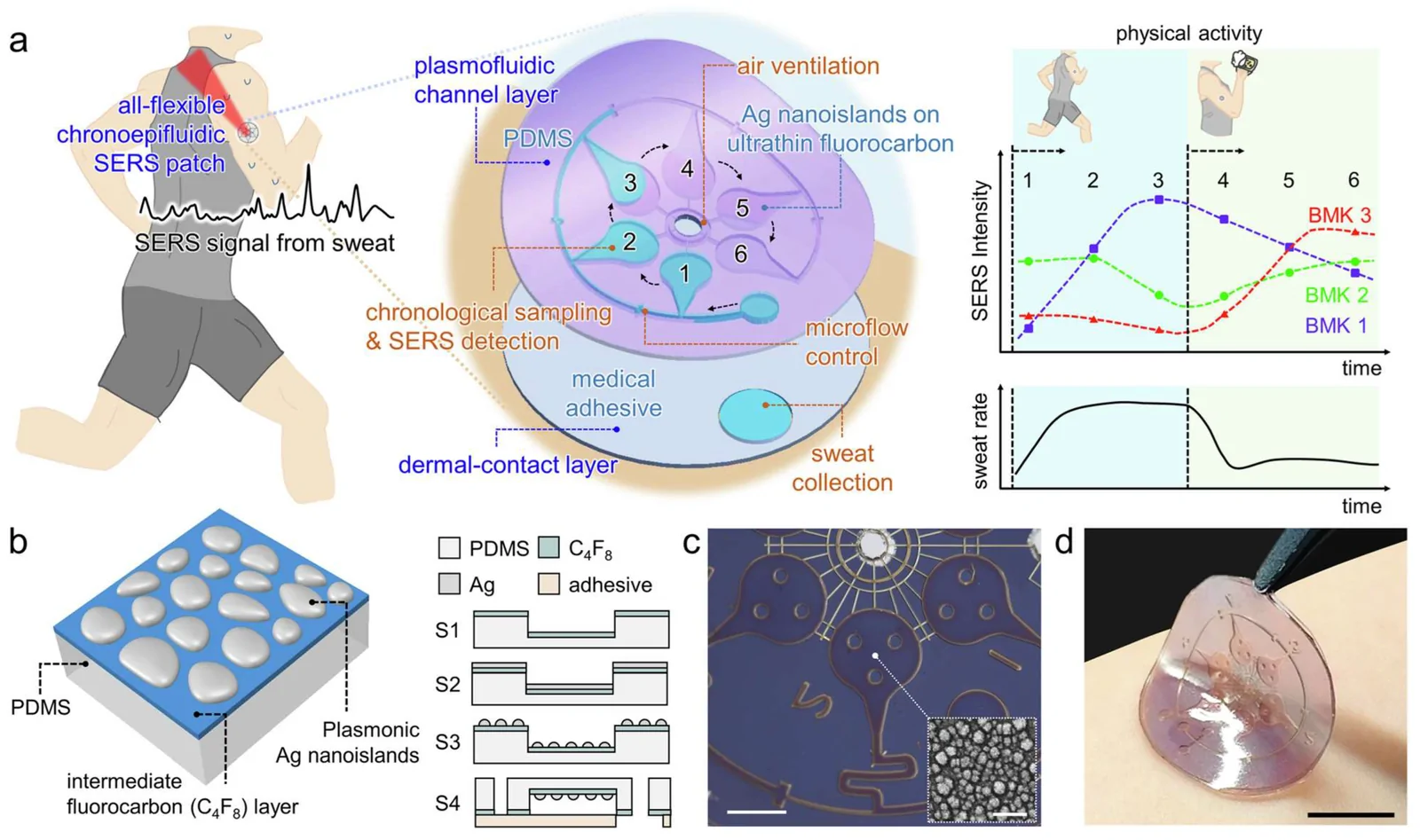
All-flexible chronoepifluidic nanoplasmonic patch for chronological sweat sampling and SERS-based label-free metabolite profiling. Reproduced from Jeon et al., *Nature Communications*, 2025, under the Creative Commons Attribution 4.0 International License (https://creativecommons.org/licenses/by/4.0/). Displayed with resizing only; no scientific content was modified [82]. (**a**) Schematic illustration of an all-flexible chronoepifluidic SERS patch (CEP-SERS patch) comprising plasmofluidic channel layer (PCL) and dermal-contact layer (DCL). PCL includes an all-flexible plasmonic SERS substrate and a chronoepifluidic sweat sampler. The flexible SERS substrate features plasmonic nanoislands on ultrathin fluorocarbon-coated PDMS membrane using low-temperature solid-state dewetting of thin silver film. The sweat sampler sequentially collects the sweat via the microfluidic channel with capillary bursting valves, which spatially separates the sweat over time. DCL interconnects the PCL on the skin for stable sweat collection, which contains medical adhesive with a sweat collection port. The CEP-SERS patch provides conformal contact on human skin and label-free sweat profiling of diverse metabolites from sequentially sampled sweat. (**b**) Schematic illustration of micro and nanofabrication for the CEP-SERS patch. The device fabrication includes fluorocarbon coating (S1), thermal evaporation of Ag (S2), low-temperature solid-state dewetting (S3), and microfluidic encapsulation (S4). The ultrathin fluorocarbon film effectively dewets the thin metal film on the sweat sampler, resulting in plasmonic structures with strong electromagnetic hotspots, which facilitate highly sensitive SERS analysis. Optical images of (**c**) the PCL (Scale bar: 1 mm) with an inset SEM image of Ag nanoislands (Scale bar: 100 nm) and (**d**) the CEP-SERS patch on the skin (Scale bar: 10 mm).

Spectroscopic and label-free wearable approaches can increase molecular multiplexity when paired with controlled sampling and machine learning calibration. Figure 9 provides a representative chronoepifluidic SERS architecture. The figure is placed here because it illustrates a central AI-ready principle: the model is inseparable from the substrate, microfluidic chronology, and spectral preprocessing [81,82].

### 8.1. Calibration, Personalization, and Leakage Prevention

Calibration is the connection between analytical chemistry and the clinical interpretation of results. Various calibration methods for chemical sensors are used to improve sensor performance, including factory calibration, one-point user calibration, and periodic recalibration. Different analytes require different calibration strategies, and it is necessary to determine whether reference materials are available. This document describes the calibration strategy that must be used, depending on the intended use and the quality of the reference materials. Relative calibration change may suffice for monitoring wellness trends at a personal level. Still, absolute calibration accuracy, traceability, and defined action thresholds will be required for therapeutic drug dosing or disease diagnosis [119,120]. Personalization may improve performance, but it will raise governance issues regarding that performance. Personalization may also mask sensor degradation or lead to inequitable performance, depending on the quality of the data used to personalize the model. AI-ready systems should maintain unique personal physiological baseline data, separate from unique device-specific performance drift. These systems should have mechanisms for storing calibration history, reporting uncertainty, and regularly auditing performance for model updates. Personalized results should not be produced in a “black box” manner. It is very common for physiological sensor data to leak into performance. Data time segments produced from the same subject or device during the same protocol and/or session will be highly correlated. Randomly splitting windows in a dataset to develop an accurate model will yield very good test results on the new data; however, the model would fail to perform well with any new user. Minimum subject-level validation should be completed on the subject; however, site-level validation, device-lot-level validation, and external cohort validation are needed to establish stronger evidence for general applicability across users or the population. All statistical models used to analyze repeated time periods must account for the within-subject correlation between time segments of varying durations. Treating thousands of windows collected from ten subjects as independent observations will greatly overestimate their precision [117,118].

### 8.2. Edge Intelligence, Neuromorphic Processing, and Privacy-Preserving Learning

**Figure 10:**
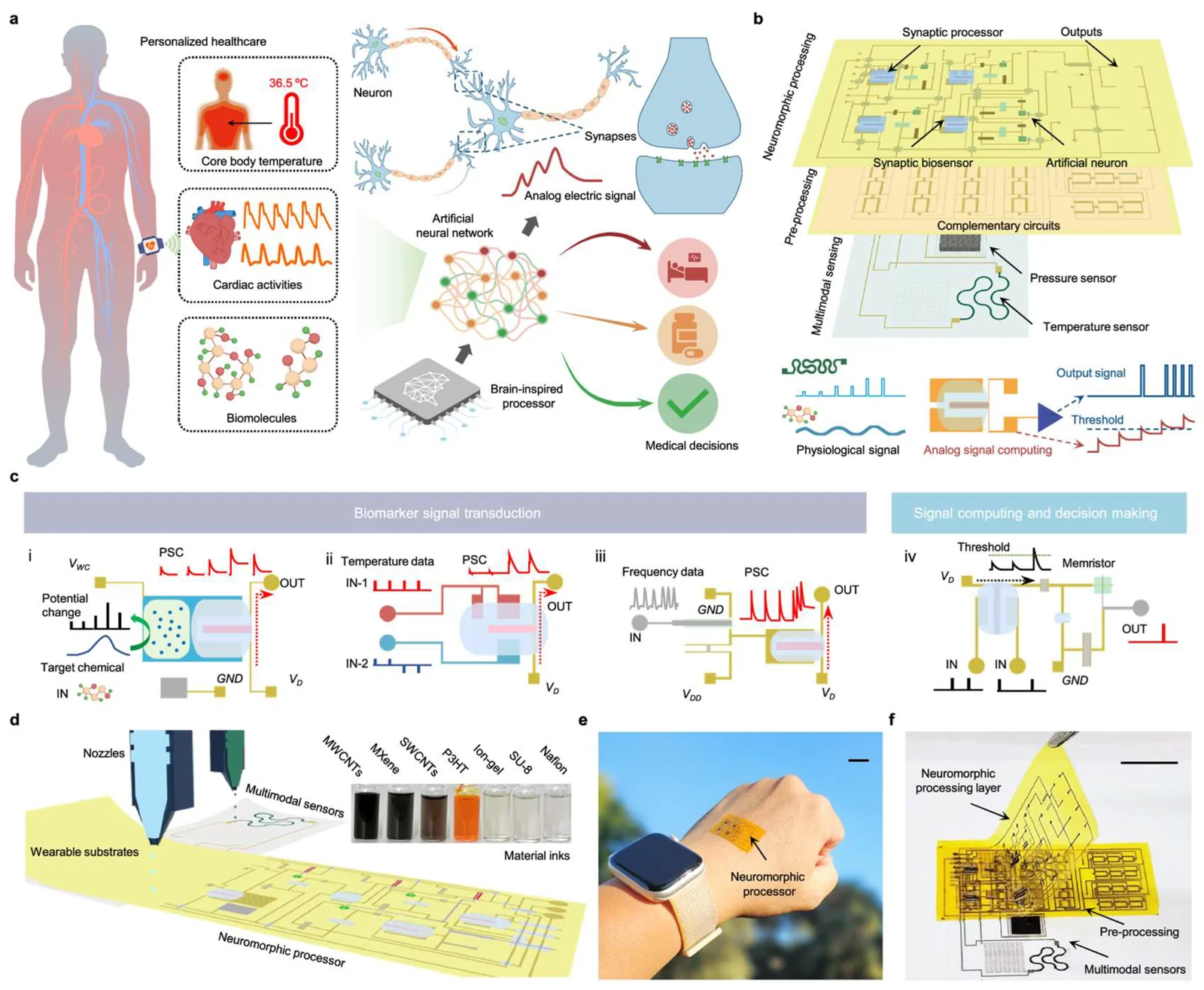
All-printed, chipless wearable sensor-processor-integrated neuromorphic system for multimodal physicochemical health monitoring. Reproduced from Choi et al., *Nature Communications*, 2025, under the Creative Commons Attribution 4.0 International License (https://creativecommons.org/licenses/by/4.0/). Displayed with resizing only; no scientific content was modified [101]. (**a**) Schematic illustrations of the wearable CSPINS, designed to collect, process, and analyze multimodal physicochemical data for real-time medical decision-making. (**b**) Layered architecture of CSPINS, consisting of a multimodal physical sensing layer, a complementary circuit amplifier layer, and a synaptic sensing and neuromorphic processing layer. (**c**) Data flow within CSPINS, detailing the stages of signal transduction and processing of synaptic biochemical sensors (**i**), core body temperature sensor (**ii**), heart rate sensor (**iii**), as well as computing and decision-making operations (**iv**). IN, input; OUT, output; GND, ground; VDD, positive supply voltage; VD, drain voltage; VWC, weight-control voltage. (**d**) Scalable fabrication of the wearable neuromorphic device using inkjet printing with a variety of organic and inorganic nanomaterial inks. PI, polyimide; PET, polyethylene terephthalate; MWCNTs, multi-walled carbon nanotubes; SWCNTs, single-walled nanotubes; P3HT, poly(3-hexylthiophene). (**e**) Optical image of the wearable neuromorphic processor attached on skin. Scale bar, 1 cm. (**f**) Optical image of the fully assembled CSPINS, featuring integrated physicochemical health sensors and processing units. Scale bar, 1 cm.

As the processing of data at wearable biochemical systems’ edge increases, so do its benefits: power, privacy, real-time failure detection, and low-latency alerts being main examples. Flexible systems benefit greatly. System designs that utilize neuromorphic and in-sensor computing reduce the need to transfer high-rate raw data. As flexible systems utilize edge intelligence (i.e., data processing), accepting validation becomes more complex. Preprocessing embedded directly in hardware makes it difficult to identify the source of the error within the object. In contrast, locally updated models make it more difficult to maintain version control or to provide regulatory documentation. Approaches to preserving privacy (e.g., federated learning, differential privacy [121], and secure aggregation) allow population modeling without necessitating centralized storage of raw biochemical data [122,123]. However, the need for representative data, transparent consent, and post-marketing monitoring remains. Biochemical trajectories provide indirect estimates of medication compliance, alcohol exposure, stress rhythms, reproductive status, and disease behavior. The default architecture should collect only the minimum context necessary for the intended purpose and should justify each data stream. For example, a hydration tool may not require geolocation, while a pharmacokinetic tool may require dose timing but not continuous location tracking. Figure 10 presents the direction of all emerging printed, chipless neuromorphic systems. This class of platforms has the potential to directly process biochemical and biophysical data while being worn on the body. The major translational lesson here is edge intelligence, which makes wearables more autonomous and secure. Still, it requires implementing more extensive documentation for model versions, preprocessing, failure detection, and explainability. An intelligent patch should be auditable post-deployment [23,28].

### 8.3. Dataset Structure, Model Cards, and Leakage-Resistant Evaluation

AI-ready biosensing should be implemented as a data product, not as an afterthought attached to an app. A reusable dataset for continuous biochemical monitoring should contain raw sensor streams, calibrated concentrations, metadata, signal-quality flags, reference measurements, user annotations, protocol events, and device health logs. The raw streams must be retained because future drift models, artifact detectors, and calibration methods may need information that a current preprocessing pipeline discards. At the same time, calibrated estimates should be versioned because a change in the calibration equation can alter every downstream feature. A data record should therefore state not only the analyte value but also the firmware version, preprocessing version, calibration model, sensor lot, and quality-control state [23,24]. Rather than just showing uncertainty, it should be kept and then evaluated. Sources of uncertainty exist in analyte measurement (due to calibration errors, noise, matrix effects, temperature compensation errors, and comparator errors). For physiological variables, uncertainty can arise from time window selection, lag adjustments, and baseline estimation. When producing decision-support outputs, uncertainty also encompasses trade-offs between risks and benefits in diagnosis/classification. Models will be rewarded for refraining from recommending any sampling when they lack confidence in the reliability of that sample and penalized for providing a confident but incorrect recommendation. Prediction interval coverage, calibration curves, abstention utility, and decision-curve analysis can operationalize uncertainty. Missing values should be treated as data. In biochemical sensing via wearables, often there will be missing values due to sweat production by the body being too low (i.e., not enough sweat), a blockage in the channel, failure of the adhesive to maintain contact, the user having removed the wearable, a loss of wireless communication capability, or a failure in the signal-quality check. Each of these situations is significant in terms of the type of missing information. Low sweat production may reflect a hydration risk or a thermal risk; adhesive failure may reflect ergonomic design limitations; and algorithmic abstention may represent a form of safety feature. If an overall dataset codes for all missing values as empty cells, then this is lost. An AI-ready dataset should maintain a reason for each missing value (where possible) and assess model performance during and after periods of missing values [116,117].

Leakage prevention is one of the most common weaknesses in wearable machine learning. Time-series datasets contain repeated measurements from the same person, device, protocol, and day. Randomly splitting time windows can place highly similar segments into both training and testing sets, inflating performance [117]. The correct split depends on the intended use. If the system will be deployed to new users, leave-subject-out or external cohort validation is essential. If the system will be used with a new sensor lot, lot-stratified validation should be included. If the system will operate in new environments, external field testing matters more than additional cross-validation within a laboratory protocol. Performance should be reported across these axes rather than as one aggregate number [118,124]. For wearable outputs framed as individual-level diagnostic, prognostic, or monitoring predictions, evaluation should additionally distinguish model-development quality from model-evaluation risk of bias and applicability. In line with PROBAST + AI, this assessment should address participants and data sources, predictors, outcomes, analysis, missing-data handling, calibration, discrimination, clinical utility, fairness, data leakage, and external validation [125]. Model cards are useful for wearable biosensor algorithms because they require developers to specify the intended use, training population, input streams, excluded populations, validation design, known failure modes, and update policy. Models that utilize sodium in sweat, sweat rates, temperature, and motion to predict the risk of heat stress must specify whether they were developed using indoor cycling, outdoor labor, military exercises, or clinical cases of heat illness. Drug-monitoring models must specify dosage regimens, comparator assay methods, and the pharmacokinetic assumptions upon which the model is based. Models used to assess stress must specify whether their outputs indicate exploratory trends in wellness or validated clinical endocrine status. If this type of documentation is not provided, the model could progress rapidly from research to product claims without sufficient supporting evidence [23,24].

### 8.4. Foundation Models, Domain Adaptation, and Mechanistic Constraints

An important point will be to establish an explicit definition of when A.I. is no longer needed. For some safety-related tasks, simple threshold- or rule-based quality checks or transparent Bayesian filters are more suitable than complex models. Complex models are warranted only if they can improve generalization, uncertainty, personalization, or decision support; however, the inclusion of multimodal data alone does not justify using a complex model. A high-impact review of the literature will help hold the community accountable to the following critical questions: specifically, what ambiguity does the model resolve, what evidence exists to support that the model resolves that ambiguity, and what are the outcomes in instances when the model would be incorrect [126,127]?

This level of accountability will help increase the overall credibility and likelihood of publication for A.I.-enabled biosensors [128]. It may also lead to additional hybrid mechanistic–machine learning models. Mechanistic modules may be used to describe variables such as diffusion, flow, temperature dependence, binding kinetics, or pharmacokinetics. In contrast, the learning module may be used to account for residual variation and across-individual differences. This hybrid structure is more interpretable than a fully black-box model alone and can also reduce the amount of labeled data required to make accurate predictions [129,130]. For instance, a drug-monitoring wearable device may employ a pharmacokinetic model to develop a framework for determining subject-specific metrics related to drug absorption or for identifying signs of bias in the results due to sensor malfunction; similarly, a hydration model may combine physiological estimates of sweat sodium concentration with empirically determined relationships between sweat sodium concentration and key environmental variables (e.g., temperature and humidity) or key physical exertion variables (e.g., intensity and frequency); and a wound healing model may integrate observations from local biochemical trends, clinical imaging, and dressing information.

It will require extensive domain adaptation because each of these devices will likely have multiple lots and may be used across different body areas or by users in different climates, with different firmware versions, etc. A machine learning model developed for a particular type of sweat patch or adhesive may not generalize to an entirely different type of sweat patch, a microneedle device, another tissue bed, or a spectroscopic assay’s substrate batch; transferring it to another might result in significant prediction discrepancies. Manuscripts must document these types of shifts in their methodology and include the results of calibration-transfer experiments (i.e., how well does the model generalize to the new condition?), lot-to-lot validation (i.e., was the model generalizable to the new lot?), body-site transfer (i.e., how well did the model generalize to new body sites?), external cohort verification (i.e., how well did the model generalize to an independent cohort?), and field usage as standard evaluation metrics for newly developed models [122,123].

When authors employ the adaptation process, the machine learning algorithm must specify the type of data required from the new environment and whether the model demonstrates safe predictive ability before adaptation is completed. Studies involving large sequence models and multimodal foundation models will undoubtedly introduce a unique body of literature on wearable biochemical sensing applications; however, there should also be physical and biological limits on their use for measurement. If data from biological data streams are generated by a strong relationship (or correlation) between biological processes and corresponding physical processes (e.g., molecular transport, sampling, recognition, electricity, firmware, and behavior), then the modeling of such would lead to significant model difficulty and a risk of error; in contrast, a model that does not consider these variables may create incorrect predictive relationships with other variables of interest (e.g., predicting lactate from motion data) and, as a result, develop associations that do not correspond to the physiological state of the individual (e.g., predicting sweat rate from amplitudes of the signal, at times without consideration of evaporation). This requires that strong models include mechanistic priors, explicit quality channels, and compartment-aware time-shift structures [126,127].

A representative end-to-end implementation of the proposed framework is the iCares smart bandage reported by Wang et al. [11]. The clinically defined task was to characterize the chronic-wound status and healing-time category using wound-exudate chemistry together with patient context. At the biofluid layer, a pump-free microfluidic architecture incorporating a Janus membrane, wedge channels, and graded micropillars collected, transported, and refreshed low-volume wound exudate. At the recognition and hardware layers, a flexible sensor array measured NO, H_2_O_2_, O_2_, pH, and temperature and transmitted the signals wirelessly. During 2 h measurements in 20 patients with diabetic or venous chronic wounds, the sensor data were calibrated, summarized, standardized, and combined with contextual variables, including diabetes, dialysis status, peripheral artery disease, age, and wound area. A linear support vector classifier yielded reported internal accuracies of 88.75% for wound classification and 94.0% for healing-time-category classification, while SHAP analysis identified the biochemical and clinical variables contributing to the predictions. The wearable O_2_ channel also correlated with a clinical oxygen-imaging comparator (r = 0.943) [11].

When mapped to the readiness levels defined in Section What Makes a Wearable Biochemical Sensor AI-Ready?, the study provides evidence of Technical Readiness through integrated sampling, sensing, and telemetry; Contextual Readiness through matrix-specific fluid handling, temperature compensation, and patient-level covariates; Statistical Readiness through multimodal feature modeling, repeated internal resampling, and SHAP-based explainability; and initial Clinical Readiness through testing in the intended patient population. Equally importantly, the framework identifies the remaining evidence gaps. The model was developed from a single cohort of 20 patients using an 80:20 random train–test split repeated 250 times rather than an independent external cohort; feature extraction was performed asynchronously using mean values; monitoring lasted only 2 h; and prospective clinical utility was not evaluated. Accordingly, the reported output should be interpreted as a research-stage classifier rather than a validated clinical decision-support system. This case demonstrates how the proposed framework can trace an AI-enabled wearable from biofluid access to a decision-relevant output while preserving appropriate claim boundaries [11].

## 9. Clinical Translation and Regulatory-Grade Evidence

Many wearable biosensor prototypes fail to achieve clinical translation. Even if the chemistry is sophisticated, the flexible substrate has appealing properties, and the app’s visual aspect is compelling, the evidence chain may still be incomplete. The process of clinical translation will require analytical validity, valid body-fluid testing, robustness in on-body tests, valid clinical evidence, clinical utility, and governance to cover the device’s lifecycle [25,28]. Each gate will measure something different; for example, does the sensor measure the intended target under controlled conditions and in the presence of high interference? Does the liquid collected for analysis provide sufficient evidence of the physiology being claimed for that sensor? Does the unit operate on real skin with the natural use patterns of a typical user? Does the output from the device relate to a clinically meaningful outcome? Will using the sensor’s output help with decision-making or lead to a better outcome? Will the system remain safe throughout the product’s lifecycle, including after its release and subsequent updates? International frameworks that regulate medical devices will provide high-quality guidance on the progression of these devices. Clinical evaluation of software as a medical device prioritizes establishing a valid clinical association between the outcome and the sample type, analytical validation of the measurement type, and clinical validation of the end-user output (i.e., following this cycle) [25]. The concepts associated with good machine learning practice apply equally to AI-enabled medical devices, in terms of a multidisciplinary approach to development, representative datasets in research, independent testing of results, clinically relevant results, clarification to the user of the differences between human and AI decision-making [131], monitoring of both AI and humans, and management of the lifecycle of the AI device. Wearable biochemical devices typically include hardware, wet chemistry, and software; thus, the regulatory process must begin shortly after the prototype stage rather than be added afterward [29,132]. For example, biofluid validity is separate from analytical validity because this is often a significant source of overstatement. Although a sensor can accurately detect lactate in sweat, there is no evidence that a diagnostic blood lactate measurement correlates with the user’s perspiration. Robustness in on-body testing is separate from clinical validity because, although the device may provide the same result as a comparator device in an experimental setting, it may not do so in routine daily use. Similarly, clinical utility is distinct from clinical validity because an accurate measurement can provide no improvement in decision-making or outcomes. Another gate in clinical translation is governance, which covers the lifecycle of use, as AI-enabled wearable devices may drift following release due to sensor aging, changes in manufacturing processes, shifts in user behavior, and periodic updates and retraining of the predictive model. Table 11 summarizes how analytical, body fluid, body robustness, clinical validity, and lifecycle evidence relate to the inherent failures that arise during clinical translation [119,133].

**Figure 11:**
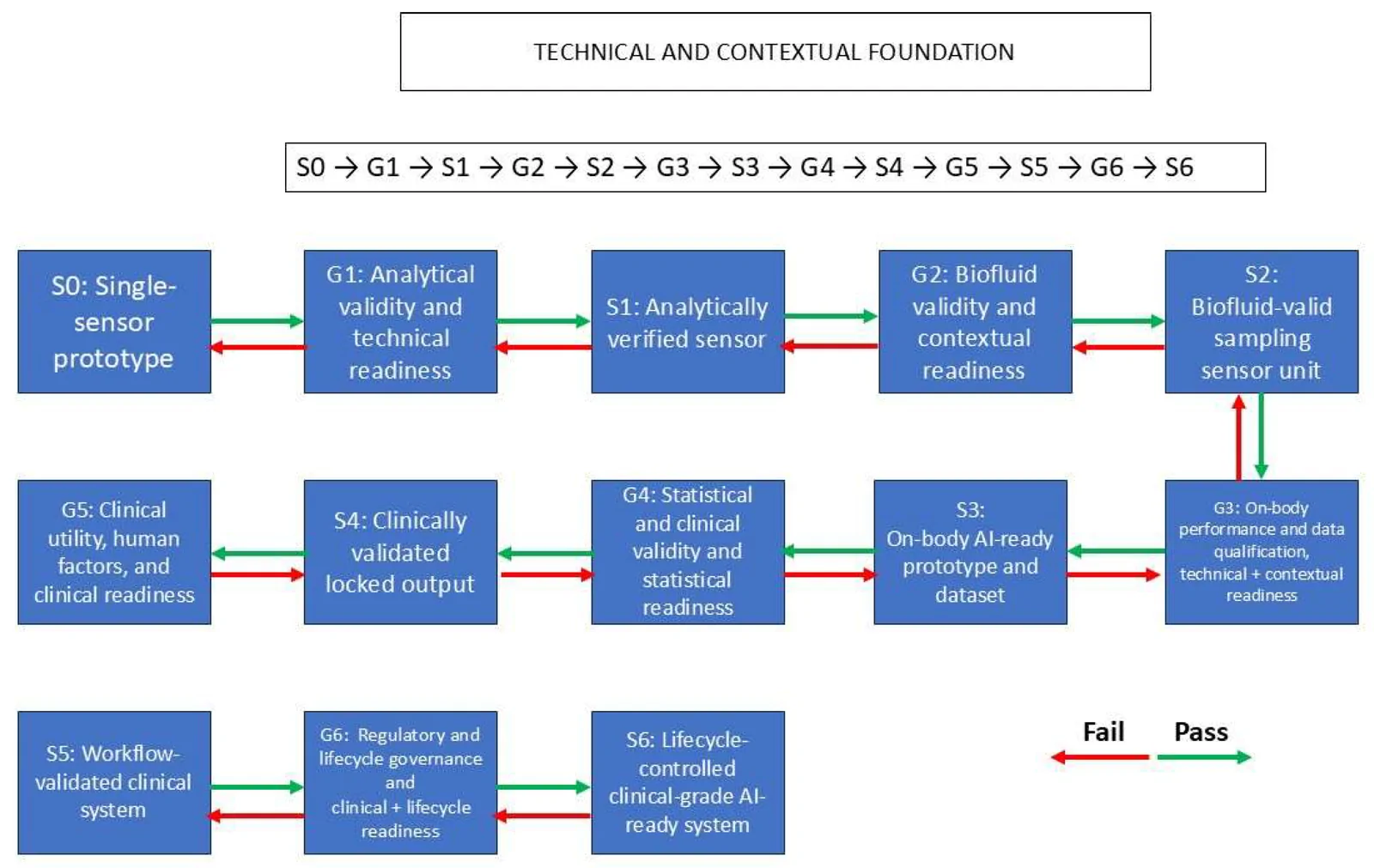
Non-compensatory stage-gate pathway from a single-sensor prototype to a lifecycle-controlled, clinical-grade, AI-ready wearable biochemical system. Seven development stages are connected by six gates: analytical validity; biofluid validity; on-body performance and data qualification; statistical and clinical validation of a locked output; clinical utility and human-factors validation; and regulatory/lifecycle governance. PASS indicates advancement only when all critical, prospectively specified, context-of-use-specific criteria are satisfied. FAIL indicates a need for redesign, additional evidence generation, and repetition of the affected gate, while STOP/REFRAME applies when the biomarker–biofluid–claim relationship is unsupported or residual risk is unacceptable. Material changes and post-deployment drift reopen the earliest affected gate. “AI-ready” at Stage S3 denotes a versioned, provenance-rich, time-synchronized dataset and pipeline containing reference labels and quality/context metadata; it does not indicate that an AI model has been clinically validated. “Clinical-grade” denotes completion of a claim-specific evidence and governance package and does not itself indicate regulatory authorization [18,25,28,132,134].

Table 10 defines the evidence domains required for translation. In contrast, Figure 11 operationalizes them as a non-compensatory stage-gate pathway from a single-sensor prototype to a lifecycle-controlled clinical-grade AI-ready system. Before Gate 1, the development team should prospectively define the analyte–biofluid pairing, target population, use environment, intended user, required wear duration, output claim, comparator, action pathway, risk level, and gate-specific acceptance criteria. A PASS decision is made only when all critical criteria applicable to that context of use have been satisfied and documented under a locked protocol. Because acceptable analytical and clinical performance depends on the intended concentration range, reference-method uncertainty, clinical consequences of error, target population, and device risk, the pathway does not impose universal numerical cut-offs. A FAIL returns the system to the earliest affected development stage. In contrast, an unsupported biomarker–biofluid–claim relationship or unacceptable residual risk requires the intended use to be reframed or development to stop. Material changes and post-deployment drift similarly reopen the affected gates [18,25,28,132,134].

Figure 11 operationalizes development as an author-developed, sequential, non-compensatory stage-gate pathway. Before testing begins, the analyte–biofluid pairing, target population, use environment, intended user, required wear duration, output claim, comparator, action pathway, risk level, and gate-specific acceptance criteria must be prospectively justified and locked. At G1, PASS requires the sensor to cover the intended decision range in the relevant matrix and to meet the predefined requirements for bias, precision, selectivity, interference, response time, drift, stability, and device- and lot-level reproducibility; FAIL requires redesign of the recognition chemistry, transducer, electronics, or calibration, followed by repetition of G1. At G2, PASS requires characterization of recovery, matrix effects, sampling volume and dynamics, lag, carry-over, contamination, body-site effects, and sampling chronology in authentic target biofluid, together with a time-aligned reference measurement or appropriate local endpoint supporting the intended physiological claim; FAIL requires modification of the sampling system, microfluidics, matrix correction, or comparator timing, while an unsupported biomarker–biofluid relationship requires STOP/REFRAME of the intended claim. At G3, PASS requires the integrated device to meet prospectively specified wear-duration, usable-data-yield, failure-rate, safety, comfort, and usability criteria in representative users and conditions and to generate synchronized, versioned signals, contextual metadata, quality-control flags, and reference labels; FAIL requires redesign of the device, fluidics, firmware, quality-control channels, or user interface, whereas unacceptable residual safety risk requires development to stop. At G4, PASS requires a locked preprocessing, model, and decision-threshold pipeline to meet prespecified performance, calibration, uncertainty, failure-detection, out-of-distribution, and subgroup-safety criteria on appropriately independent data without participant, site, device-lot, or temporal leakage; FAIL requires model revision or additional data collection followed by evaluation on a new, untouched test set. At G5, PASS requires prospective evidence, in the intended population and workflow, that the system achieves the predefined clinical or operational endpoint with an acceptable action threshold, escalation pathway, alert burden, human–AI interaction, and critical-use-error profile; FAIL requires revision of the claim, threshold, interface, or workflow and repetition of G5. At G6, PASS requires verified quality and risk management, production and calibration traceability, cybersecurity, privacy, auditability, version control, post-deployment monitoring, update or retraining, corrective action, and rollback procedures; FAIL requires deployment to be withheld until the missing controls are completed. At every gate, failure of one claim-critical criterion prevents advancement, and later-stage performance cannot compensate for an upstream failure. Table 2 provides cross-platform default minima for SNR, normalized drift, data sufficiency, and lot-to-lot degradation. Classification and agreement thresholds remain context-specific but are fully operational: F1_min_^COU^ and the Bland–Altman allowable-difference band A(c) must be justified and locked before testing, and the specified confidence-bound and cross-lot degradation criteria must subsequently be satisfied. Material changes or post-deployment drift reopen the earliest potentially affected gate [18,25,28,132,134].

### 9.1. Comparator Alignment and Reference Sampling

False confidence arises from selecting inappropriate comparators. A sweat sensor shows low correlation with blood due to the compartments and time constants the fluids are subject to. Likewise, an athletic exertion trial demonstrates a strong correlation between participants’ sweat and blood readings, but the correlation does hold for sweat and blood measurements in their daily life following the exertion [119]. When a biological claim related to systemic equivalence is made, the biological sample comparator must match in timing and lag to the blood or plasma results from the biological claim, whereas, if making a biological claim of a concentration, there may be a more appropriate comparator type available based on the tissue of interest or clinical outcome. If making a biological claim based on trends, repeating measures and aligning events would be more informative than using isolated paired samples for comparator-related data accuracy [120].

Inherent uncertainty exists in all comparators. The laboratory assays used to establish the laboratory sample comparator are subject to random error, pre-analytic variability, and the timing of laboratory samples. Patients’ self-reported meals, symptoms, or medication intake may also provide inaccurate comparators for biological claims made. The accuracy of wearable physiological streams (e.g., heart rate) can be compromised during physical activity. Failure to account for uncertainty in all derived labels leads to treating all labels as perfect, resulting in overfitting and underrepresenting the magnitude of uncertainty. When possible, propagate label-based uncertainties through mathematical models and, where needed, validate them prospectively in the intended workflow [116,119].

### 9.2. Human Factors, Equity, and Real-World Deployment

Human factors should be treated as determinants of effective system performance rather than as late-stage usability considerations. Evaluation should begin with intended users and workflows. It should include donning and removal, adhesive and mechanical stability, wear comfort, skin irritation, local sweating, hair density, skin texture, anatomy, motion, wear duration, climate, and foreseeable misuse [134]. Interfaces should distinguish a measured analyte signal from a corrected estimate or model-derived output, communicate uncertainty and invalid-data states, and provide actionable alerts without imposing excessive cognitive load or alarm fatigue. For clinicians, summarized outputs should integrate with the intended workflow rather than transfer the burden of interpreting continuous unfiltered time-series data.

Skin pigmentation requires a mechanism-specific assessment. When light traverses or is reflected from tissue, as in photoplethysmographic, spectroscopic, or other transcutaneous optical measurements, epidermal melanin and other chromophores can alter wavelength-dependent absorption, scattering, signal amplitude, and signal quality. However, the magnitude and direction of the effect are device-, wavelength-, geometry-, site-, and context-specific; it should not be assumed that every optical wearable performs less accurately on darker skin. Evidence from wearable photoplethysmography further shows that pigmentation-related inequity may appear as missing values, outliers, or rejected windows even when differences in mean error among retained measurements are small. The mechanism is different for external colorimetric or fluorescent biochemical patches: pigmentation is a direct interferent only when the underlying or adjacent skin contributes to the excitation–detection path, image background, or region-of-interest segmentation. Even with an opaque backing, the ambient-light spectrum, shadow, viewing angle and distance, flash, automatic exposure and white balance, camera spectral response, and contrast with adjacent skin may affect smartphone-derived color estimates [135,136,137,138].

Race and ethnicity should therefore be recorded as social descriptors but should not be used as proxies for pigmentation. Intended-use studies involving an optical pathway should independently quantify pigmentation at the actual sensor site using a calibrated CIE L*a*b*/Individual Typology Angle or melanin-index measurement; Fitzpatrick phototype alone is not an adequate pigmentation measure. Pigmentation should be analyzed continuously and in prospectively defined bands. Error or agreement, calibration, clinically relevant sensitivity and specificity, usable-data yield, missingness, quality-control rejection, and algorithmic-abstention rates should be reported with participant-clustered 95% confidence intervals. Interactions with motion, perfusion, illumination, camera model, anatomical site, device placement, and wear conditions should also be evaluated. An equitable-performance claim requires a powered equivalence or non-inferiority analysis against a prospectively justified maximum allowable subgroup difference. Reference-color markers, controlled or measured illumination, optical shielding, opaque backings, device-specific camera calibration, and image-quality rejection are risk controls, but they do not replace subgroup validation [135,136,137,138].

Equity in sweat sensing similarly requires sweat availability to be treated as a measured physiological input rather than inferred from demographic labels. Sweat secretion depends on the evaporative requirement for heat balance, metabolic heat production, body size and morphology, anatomical site, exercise or occupational workload, ambient temperature and humidity, clothing, acclimation, hydration, fitness, stimulation method, health status, and medication. Age- and sex-associated differences have been observed, but their magnitude varies with heat strain, body region, and protocol; race or ethnicity and sex should not be converted into biological calibration coefficients without a validated analyte-specific mechanism. Low secretion can delay microfluidic filling or provide an insufficient sample, whereas high secretion can alter transit time, ductal reabsorption, analyte concentration, and sensor saturation. Heat acclimation can increase local sweat rate while decreasing several electrolyte and metabolite concentrations, demonstrating that concentration changes cannot be interpreted as simple dilution [40,139,140,141].

Validation should therefore record the local sweat rate, collection area and body site, stimulation method, workload, temperature, humidity, acclimation or season, hydration status, collection duration, and obtained volume. Analyte concentration C, local areal sweat rate q, and, when physiologically justified, analyte flux J = Cq should be reported separately; automatic normalization of every analyte by sweat rate is inappropriate. A device-specific minimum valid secretion rate can be prospectively derived as q_min = V_fill/(A_collect × t_max), where V_fill is the volume required to reach the sensing region, A_collect is the collection area, and t_max is the maximum acceptable sampling delay. Below this threshold, the system should return “insufficient sample” or abstain rather than convert absent or delayed sweat into a low-analyte result. Time to first valid result, usable-window yield, insufficient-sample rate, analytical error, and abstention should be reported for prespecified age, sex or gender, body-morphology, disease, medication, and use-environment groups.

Algorithmic equity cannot be inferred from pooled accuracy or from simply omitting protected attributes from the model. Bias may enter through non-representative recruitment, reference-standard or label error, sensor-dependent missingness, preprocessing and quality filtering, class imbalance, threshold selection, participant leakage, and shifts across sites, phone or camera models, device versions, manufacturing lots, environments, or time. All repeated windows from one participant must remain within the same data partition, and claim-level testing should be participant-disjoint and include independent site-, device-, and lot-level challenges whenever these sources of variation fall within the intended use [24,28,29,36,37].

Equity-relevant strata and clinically plausible intersections should be specified before analysis. Depending on the measurement mechanism and intended use, these may include age, sex, gender, objectively measured pigmentation, race or ethnicity, body morphology, comorbidities, medication, socioeconomic and geographical context, device or site, and environmental conditions. Subgroup estimates and confidence intervals for error or agreement, calibration, sensitivity, specificity, predictive values, false-alert burden, usable-data yield, missingness, quality-control failure, and abstention should accompany pooled results. Complete-case accuracy can conceal inequity when one group is less likely to receive a valid result. Insufficient subgroup size should be reported as uncertainty and residual risk rather than interpreted as evidence of equivalence. No single statistical-parity measure is appropriate for every clinical claim; the relevant metric, maximum allowable disparity, and safety-critical subgroup floors must be justified prospectively from the intended use and consequences of error. Subgroup-specific recalibration or thresholds must be mechanistically and clinically justified, frozen before independent testing, and evaluated for both benefit and harm rather than being used to conceal an upstream sensing failure [24,28,29,36,37].

Finally, validation in a well-connected tertiary center does not establish equitable deployability in a resource-constrained setting. Intended-use evaluation should reproduce locally available phones and operating systems, computing capacity, intermittent electricity and connectivity, data costs, heat, humidity, dust, storage conditions, staffing, workflow, referral capacity, and access to a fit-for-purpose comparator. Safety-critical functions should not depend on uninterrupted connectivity unless that dependency is explicit and a safe outage pathway has been demonstrated. Where compatible with the claim, systems should support offline-first acquisition and quality control, on-device inference, encrypted local buffering, visible synchronization and software-version status, and deterministic recovery from interrupted or duplicate transfers [142,143].

Deployment planning should additionally cover low-power operation and locally feasible charging; interfaces adapted to local languages, literacy, accessibility, and shared-device use; data minimization and locally appropriate consent; shelf-stable consumables and quality-control materials; interoperable data export; training and technical support; and access to calibration, spare parts, repair, and replacement. Affordability should be evaluated through total cost of ownership—including the reader or phone, patches, reagents, connectivity, charging, calibration, training, maintenance, clinician time, replacement, and disposal—rather than purchase price alone. Performance, usable-data yield, and human-factor outcomes should be reverified following language adaptation, offline–synchronization cycles, and environmental-stress testing with locally recruited users and health workers. Reusable and disposable components should be distinguished, with defined battery, chemical, and electronic-waste pathways. A low initial price is not equitable if recurrent consumables, downtime, privacy risks, or waste-management burdens are transferred to under-resourced users.

Equity is therefore a non-compensatory evidence requirement, not a demand for identical physiological distributions among demographic groups. Where the author-proposed 90% usable-data default in Table 2 is applied, the same locked criterion should be examined within every prespecified safety-critical subgroup, together with the claim-specific accuracy or agreement limit. Aggregate performance cannot compensate for a subgroup that cannot reliably obtain a valid and safe output. Within Figure 11, pigmentation and sweat-generation effects are addressed at G2–G3; subgroup and site/device generalization at G4–G5; and access, maintenance, monitoring, and deployment drift at G6.

### 9.3. Regulatory-Grade Lifecycle Planning for AI-Enabled Biochemical Wearables

Evidence of regulatory grade also necessitates evaluation of human factors, as a user may misinterpret a signal that technically meets the maximum specifications, making it unsafe to use. User interfaces must distinguish between the measured concentration of an analyte, a corrected estimate of its concentration, a physiological characteristic, and the output from decision support. Interfaces must indicate when data quality is compromised and avoid unsupported diagnostic language. For systems used by clinicians, outputs must integrate into their workflow while reducing cognitive load compared to presenting continuous raw-data traces. For systems used by patients or other users, alerts must be actionable; sufficient population data must be available to prevent alert fatigue, and there must be explicit communication about whether a medical evaluation is necessary at the point of receiving the alert [134]. Cybersecurity and privacy are aspects of device safety. Continuous biochemical signal data can also provide information about medication use, circadian stress patterns, alcohol exposure, reproductive status, or a patient’s illness. Examples of attack surfaces include those created by wireless communication, cloud storage, and mobile application interfaces. A responsible system minimizes data collection, encrypts data transferred wirelessly, authenticates devices, and, when practical, separates previously collected identifiers from raw data streams and defines policies for data retention [144]. Clinical-grade systems also maintain an up-to-date record of all devices and models, including both soft- and hard-product components, as well as calibration history. If a user receives a recommendation that has harmful consequences, the system must be capable of reconstructing which data and models produced that recommendation so it can be addressed [132,144].

Planned lifecycle activities for a system should begin before human testing. This lifecycle program should also operationalize the FUTURE-AI principles of fairness, universality, traceability, usability, robustness, and explainability, with continuous and transparent risk assessment extending from design and validation through deployment, monitoring, updating, and decommissioning [145]. Authors of research publications must specify the sections of the system that are fixed and cannot be altered before testing begins (e.g., electrode design, enzyme lot number, microfluidics design, firmware version, calibration model, algorithm for determining signal quality, model for determining clinical risk, and user interface). Changes to individual components/systems may affect system performance in various ways (for example, a system’s sensitivity could change with the introduction of a new lot number for an enzyme, and the methodology used to glue electrodes may affect sweat capture). If updated firmware changes how data is processed before results are returned to an end user, a system’s performance could be altered. The governance of model changes in AI-enabled systems will require special consideration and approval [28,132], as data indicate that average model performance may improve. In contrast, performance in a subgroup of patients may decrease. Therefore, systems must be continually monitored after deployment to ensure sufficient data to evaluate overall system performance, subgroup performance, lot-to-lot device performance, the occurrence of missing data and false alerts, and the frequency of patient complaints and adverse events. When a wearable biochemical biosensor produces results that assist with a clinical decision, medication dosing, an occupational safety action, or escalating wound-repair treatment, it is considered higher risk for use than if it did not. Therefore, validation must include reviewing both the biochemical sensor and the software layer that interprets its data. The components of the evidence architecture must support the intended claim associated with the results produced. If the result produced is intended to provide a prediction of wellness, that limitation of the evidence architecture must be clearly stated in the manuscript. If the output of the evidence architecture will assist with clinical decision-making, the manuscript must define the target population, the circumstances surrounding the delivery of the output, the action threshold for responding to the output, the end-user role, and the risk controls in place to manage the risk of delivering the output. Verification of intended use is a critical component of manufacturing because the same system can be classified as a wellness product or a medical device, depending on the regulatory approval path used [25,132]. Consistent with this claim-based approach, FDA’s 2026 final general-wellness policy uses a non-invasive athletic wearable reporting validated electrolyte, lactate, and hemoglobin values as an illustrative potentially low-risk general-wellness use when its claims remain limited to exercise or fitness. Outputs intended for disease screening, diagnosis, monitoring, clinical alerts, or treatment decisions fall outside that policy [146]. This US-specific example does not itself establish the safety, effectiveness, or regulatory status of an individual device.

## 10. Failure Modes and Built-In Self-Diagnostics

Future biochemical wearable sensors must be built to self-diagnose. Similarly to a laboratory instrument, a device will undergo routine calibration checks, verification of blank samples, reagent log entries, and maintenance checks. A wearable, when attached to a body, is perceived to work. This inference is unsafe because wearables are subject to motion, sweat, drying out, compression, contact with skin oils, temperature fluctuations, wireless connectivity failures, and user touch. During the design phase of a device, all potential failure modes should be identified and linked to diagnostic signals for feedback [16,134]. If a device cannot determine whether a true analyte excursion is due to improper sampling, then clinical data should not suggest that an excursion occurred. Sampling failures can include no sweat collected, a mix of aged sweat, blockage within a channel, air bubbles, leakage, evaporation, and contamination. Microfluidic fill sensors, impedance measurements, optical markers, flow estimates, and timing patterns can help detect sampling failures. When working with systems that use interstitial fluids, data quality may be compromised by factors such as insertion failure, incomplete insertion, local bleeding, tissue response, or microneedle fracture. Using electrical impedance, feedback from the insertion force, baseline signal stabilization, and redundant electrodes may all help identify these deficiencies [49,147]. User-related failures include poor placement of the device, failure to replace or reapply the device, showering, use of cosmetics, and pressure from clothing. They should be viewed as design failures rather than simply user failures. Some of the failures due to chemical-interface issues include enzyme degradation, mediator leaching, membrane delamination, receptor saturation, nonspecific adsorption, pH shift, and overload/interference from other substances. Failures due to mechanical or electrical issues will include cracking of the device, delamination of the device, an increase in resistance (strain-induced), ingress of water, depletion of the battery, failure of wireless communication, and saturation of the Analog Front End (AFE). The device’s firmware should record all resets, battery voltage, memory state, packet losses, and sensor saturation [144]. All of the above failures will provide guidelines for estimating uncertainty. During times of uncertainty, the system will, by default, remove unreliable data. Rather than removing unreliable data from the device, the device will label the period as high confidence, low confidence, sampling failure, loss of contact, calibration drift, or insufficient context. A mapping of the diagnostic signals related to the reasoning behind the major classes of failure with wearable devices is presented in Table 12.

## 11. Use-Case Blueprints for High-Value Translation

Use-case blueprints, rather than analyte lists, will improve efficiency in advancing the field. The heat-stress blueprint starts with the action to be taken based on a decision [40,86]. These decisions include whether to continue working, rest, replace fluids, cool down, and consider other variables such as skin temperature, ambient temperature, humidity, heart rate, relative activity level/intensity, and symptomatology. For example, the output will not simply be the sodium concentration. Still, it will also include the risk trajectory (and confidence) of how much risk existed and its severity, and an explanation for why the risk occurred (e.g., fluid-volume loss, thermal stress, poor sampling technique, or an unreliable patch). The design of the athletic recovery blueprint will also be different from the previous two examples. Lactate, glucose, and ketones may be good indicators of recovery; however, the biological question is whether the individual has accumulated metabolic stress and has effectively recovered or is showing initial signs of overreaching. Answering this question will require evaluating the composition of heart-rate zones, perceived exertion, sleep duration, individual training plans, environmental heating, and personal baseline. A small increase in lactate during low-level activity may provide greater predictive value than a large increase during planned high-level activity. The model must learn, in the context of the individual and the training environment, what is required for the individual to locate transition points [67,86].

The wound monitor blueprint is to be considered local rather than systemic. The relevant channels include PH, temperature, uric acid, lactate, oxygen, moisture, proteolytic activity, and cytokine/bacterial signatures. The device must minimize disruption to the wound bed [148,149], manage exudate, maintain sterility, and produce a clinician-ready summary of findings [150,151]. Alternatively, laboratory reference samples do not include venous blood but include photographic records of the wound, microbiological data, clinical scores, timeframes for wound healing, and treatment decisions [152,153]. A successful wound-monitoring wearable device will reduce the frequency of unnecessary wound dressing changes while triggering earlier interventions when biochemical and thermal variations indicate risk of harm [154,155,156,157]. A therapeutic-drug-monitoring blueprint should include pharmacokinetic actionability. The challenge to be overcome is not whether interstitial fluid can detect the presence of a drug or sweat, but whether sufficient pharmacokinetic trajectories are present in the dataset to support dose adjustments and/or monitoring of patient compliance or toxicity prevention. This type of system will require an accurate record of drug dosing timing, formulation details, and renal and hepatic variables affecting drug levels, as well as an appropriate reference sample collected during development, to ensure that a model can be built to separate pharmacokinetics from sensor drift. The output from this type of system should be a clinically interpretable estimate of drug exposure, rather than an uncalibrated recording [105,106].

An endocrine-rhythm blueprint should be considered from the perspective that time is biochemistry [158,159]. All hormones (cortisol, estradiol, progesterone, etc.) exist on a continuum across circadian, ultradian, menstrual, stress, and medication-induced rhythms. An endocrinology wearable platform should include time of day, individual sleep/wake state [160,161], activity level during the monitoring period, and acute stressors, along with descriptive metadata associated with the sampling matrix. The model should not convert rhythm deviations to pathology. Instead, the model should learn the individual-specific rhythm envelopes and the steady-state disruptions to the subject’s rhythm envelopes, and it will communicate when the sampling matrix or sensor does not contain sufficient data to derive an interpretation of the rhythmic event [162,163]. Table 13 lists the context streams and validation endpoints associated with the above.

Recent human evidence illustrates both the progress and the remaining claim boundaries of these translational blueprints. iCares evaluated refreshed multiparameter wound-exudate sensing directly in patients with chronic wounds [11]; Stressomic and a multimodal cortisol–physiology platform resolved responses to prospectively defined human stress challenges [12,99]; a Parkinson-focused patch recorded sweat L-DOPA and associated molecular trajectories in healthy participants and patients [13]; and continuous microneedle lactate monitoring reached early evaluation in critically ill patients [89]. These studies move the field beyond artificial-fluid feasibility. Still, none has yet demonstrated through an independent prospective trial that acting on the wearable output improves treatment selection, safety, recovery, or patient outcomes.

## 12. Minimum Reporting Standards for Reproducible Wearable Biochemical Sensing

Wearable biosensing is a developing field in need of standardized reporting [164], similar to that used in clinical trials and diagnostic-accuracy studies [133,165]. AI-centered diagnostic-accuracy studies should additionally follow STARD-AI by reporting dataset practices, the complete AI index-test specification, evaluation procedures, algorithmic-bias and fairness assessments, and evidence relevant to applicability and generalizability [166]. STARD-AI complements rather than replaces STARD 2015 and should be used together with TRIPOD + AI when the study also develops or evaluates an individual-level prediction model. For the device aspect of a wearable sensor, the authors recommended the following: electrode geometry, active area, materials, immobilization chemistry, membrane composition, reference-electrode design, substrate mechanical properties, encapsulation, adhesives, sampling interface, fluid volume, and manufacturing variability. For the assay aspect of a wearable sensor, the authors recommended the following: calibration fluid, validation on actual samples, response time, reversibility, drift, selectivity, limit of quantification, dynamic range, interference panel, and temperature and pH dependence. These device criteria will enable more straightforward comparison and replication of promising results. Furthermore, for the on-body aspects of a wearable sensor, the authors have recommended consideration of the following: site on the body, skin preparation, duration of wear, participant demographics, activity protocol, environmental conditions, sweat induction method, sweat-rate estimation, patch failure rate, skin adverse events, and missing data. Many publications document successful traces without reporting the number of failed patches, participants excluded, or unusable time windows, which gives an overall impression of high readiness. Reporting for AI should also consider failures as data. For example, bubbles in the microfluidic path, adhesive failures due to excessive sweating, or transients during flexing should be included in comprehensive quality-control models [21,24]. They should not be treated as a nuisance.

Additionally, the authors state that raw data streams and preprocessing should be adequately described to allow for an independent review of results. The authors recommend that the following datasets be adequately described to allow for independent review: sampling frequency, filtering, baseline adjustments, smoothing, calibration equations, time alignment of data, outlier treatment, and treatment of missing data. When developing wearable sensors using ML, the development of training/test splits, subject-level splits, cross-validation methods, hyperparameter selection, feature construction, and measures to prevent leakage in model assessment should be described [23,24]. Although a model trained on a participant’s data may appear accurate compared to nearby windows, it could fail when applied to a new user.

When reporting comparators, the following parameters are essential for reference measurements: measurement time, measurement matrix, sample acquisition method, laboratory variability, and sample handling. When comparing blood levels with sweat/interstitial-fluid samples, the authors recommend providing the expected lag time and compartment considerations. If the intended use of the output is a trend rather than a concentration level, it is important to provide an appropriate method for trend and event-time agreement, as well as decision agreement. A single correlation coefficient is rarely sufficient in these applications [119,120]. Appropriate reporting methods for agreement can also be provided in calibration plots, and techniques like Bland–Altman analysis can depict points of agreement and regions where the concentration is erroneous, as well as between-group performance and uncertainty of coverage for each group. The authors supply a detailed checklist for practical reporting of AI-ready wearable sensors in Table 14 [133,167,168].

## 13. Ethical Deployment, User Communication, and Trust

The concept of responsible use should be established as an essential aspect of design. Ongoing biochemical monitoring may provide information about medication timing, alcohol use, stress rhythms, reproductive status, metabolic behavior, effects of sleep disturbances, and adherence to therapy. Data from the device may be considered private even though the device was developed for wellness purposes. Trusted systems must utilize the principles of privacy by design; clearly defined consent; local processing whenever possible; minimal data sharing; secure transmission; and control over whether/how data can be shared [144,169]. Researchers need to indicate who can access raw, processed, and model-derived data.

Providing information to the user is particularly important because biochemical data carries medical authority. An individual may feel anxious and attempt self-treatment, with variable results, when the measurement is exploratory. An interface should provide the information necessary to determine what is known, what is uncertain, and what action is appropriate. For example, a hydration tool could have indicated that it had detected the wearer had lost a large amount of sweat based on reliable sampling. It would then provide the individual with a fluid and electrolyte plan. Low confidence in a specific measurement will provide a compelling reason for the wearer to check the patch rather than interpret physiological signals. A stress measurement by an individual should not indicate pathology based on a single cortisol reading, without considering longer-term trends and contextual information about the individual. The ethical risk is that an individual who receives a false alarm may find comfort in the absence of one. Continuing to wear a device that has not detected a danger due to poor sampling, a model being out of distribution, or an analyte having no causal relationship to the individual’s condition will delay care. A decision-support output should include conservative fail-safe rules that clearly state when clinical evaluation is warranted. In high-risk environments such as drug dosing, heat illness, and wound infection, alert thresholds should be validated prospectively, along with defined escalation pathways [119,134]. Data ownership and secondary uses of the data must be accompanied by clear disclosure language. Longitudinal biochemical datasets will play an important role in both research studies and commercial model development. Individuals should be aware of how long their data will be stored, whether it will be used only for immediate feedback, how long it will be used for model improvement, and for what purposes it will be used in the future (e.g., clinical care, research). The occupational and athletic environments are very relevant because both athletes and workers may feel compelled to share their physiological data. Defining the boundaries between safety support and surveillance will help minimize the ethical implications of deploying a wearable device [36,169].

## 14. Limitations of This Review

This review uses a narrative framework rather than a systematic review or meta-analysis methodology [168]. This review aims to integrate design principles from a rapidly evolving interdisciplinary area and to establish a practical measurement-chain framework for AI (artificial intelligence)-ready wearable biochemical monitoring. Therefore, the examples used in the literature are illustrative rather than exhaustive [5,7]. Emphasis is placed on translational lessons that recur across various aspects of biofluid sampling, molecular recognition, flexible integration, data architecture, and clinical validation. There are several limitations in this area of research; one is the heterogeneity of performance metrics for wearable biochemical sensing devices. Studies vary in the biofluid used, body location, induction/extraction methods, calibration media, target ranges, subject types, duration of wear, timing of comparators, and statistical analyses used to evaluate comparators. For example, a low detection limit in a buffer may have little clinical relevance compared with stable performance in real samples, and a high correlation in controlled challenge studies may not predict physician decision-making in daily life. For these reasons, the tables presented in this article emphasize categories of evidence related to performance metrics and the limitations of claims about device use [18,24], rather than ranking devices by a single performance metric. A second limitation is the limited evidence of clinical utility of many biomarkers (beyond glucose) that can be detected using wearable biochemical sensors. There is a wealth of established analytical and in-use performance evidence; however, relatively few prospective clinical studies demonstrate that acting on wearable output improves clinical decision-making, workflow, or outcomes. The lack of clinical evidence should be viewed as the next stage in the translational pathway, rather than the failure of the field. Future studies will focus on connecting the outputs from wearable sensing with the intended use of the sensing data; the behavior of the user related to using the wearable; the intended clinical outcome of acting upon the data; how uncertainty related to the output is communicated to users; and how to respond to failures of the wearable system. It is also important to clarify that AI readiness is not synonymous with automated clinical decision-making. In many near-term wearables, the most significant role for AI will be in quality assurance of sensor outputs. Clinical automation should only be used in settings that meet four criteria: devices have strong biofluid validity and strong evidence supporting clinical validation; the use of the device is associated with controls that limit the risk of harm to users; there are guidelines for the lifecycle governance of the device; and there are protocols for training and educating users regarding the care, use, and limitations of the device. The conservative claims structure within the framework is intended to guide the design of future bioelectronics and intelligent biosensing systems to enhance safety, reproducibility, and clinical benefit [25,28].

## 15. Conclusions

Multimodal, AI-enabled continuous monitoring of molecules via wearable biochemical sensors is advancing beyond glucose sensing alone. One of the barriers may no longer be the isolated demonstration of flexible sensors or single-analyte detection but rather the integration of biofluids (e.g., sweat, interstitial fluid, saliva, tears, exudate from wounds, or breath), recognition chemistry, flexible materials, electronics, metadata, sensor fusion, and validation into a trusted chain of measurements that provide continuous monitoring and adhere to meaningful clinical outcomes. To establish unique opportunities associated with the use of each of the above-mentioned biofluids, the sampling dynamics and matrix effects of these fluids must be explicitly represented in models. The interfaces that provide recognition need to be stable, reversible, and contextually corrected. Accordingly, multi-analyte panels need to be designed based on the relevant clinical questions rather than on analyte concentrations. Although artificial intelligence can assist with converting noisy, multimodal data into interpretable digital biomarkers, these methodologies will only succeed if the associated data is “AI ready” (i.e., consists of synchronized raw data streams, metadata, signal-quality indices, calibration histories, estimates of uncertainty, comparator alignment, diverse validation cohorts, and lifecycle monitoring). The validity of a wearable system when translated to clinical practice comprises six components: analytical validity, biofluid validity, robustness in use-on-body, clinical validity, clinical utility, and post-marketing learning. The most effective systems in the future will include the following elements: sensitivity in detecting molecules, control over the sampling process, rigorous human-centered design, and responsible algorithms. For investigators wishing to prepare submissions for consideration within next-generation submissions, the one key practical recommendation is to provide a complete chain of evidence in the reporting of a study, including descriptions of how the fluid was obtained, as well as how time was maintained, chemistry stabilized, artifacts identified, uncertainty modeled, and comparators aligned, and how the study output will change the outcome decision. Thus, a manuscript that presents the complete chain of evidence will be more robust than one that only reports a single sensitivity value or an attractive trace. This systems perspective will provide a basis for comparison, challenge, adaptation, and clinical validation of future studies utilizing next-generation wearable biochemical systems. The general focus of this Special Issue on wearable bioelectronics and intelligent biosensing systems is that flexibility and intelligence should be evaluated together: devices that exhibit flexibility without biochemical dependability will be comfortable but lack clarity; likewise, devices that exhibit intelligence without adequate control over sampling or validation will produce highly confident artifacts. Integration of soft interfaces, stable chemistry, transparent data architecture, and clinically validated claims will yield the most durable outcomes in this area; consequently, the future of wearable biosensors will entail continuous monitoring based on the same standards that have made glucose monitoring clinically valid. Therefore, the proposed framework should serve as a standard for reporting and designing future studies of wearable biochemical sensors. Researchers conducting high-quality reviews and original studies using wearable biosensors must demonstrate the complete evidence chain (i.e., why the analyte is important; why the selected biofluid is appropriate; how the time course is preserved; how the recognition interface remains interpretable; how metadata are gathered; how models are validated without leakage; how uncertainty is communicated; and what impact the study output will have on decision-making). By including the above components in their research and/or publishing high-quality reviews and articles on wearable biochemical sensors, researchers will contribute durable value to the field of wearable biosensors by providing reusable standards rather than isolated demonstrations. The full list of abbreviations used in this manuscript is included in Table 15.

## Figures and Tables

**Table 1:** Scope, inclusion logic, and evidence extraction fields used to structure this framework review [23,24].

Review Dimension	Primary Inclusion Logic	Evidence Fields Extracted	Why It Matters for AI-Ready Translation
Biofluid and sampling	Reports that explicitly define access to sweat, interstitial fluid, saliva, tears, wound exudate, or breath condensate.	Sampling route, flow or volume control, chronology, induction or extraction method, comparator fluid, and expected lag.	The biofluid is the first transformation in the measurement chain and determines how a molecular signal should be interpreted.
Recognition and transduction	Enzymatic, ion-selective, aptamer, molecularly imprinted, immunoassay, transistor, optical, electrochemiluminescent, and SERS systems.	Target analyte, matrix, reversibility, dynamic range, interferents, drift, regeneration, fouling, and environmental compensation.	AI models can only compensate for known failure modes if the interface exposes them through controls and metadata.
System integration	Flexible, printed, textile, microneedle, microfluidic, wireless, self-powered, or edge-processing platforms.	Wear duration, adhesion, electronics, power, data transfer, self-test routines, reusable/disposable partition, and failure reporting.	Clinical use depends on the whole wearable system, not on an isolated electrode or assay chemistry.
Human and clinical evidence	On-body, volunteer, field, patient, or workflow studies, including negative or limited evidence when available.	Cohort size, comparator, task, endpoint, subgroup coverage, missingness, and real-world perturbations.	Validation should reflect intended use; laboratory traces alone cannot support clinical claims.
Data and AI readiness	Studies and reviews that discuss sensor fusion, calibration transfer, uncertainty, learning systems, or software governance.	Raw-data retention, metadata, quality labels, subject-level splits, external validation, uncertainty metrics, and update policy.	A wearable becomes intelligent only when data provenance, model behavior, and lifecycle performance remain auditable.

**Table 3:** Biofluid compartments for continuous wearable biochemical monitoring [39,40].

Biofluid/Access Route	High-Value Analyte Classes	Dominant Confounders	Translation Fit and Claim Boundary
Sweat/skin-surface microfluidics	Electrolytes, lactate, uric acid, cortisol candidates, drugs, nutrients, hydration, and heat-stress markers.	Sweat rate, gland metabolism, duct reabsorption, evaporation, contamination, skin temperature, body site, and patch occlusion.	Strong for non-invasive trend monitoring, exercise, occupational heat stress, and selected screening applications. Requires flow-aware interpretation and should not be treated as a universal blood surrogate.
Interstitial fluid/microneedles, reverse iontophoresis, or microdialysis.	Glucose, lactate, alcohol, ketones, electrolytes, drugs, and selected hormones.	Transport lag, tissue response, insertion depth, fouling, local inflammation, calibration transfer, and sterility.	Strong for minimally invasive systemic monitoring and therapeutic applications. Requires reference alignment and intended-use-specific lag modeling.
Saliva/mouthguard or oral patch	Hormones, cortisol, drugs, metabolites, oral inflammatory and infectious markers.	Food and drink, pH, enzymes, mucins, microbiome, oral hygiene, dilution, and mechanical abrasion.	Best for targeted oral, behavioral, endocrine, or pharmacokinetic applications. Long-term wearability and hygiene are major barriers.
Tears/contact lens or periocular device	Glucose candidates, lactate, electrolytes, and ocular inflammatory markers.	Low volume, blinking, tear turnover, lens movement, ocular safety, and comfort.	Best for ocular disease and selected systemic research questions. Regulatory and comfort constraints are high.
Wound exudate/smart dressing	pH, uric acid, lactate, oxygen, proteases, cytokines, bacterial metabolites, and temperature.	Local heterogeneity, infection burden, exudate volume, dressing effects, and biofouling.	Strong for local tissue-state monitoring. Does not need to mimic blood if the endpoint is wound healing, infection risk, or dressing decision.
Breath condensate or mask-based sampling	Volatile compounds, respiratory biomarkers, oxidative stress markers, and humidity-related signatures.	Condensation efficiency, humidity, breathing pattern, environmental exposure, and dilution.	Promising for respiratory and metabolic screening, but continuous quantitative calibration and analyte stability remain challenging.

**Table 4:** Sampling technologies and AI-readiness implications [45,47].

Sampling Strategy	Strengths	Key Limitations	AI-Ready Metadata to Capture
Passive sweat collection with an absorbent layer	Simple, inexpensive, comfortable, and compatible with colorimetric or electrochemical patches.	Integrates old and new sweat, uncertain timing, evaporation, and variable dilution.	Wear time, saturation estimate, temperature, humidity, patch occlusion, skin preparation, and removal time.
Soft microfluidic sweat routing	Chronological sampling, reduced contamination, flow estimation, multiplexed reservoirs, and visual quality control.	Fabrication complexity, air bubbles, dead volume, leakage, and low-flow failure.	Channel fill time, reservoir identity, front velocity, bubble or leakage flag, body site, and skin temperature.
Iontophoretic sweat induction	Enables measurements at rest and allows controlled stimulation for selected tests.	May alter local physiology, dilute analytes, irritate skin, and require power/safety controls.	Current profile, stimulant dose, induction duration, skin impedance, rest/exercise state, and adverse skin events.
Solid microneedle electrodes	Direct interstitial-fluid access, fast electrochemical readout, and compact integration.	Insertion mechanics, sterilization, fouling, tissue response, and pain perception.	Insertion site, force or applicator state, wear duration, impedance/contact, device age, and local irritation.
Hollow or hydrogel microneedle extraction	Can collect fluid for reagent-assisted assays and multiplex chemistry.	Variable extraction rate, clogging, swelling, storage, and microfluidic handling.	Extracted volume, pressure, or swelling state, chamber identity, flow history, and time since insertion.
Droplet microfluidic wet chemistry	Controlled assays, reagent integration, repeated measurements, and improved specificity for difficult targets.	Reagent stability, cartridge complexity, timing control, and optical/electrochemical alignment.	Droplet timestamp, reagent lot, incubation time, temperature, background signal, and assay validity flag.
Smart wound dressing	Local monitoring within an existing clinical interface; highly relevant to infection and healing.	Biofouling, exudate heterogeneity, dressing materials, and clinical handling.	Dressing age, wound site, exudate volume, image/clinical score, temperature, and dressing-change event.

**Table 5:** Recognition and transduction modalities for beyond-glucose wearable sensing [64,72].

Modality	Representative Targets	Main Advantages	AI-Readiness Requirement
Enzymatic amperometry	Lactate, glucose, alcohol, uric acid, glutamate, and ketones.	High selectivity for compatible metabolites, mature electronics, and real-time readout.	Track enzyme age, mediator or oxygen dependence, pH, temperature, redox interferents, drift, and membrane hydration.
Potentiometric ion-selective electrodes	Na^+^, K^+^, Cl^−^, NH^4+^ and pH.	Low power, direct electrolyte monitoring, and a strong role as both a biomarker and control stream.	Maintain reference-electrode state, membrane conditioning, hydration, temperature, and ionic-strength context.
Aptamer-based electrochemical sensing	Cortisol candidates, estradiol, drugs, proteins, and nucleic acid-related targets.	Potentially reversible and reagentless affinity readout; adaptable target selection.	Model binding kinetics, nonspecific adsorption, nuclease exposure, regeneration, baseline drift, and matrix effects.
Molecularly imprinted polymers	Cortisol, hormones, drugs, nutrients, and metabolites.	Synthetic robustness, low cost, and compatibility with flexible electrodes.	Validate cross-reactivity, template removal, batch reproducibility, regeneration, and real-sample response.
Immunoassay microfluidics	Cytokines, CRP, proteins, pathogens, and wound markers.	High specificity and sensitivity for large biomolecules.	Record reagent lot, incubation, wash state, temperature, timing, capture-surface history, and assay validity.
Organic electrochemical and field-effect transistors	Ions, metabolites, hormones, and proteins with functionalized channels.	Amplification at soft ionic–electronic interfaces and low-voltage operation.	Separate device physics from analyte binding; track hydration, ionic strength, hysteresis, and transistor drift.
Colorimetric, fluorometric, and electrochemiluminescent patches	pH, chloride, lactate, glucose, ions, enzymes, and selected inflammatory markers.	Visual readout, multiplexed imaging, and smartphone compatibility.	Control illumination, camera calibration, reagent aging, optical background, image processing, and sampling chronology.
SERS and spectroscopic fingerprints	Multiplexed metabolites, drugs, and molecular signatures.	Label-free or low-label multiplexity with high chemical information content.	Standardize substrates, hotspots, spectral preprocessing, sweat chronology, model calibration, and uncertainty reporting.

**Table 6:** Highest human evidence, current claim boundaries, and unresolved translation barriers for beyond-glucose wearable biomarker targets [5,6].

Biomarker Target	Highest Human Evidence, Period, and Volume	Current Evidence-Supported Claim Boundary	Evidence Required for the Next Level	Principal Unresolved Translation Barriers
Electrolytes, principally Na^+^, Cl^−^, and K^+^	HEL2; 2010–2026; nOB ≥ 30 [8,10,52]	Context-aware assessment of local sweat electrolyte concentration and sweat loss under a validated protocol. The output should not be interpreted directly as serum electrolyte concentration or whole-body electrolyte balance.	Multisite validation of a locked device and claim in independent intended-use cohorts, followed by prospective testing of electrolyte-guided action.	Sweat-rate and glandular-reabsorption effects; body site, acclimation, exercise, temperature and humidity; low- or absent-sweat conditions; stimulated versus natural sweat; old–new sweat mixing, evaporation and skin contamination; reference-electrode drift; lot calibration and limited external validation.
Lactate/uric acid	Lactate: HEL2; 2013–2026; nOB ≥ 20. Uric acid: HEL2; 2020–2026; nOB ≥ 10 [8,10,52,54,89].	Lactate may support exercise-threshold, recovery, or physiological-trend assessment; uric acid may support exploratory purine-response or gout-associated trend monitoring. Neither is established as an interchangeable blood measurement.	For each analyte, replicate a locked claim and threshold in at least two independent intended-use cohorts, followed by a sensor-guided intervention study.	Lactate: Eccrine-gland production, sweat-rate and exercise-mode dependence, delayed sweating, enzyme stability, pH, temperature and drift. Uric acid: Low concentration, electroactive interference, pH and dietary effects, serum–sweat lag, renal-function confounding, uricase stability and small short-term patient cohorts.
Alcohol/ketones	Alcohol: HEL4; 1992–2025; nOB ≥ 30. Ketones: HEL2; 2021–2026; nOB ≥ 5 [14,87,88,96,97].	Transdermal alcohol monitoring can identify drinking episodes and has supported sensor-contingent interventions that reduce heavy drinking. Ketone sensors support continuous β-hydroxybutyrate trend and reference-comparison studies but not sensor-guided diabetic ketoacidosis management.	Alcohol: Confirm durability, acceptability, harms, cost-effectiveness, and routine-care generalizability beyond incentive-dependent settings. Ketones: Independent intended-population validation at clinical action thresholds, followed by sensor-guided treatment or prevention trials.	Alcohol: Skin-permeability variation, temperature, environmental alcohol, lag relative to blood or breath, nonwear, tampering, privacy, stigma and forensic interpretation. Ketones: ISF–blood lag, precision across the high β-hydroxybutyrate range, enzyme/cofactor stability, calibration transfer, multiday drift, manufacturing-lot variation, alarm burden and limited evidence in pediatric, home-use and euglycemic-DKA populations.
Cortisol/sex hormones	Cortisol: HEL2; 2018–2026; nOB ≥ 20. Sex hormones, principally estradiol: HEL2; 2023–2024; nOB ≥ 1 [12,98,99].	Research use for circadian, stress-response and reproductive-hormone trajectories with matrix-specific correction and reference comparison. Current evidence does not support endocrine diagnosis or treatment selection.	Multisite validation of a locked assay and interpretation rule across relevant circadian phases, demographic groups and clinical populations, followed by demonstration of decision or patient benefit.	Biofluid–blood partitioning and lag; circadian and ultradian variability; stress-, exercise- and sweat-rate dependence; steroid cross-reactivity; picomolar concentrations; fouling, regeneration and drift; menstrual-cycle, contraceptive and demographic heterogeneity; very limited sex-hormone replication.
Amino acids/vitamins	Amino acids: HEL2; 2019–2026; nOB ≥ 5. Vitamins: HEL2; 2020–2026; nOB ≥ 5.	Monitoring of intake- or exercise-associated molecular dynamics and exploratory sweat–serum relationships. Deficiency diagnosis, dietary prescription and supplementation dosing are not established.	Independent replication of analyte-specific transport relationships and prospectively defined deficiency or response thresholds, followed by sensor-guided nutritional intervention studies.	Trace concentrations; chemically diverse targets; cross-reactivity; diet-, exercise-, sweat-rate-, pH- and body-site dependence; incomplete sweat–serum transport models; vitamin oxidation and stability; lot reproducibility; evidence often originating from one platform or research group.
Drugs	HEL2; 2018–2026; nOB ≥ 10; the strongest evidence concerns L-DOPA and first-in-human antibiotic sensing [13,100].	Pharmacokinetic feasibility, exposure trends and exploratory adherence monitoring. Evidence does not yet support autonomous dose selection or therapeutic titration.	External evaluation of the same locked sensor configuration in independent target-patient cohorts, followed by a prospective sensor-guided dosing trial with safety and outcome endpoints.	Drug-specific sweat or ISF partitioning, protein binding and lag; renal and hepatic function; narrow therapeutic windows; biofouling and calibration drift; microneedle insertion variability and biocompatibility; polypharmacy interference; short wear duration; small cohorts and heterogeneous platforms.
Cytokines/proteins	HEL2; 2020–2025; nOB ≥ 10	Exploratory local or systemic inflammatory trends, disease-activity association and molecular phenotyping using an appropriate reference assay. Diagnostic replacement and treatment escalation are unsupported.	Replication of a locked output and threshold in independent disease cohorts, with prospective evaluation of whether use of the result improves clinical decisions or outcomes.	pg/mL abundance; proteolysis, adsorption and biofouling; affinity-reagent stability and reversibility; sweat-rate dilution; uncertain serum–sweat kinetics; saturation; comparator timing; medication and circadian confounding; small single-center cohorts; possible cohort overlap and absent validated action thresholds.
Wound markers	HEL2; 2011–2026; nOB/in situ ≥ 5 [11]	Direct local monitoring of pH, oxygen, reactive species, temperature and selected inflammatory signals, together with exploratory wound-severity or healing-time classification. Treatment selection has not been prospectively validated.	Independent external validation of a locked wearable output in representative wound types, followed by a trial in which sensor-guided management improves healing, infection control, safety or workflow.	Spatial and etiological wound heterogeneity; biofilm; variable exudate flow and viscosity; old–new exudate mixing; pH and oxygen gradients; fouling and calibration; dressing pressure and sensor contact; sterilization and biocompatibility; nonspecific inflammatory signals; absent reference standards and action thresholds.

HEL values are claim-specific and must not be transferred from one analyte to another within a combined family. nOB denotes the conservative cumulative lower-bound number of independent studies involving actual human wear or direct in situ clinical sensor contact . Human-sample-only feasibility studies do not increase nOB. Study volume describes the size of the published evidence base and does not itself establish clinical validity, clinical utility, AI readiness, regulatory authorization, or completion of the stage-gate pathway. HEL4 for alcohol applies only to transdermal-sensor-contingent alcohol-reduction interventions.

**Table 7:** Representative wearable platforms extending beyond glucose-only monitoring: analytes, sensing principles, biofluid access, reported on-body duration, AI/data-processing methods, major performance, and key limitations.

Study/Platform [Reference]	Target Analyte(s)	Sensing Principle	Biofluid/Access Route	Reported on-Body Duration ᵃ	AI/Algorithmic Method ᵇ	Major Reported Performance ᶜ	Key Translational Limitation
Imani et al., Chem–Phys patch (2016) [95]	Lactate; ECG/heart rate	LOx–Prussian Blue amperometry combined with bipolar Ag/AgCl ECG electrodes and BLE telemetry	Exercise-induced sweat; epidermal ECG	15–30 min intense cycling plus a 3 min cool-down	None; conventional heart-rate extraction and synchronized signal acquisition	Lactate sensitivity: 96 nA mM^−1^ over 0–28 mM; heart rate versus commercial monitor: *r* = 0.975	Only three healthy male participants; short exercise-only protocol; no paired reference assay for on-body sweat lactate or sweat-rate normalization
Nightingale et al., droplet-microfluidic tissue sensor (2019) [51]	Glucose or lactate	Dermal microdialysis coupled to segmented-droplet enzymatic wet chemistry and optical absorbance detection	Dermal tissue microdialysate	Glucose protocol: approximately 60 min baseline plus up to 180 min after oral challenge (≈4 h); glucose *n* = 5 and lactate *n* = 4	None; mechanistic two-compartment modeling, two-point calibration and conventional smoothing	Measurements approximately every 3 s; LODs of 68 μM for glucose and 17 μM for lactate	Invasive microdialysis probe; pump, reagents, carrier oil and waste handling required; palm-sized instrumentation; small healthy cohorts and no numerical MARD/comparator-correlation endpoint
Tehrani et al., integrated microneedle array (2022) [49]	Glucose plus lactate or glucose plus alcohol	Enzyme-functionalized solid microneedle amperometry with integrated wireless readout	Dermal interstitial fluid	Up to 300 min (≈5 h); nine healthy adults	None; retrospective two-point calibration and app-based visualization	MARD: 9.6% for glucose, 16.1% for lactate and 15.7% for alcohol	Small healthy cohort; retrospective calibration; hours rather than multiday wear; insertion variability, biofouling and population-level validation remain unresolved
Wang et al., NutriTrek (2022) [93]	Tyr, Trp, Leu/BCAAs and other nutrients/metabolites	Laser-engraved graphene–MIP interfaces; direct or redox-active-reporter-mediated voltammetry; in situ regeneration, iontophoresis and microfluidic sampling	Exercise- or iontophoresis-induced sweat	60 min cycling protocol or until fatigue; approximately 100 min monitoring traces in exercise/rest studies	None; iterative/polynomial baseline correction, automatic peak extraction and deterministic temperature/electrolyte calibration	Sweat–serum correlation: *r* = 0.66 for Leu and *r* = 0.69 for total BCAA (*n* = 65 paired measurements)	Only moderate serum–sweat correlations; small pilot groups, including only three participants with obesity and T2DM; clinical risk interpretation was not externally validated
Ye et al., estradiol aptamer patch (2024) [98]	Estradiol; temperature, pH and ionic strength as correction channels	Target-induced strand-displacement aptamer on an AuNP–MXene electrode; SWV, iontophoresis and capillary microfluidics	Iontophoresis-induced sweat	5 min iontophoresis plus 60 min sampling/incubation/sensing: 65 min per test	None; deterministic multivariate calibration using temperature, pH and ionic strength	LOD: 0.14 pM; sensor–ELISA *r* = 0.921 (*n* = 25); sweat–serum *r* = 0.837 (*n* = 51)	Menstrual-cycle trajectories were obtained from only two women and real-time tests from three; the 60 min assay is an intermittent measurement rather than demonstrated continuous long-term monitoring
Spinelli et al., hydration EBS device (2025) [86]	Sodium concentration/loss, sweat volume/rate; skin temperature, heat flux and activity	Microfluidic Ag/AgCl conductance/volume electrodes integrated with thermal and inertial sensors and haptic feedback	Sweat	Controlled validation: 60–90 min (*n* = 22); one representative field trace: approximately 9 h	None; deterministic 2% °C^−1^ correction, regional-to-whole-body scaling, median filtering and rule-based haptic thresholds	Sodium versus reference electrode: *R*^2^ = 0.85 (*n* = 13); whole-body sweat rate versus gravimetry: *R*^2^ = 0.76 (*n* = 22)	Nine of 22 participants produced insufficient sweat for the sodium comparator; active sweating is required, and local-to-whole-body scaling remains site- and population-dependent
Jeon et al., CEP–SERS patch (2025) [82]	Uric acid, lactate and tyrosine	Chronoepifluidic sequential sweat sampling with capillary burst valves and Ag-nanoisland SERS	Exercise-induced sweat	Session-based exercise protocol; a single total wear duration was not reported	Autoencoder plus logistic regression; repeated random-sampling cross-validation and SHAP interpretation	Independent human-sweat validation: *R*^2^ = 0.96 for uric acid and *R*^2^ = 0.86 for lactate; four participants	Only four participants; Raman spectra were acquired after patch removal using a benchtop instrument; no fully integrated real-time optical readout or participant-level external validation
Choi et al., CSPINS neuromorphic system (2025) [101]	Lactate/glucose sensing components; heart rate and core body temperature; sepsis classes	Enzymatic synaptic transistors, printed physical sensors and memristive neuromorphic circuitry	Biochemical channels tested in PBS/model solutions; physiological channels tested on skin	2 h for heart-rate/temperature modules only; integrated biochemical on-body duration not reported	Fixed-threshold printed hardware neural network	Replayed diagnostic-classification accuracy: 84.4%	Clinical biomarker values were converted to simulated voltage inputs; biochemical sensors were not validated in an on-body biofluid, and the reported accuracy is not prospective wearable diagnostic performance

ᵃ On-body duration denotes the reported measurement episode, not analytical response time, sampling interval, battery lifetime, storage stability, or proposed device lifetime. NR indicates that a single continuous wear duration was not reported. ᵇ “None” indicates that no AI/ML model was applied to the wearable data. Deterministic calibration, filtering, conventional regression, pharmacokinetic modeling and threshold-based control are reported but are not classified as AI. ᶜ Performance measures are reproduced as defined in the primary studies. They should not be interpreted as a direct cross-platform ranking because the analytes, matrices, cohorts, comparators and endpoints differ. *r*, Pearson correlation coefficient; *R*^2^, coefficient of determination; MARD, mean absolute relative difference; LOD, limit of detection.

**Table 8:** Materials and integration choices for flexible biochemical wearables [108,109].

Material/Subsystem	Benefits	Failure Modes	Design Mitigations
Elastomeric microfluidics	Soft conformal routing, low-cost molding, optical transparency, and skin compatibility.	Hydrophobic absorption, bubble trapping, delamination, and evaporation.	Surface treatment, barrier layers, vent design, flow markers, and adhesive optimization.
Hydrogels and ion gels	Hydrated interfaces, enzyme immobilization, ion conduction, and comfort.	Drying, swelling, leaching, microbial growth, and mechanical weakness.	Humectants, cross-link control, antimicrobial design, encapsulation, and shelf-life testing.
Printed carbon/metal electrodes	Scalable fabrication, low profile, and compatibility with tattoos/textiles.	Cracking, ink variability, fouling, and reference instability.	Stretchable geometries, quality control, protective membranes, and robust reference chemistry.
Conducting polymers and organic electronics	Soft mixed ionic–electronic conduction, amplification, and low-voltage operation.	Doping drift, hydration sensitivity, hysteresis, and encapsulation challenges.	Stable dopants, control transistors, environmental isolation, and device-physics models.
Textile platforms	Comfort, large area, everyday integration, and sweat-wicking.	Variable contact, laundering, roughness, and uncontrolled fluid routing.	Modular cartridges, wash-stable conductors, and defined microfluidic zones.
Microneedle arrays	Direct ISF access, multiplexed electrodes, and small sample volumes.	Fracture, insertion variability, pain, tissue response, and sterilization needs.	Mechanical testing, insertion aids, sterile packaging, biocompatible materials, and impedance feedback.
Reusable electronics with a disposable assay patch	Cost reduction, sustainability, and replaceable biointerface.	Connector fouling, alignment errors, and user handling variability.	Mechanical keying, self-test routines, sealed contacts, and a simple user interface.

**Table 9:** Minimum AI-ready data package for multimodal wearable biochemical biosensors [17,18].

Data Class	Required Fields	Examples of Failure Prevented	Reporting Expectation
Time and provenance	Absolute timestamp, sampling frequency, clock drift, sensor lot, firmware version, body site, wear start/stop, and preprocessing version.	Misaligned reference samples, hidden device-lot shifts, and irreproducible preprocessing.	Report raw and processed timelines with versioned transformations.
Raw and calibrated signals	Raw current/potential/spectrum/image, calibrated analyte estimate, calibration equation, baseline, dynamic range flag, and saturation flag.	Loss of troubleshooting ability and hidden saturation or clipping.	Retain raw streams and state calibration method for every analyte.
Sampling state	Sweat rate or flow proxy, chamber identity, fill time, induction current, extracted volume, bubble/leak flag, ISF insertion status, and dressing age.	Treating old sweat, absent flow, or failed insertion as physiology.	Record sample provenance as continuous metadata, not a note in methods.
Matrix and environment	Temperature, pH, ionic strength, skin impedance, humidity, motion, pressure, activity class, and ambient heat.	pH/temperature artifacts, motion noise, and environmental confounding.	Use measured correction streams whenever they affect the recognition interface.
Comparator and labels	Reference method, reference matrix, sample time, laboratory error, meal/drug/exercise events, symptom annotations, and label uncertainty.	Training on mislabeled or temporally shifted comparators.	Report comparator timing and uncertainty explicitly.
Quality and uncertainty	Signal-quality index, missingness reason, artifact class, drift estimate, prediction interval, abstention flag, and alert confidence.	False precision, silent data deletion, and unsafe alerts.	Output values with reliability or confidence indicators.
Privacy and governance	Consent category, data-minimization rationale, encryption, access control, model version, update policy, and audit log.	Uncontrolled secondary use, non-auditable model changes, and cybersecurity risk.	Describe data flow and lifecycle governance for any clinical-facing claim.

**Table 10:** Sensor-fusion tasks and validation metrics for AI-ready wearables [116,118].

AI/Analytics Task	Possible Methods	Inputs Required	Validation Metrics Beyond Accuracy
Artifact detection	Rule-based gates, supervised classifiers, autoencoders, and anomaly detection.	Raw signal, motion, impedance, flow, temperature, and device age.	Sensitivity to known artifacts, false rejection rate, recovery time, subgroup behavior, and effect on downstream outputs.
Drift compensation	State-space models, Kalman filters, adaptive baselines, transfer learning, and internal standards.	Calibration history, control channels, repeated standards, or reference events.	Residual drift, uncertainty coverage, performance by sensor age, failure-flag accuracy, and external lot validation.
Lag and temporal alignment	Physiological compartment models, dynamic time warping, and Bayesian filters.	Reference timing, flow, activity, perfusion proxies, and analyte kinetics.	Event timing error, trend agreement, comparator-aligned RMSE, and robustness to sparse labels.
Multimodal state estimation	Bayesian networks, multitask learning, temporal transformers, and hybrid mechanistic models.	Biochemical channels, vitals, activity, environment, symptoms, and context metadata.	Calibration, discrimination, decision-curve utility, false-alert burden, and prospective generalization.
Personalized baselines	Hierarchical models, mixed-effects models, meta-learning, and Gaussian processes.	Repeated user data, population priors, covariates, and device metadata.	Within-person change detection, new-subject validation, bias/variance decomposition, and drift separation.
Uncertainty quantification	Bayesian inference, ensembles, conformal prediction, and probabilistic forecasting.	Training distribution, quality flags, reference uncertainty, and missingness patterns.	Prediction interval coverage, abstention usefulness, risk calibration, and decision consequences.
Privacy-preserving learning	Federated learning, secure aggregation, differential privacy, and split learning.	Local device data, labels, model version, and cohort descriptors.	Central and subgroup performance, update stability, privacy budget, and drift detection.

**Table 11:** Validation gates for clinical translation of multimodal wearable biosensors [25,28].

Validation Gate	Key Question	Minimum Evidence	Common Failure
Analytical validity	Does the sensor measure the intended target under controlled and interferent-rich conditions?	LoD, LoQ, dynamic range, selectivity, interference, response time, drift, reproducibility, stability, and cross-talk.	Excellent buffer performance but weak real-sample performance or unmeasured cross-talk.
Biofluid validity	Does the sampled fluid and sampling method support the physiological claim?	Recovery, flow, lag, matrix effects, contamination, comparator strategy, sampling chronology, and body-site variation.	Assuming sweat, saliva, or ISF equals blood without transport and matrix validation.
On-body performance	Does the device work during realistic wear?	Adhesion, comfort, motion robustness, skin response, body-site variation, usability, missingness, and failure analysis.	Short controlled tests in homogeneous volunteers with failed patches were excluded silently.
Clinical validity	Does the output relate to a meaningful endpoint or reference in the intended population?	Prospective design, predefined endpoints, comparator timing, diverse cohort, subgroup analysis, and frozen algorithm testing.	Post hoc correlations without intended use, action threshold, or subject-level validation.
Clinical utility	Does acting on the output improve decisions, workflow, or outcomes?	Workflow study, alert burden, user comprehension, decision-curve analysis, health-economic evidence, and outcome impact.	Data-rich dashboards that do not safely change care or behavior.
Regulatory and lifecycle governance	Can the system remain safe after deployment and updates?	Risk management, cybersecurity, privacy, model versioning, update policy, post-market drift monitoring, and audit trail.	Opaque AI updates, unmonitored performance drift, and unclear responsibility for alerts.

**Table 12:** Failure modes, diagnostic signals, and design responses [16,144].

Failure Class	Examples	Detectable Diagnostic Signals	Recommended Response
Sampling failure	No sweat, old sweat mixing, bubbles, leakage, low ISF extraction, and chamber blockage.	Flow/front velocity, chamber fill status, impedance, optical markers, extraction pressure, and missingness pattern.	Flag sample state, abstain from analyte interpretation, prompt replacement, or report uncertainty.
Recognition-layer failure	Enzyme aging, receptor saturation, mediator leaching, MIP cross-reactivity, antibody fouling, and SERS substrate drift.	Control electrodes, baseline drift, internal standard, regeneration response, spectral quality, and duplicate-channel disagreement.	Use materials prevention plus quality flags; separate drift from physiology before model fusion.
Mechanical/electrical failure	Cracking, delamination, strain artifacts, water ingress, wireless dropout, battery depletion, and analog front-end saturation.	Strain channel, contact impedance, battery voltage, reset log, packet loss, saturation flag, and temperature excursions.	Pause output, communicate device health status, and maintain an audit trail for post-market analysis.
User-interface failure	Incorrect placement, poor skin preparation, early removal, reapplication, cosmetics, topical medication, and compression.	Contact maps, placement guide, motion/contact mismatch, user annotation, and sudden baseline shifts.	Make correct use intuitive; detect incorrect use and give simple corrective prompts.
Algorithmic failure	Out-of-distribution users, device-lot changes, firmware updates, data leakage, and subgroup bias.	OOD score, calibration drift, subgroup monitoring, model version mismatch, and performance surveillance.	Use conservative abstention, locked validation, controlled updates, and post-market drift monitoring.

**Table 13:** Use-case blueprints linking panels, context streams, and validation endpoints [86,154].

Use Case	Core Biochemical Panel	Context Streams Required	Validation Endpoint and Output
Occupational heat stress	Sweat Na^+^ and Cl^−^, sweat rate, pH, and optional lactate.	Skin/core-temperature proxy, ambient temperature/humidity, thermal flux, activity, work-rest schedule, and symptoms.	Fluid-loss and heat-strain risk with confidence; validation against body mass loss, symptoms, thermal metrics, and work-rest decisions.
Athletic recovery and training load	Lactate, glucose context, ketones, electrolytes, and sweat rate.	Heart rate, HRV, activity class, perceived exertion, sleep, training plan, and environment.	Personalized metabolic stress/recovery feature; validation against performance, recovery metrics, and training outcomes.
Wound monitoring	pH, uric acid, lactate, oxygen, proteases, temperature, and inflammatory markers.	Dressing age, exudate volume, wound image, clinical score, infection signs, and treatment events.	Local deterioration or healing trajectory; validation against microbiology, healing rate, and clinician decisions.
Therapeutic drug monitoring	Drug or metabolite, electrolytes/pH, optional renal or hepatic stress markers.	Dose time, formulation, meals, renal/hepatic covariates, activity, and sensor quality.	Exposure estimate and adherence/toxicity risk; validation against pharmacokinetic reference sampling and dose-adjustment outcomes.
Endocrine rhythm and reproductive health	Cortisol, estradiol, progesterone candidates, and pH/ionic correction variables.	Clock time, sleep, menstrual phase, stressors, medication, and sampling mode.	Personal rhythm feature or cycle-phase support; validation against blood/saliva reference and clinically defined endpoints.

**Table 14:** Practical reporting checklist for AI-ready wearable biochemical biosensor manuscripts [133,165].

Reporting Domain	Minimum Information	Reviewer’s Concern Addressed	Submission-Ready Recommendation
Device and materials	Geometry, active area, substrate, adhesive, encapsulation, electrode/reference design, membrane/receptor composition, and manufacturing variability.	Can another group reproduce the device and understand failure modes?	Provide diagrams, layer stack, fabrication tolerances, and lot-to-lot information.
Assay chemistry	Calibration medium, real-sample validation, LoD/LoQ, range, selectivity, drift, response time, reversibility, temperature/pH dependence, and interferences.	Does buffer performance translate to the target biofluid?	Separate buffer, artificial biofluid, and real-sample results.
Sampling and on-body protocol	Body site, skin prep, wear duration, induction/extraction mode, flow estimate, environmental conditions, cohort descriptors, and adverse skin events.	Were real-world use and failures captured?	Report failures, exclusions, and missingness with reasons.
Data processing and AI	Raw-data retention, filters, calibration equations, time alignment, feature construction, split strategy, model version, and uncertainty method.	Could performance reflect leakage or hidden preprocessing?	Use subject-level or external validation; document pipeline versions.
Clinical claim	Intended use, target population, comparator, endpoint, action threshold, alert logic, and risk controls.	Does the evidence match the claim?	State whether the output is exploratory or for wellness, diagnostic, monitoring, or decision-support purposes.
Figures and reuse	Source, license, permission, modifications, resolution, alt text, and original schematic status.	Are visuals legally reusable and publication-quality?	Use original schematics where possible; give exact licenses and modification statements for third-party figures.

**Table 15:** Abbreviations used in the manuscript [5,15].

Abbreviation	Definition	Abbreviation	Definition
AI	artificial intelligence	BLE	Bluetooth Low Energy
CGM	continuous glucose monitoring	CRP	C-reactive protein
EBS	electrochemical/biophysical sensing	ECL	electrochemiluminescence
GMLP	Good Machine Learning Practice	HRV	heart-rate variability
IMDRF	International Medical Device Regulators Forum	ISF	interstitial fluid
LoD	limit of detection	LoQ	limit of quantification
MIP	molecularly imprinted polymer	ML	machine learning
NFC	near-field communication	OOD	out of distribution
RAR	redox-active reporter	SaMD	Software as a medical device
SERS	surface-enhanced Raman spectroscopy	TNF	tumor necrosis factor

## Data Availability

No new experimental data were generated or analyzed in this review. Original schematics were created for this manuscript. Reused literature figures are attributed in their captions with license information.

## References

[1] Rodbard D. (2016). Continuous glucose monitoring: A review of successes, challenges, and opportunities. Diabetes Technol. Ther..

[2] Danne T., Nimri R., Battelino T., Bergenstal R.M., Close K.L., DeVries J.H., Garg S., Heinemann L., Hirsch I., Amiel S.A. (2017). International consensus on use of continuous glucose monitoring. Diabetes Care.

[3] Battelino T., Danne T., Bergenstal R.M., Amiel S.A., Beck R., Biester T., Bosi E., Buckingham B.A., Cefalu W.T., Close K.L. (2019). Clinical targets for continuous glucose monitoring data interpretation: Recommendations from the International Consensus on Time in Range. Diabetes Care.

[4] Graham C. (2017). Continuous glucose monitoring and global reimbursement: An update. Diabetes Technol. Ther..

[5] Kim J., Campbell A.S., de Ávila B.E.-F., Wang J. (2019). Wearable biosensors for healthcare monitoring. Nat. Biotechnol..

[6] Sempionatto J.R., Lasalde-Ramírez J.A., Mahato K., Wang J., Gao W. (2022). Wearable chemical sensors for biomarker discovery in the omics era. Nat. Rev. Chem..

[7] Brasier N., Wang J., Gao W., Sempionatto J.R., Dincer C., Ates H.C., Güder F., Olenik S., Schauwecker I., Schaffarczyk D. (2024). Applied body-fluid analysis by wearable devices. Nature.

[8] Cho S., Shaban S.M., Song R., Zhang H., Yang D., Kim M.-J., Xiong Y., Li X., Madsen K., Wapnick S. (2024). A skin-interfaced microfluidic platform supports dynamic sweat biochemical analysis during human exercise. Sci. Transl. Med..

[9] Heng W., Yin S., Min J., Wang C., Han H., Sani E.S., Li J., Song Y., Rossiter H.B., Gao W. (2024). A smart mask for exhaled breath condensate harvesting and analysis. Science.

[10] Shin S., Liu R., Yang Y., Lasalde-Ramírez J.A., Kim G., Won C., Min J., Wang C., Fan K., Han H. (2025). A bioinspired microfluidic wearable sensor for multiday sweat sampling, transport, and metabolic analysis. Sci. Adv..

[11] Wang C., Fan K., Shirzaei Sani E., Lasalde-Ramírez J.A., Heng W., Min J., Solomon S.A., Wang M., Li J., Han H. (2025). A microfluidic wearable device for wound exudate management and analysis in human chronic wounds. Sci. Transl. Med..

[12] Tu J., Yeom J., Ulloa J.C., Solomon S.A., Min J., Heng W., Kim G., Dao J., Vemu R., Pang M. (2025). Stressomic: A wearable microfluidic biosensor for dynamic profiling of multiple stress hormones in sweat. Sci. Adv..

[13] Zhao H., Bai J., Zhang X., Li D., Chang Q., Xia Y., Liu Y., Mugo S.M., Wang H., Zhang Q. (2025). A fully integrated wearable sweat sensing patch for online analysis of multiple Parkinson’s disease-related biomarkers. Adv. Mater..

[14] Saha T., Ding S., Qin S., Fishman H., Pham R., Gu T., Lin M., Xian Y., Sabbagh B., Abdal A. (2026). A fully integrated smart ring for daily biochemical monitoring. Nat. Commun..

[15] Heikenfeld J., Jajack A., Feldman B., Granger S.W., Gaitonde S., Begtrup G., Katchman B.A. (2019). Accessing analytes in biofluids for peripheral biochemical monitoring. Nat. Biotechnol..

[16] Ray T.R., Choi J., Bandodkar A.J., Krishnan S., Gutruf P., Tian L., Ghaffari R., Rogers J.A. (2019). Bio-integrated wearable systems: A comprehensive review. Chem. Rev..

[17] Coravos A., Khozin S., Mandl K.D. (2019). Developing and adopting safe and effective digital biomarkers to improve patient outcomes. npj Digit. Med..

[18] Goldsack J.C., Coravos A., Bakker J.P., Bent B., Dowling A.V., Fitzer-Attas C., Godfrey A., Godino J.G., Gujar N., Izmailova E. (2020). Verification, analytical validation, and clinical validation (V3): The foundation of determining fit-for-purpose for biometric monitoring technologies (BioMeTs). npj Digit. Med..

[19] Ye S., Feng S., Huang L., Bian S. (2020). Recent progress in wearable biosensors: From healthcare monitoring to sports analytics. Biosensors.

[20] Sharma A., Badea M., Tiwari S., Marty J.L. (2021). Wearable biosensors: An alternative and practical approach in healthcare and disease monitoring. Molecules.

[21] Ates H.C., Nguyen P.Q., Gonzalez-Macia L., Morales-Narváez E., Güder F., Collins J.J., Dincer C. (2022). End-to-end design of wearable sensors. Nat. Rev. Mater..

[22] Yang Y., Gao W. (2019). Wearable and flexible electronics for continuous molecular monitoring. Chem. Soc. Rev..

[23] Mitchell M., Wu S., Zaldivar A., Barnes P., Vasserman L., Hutchinson B., Spitzer E., Raji I.D., Gebru T. (2019). Model cards for model reporting. Proceedings of the Conference on Fairness, Accountability, and Transparency.

[24] Collins G.S., Moons K.G.M., Dhiman P., Riley R.D., Beam A.L., Van Calster B., Ghassemi M., Liu X., Reitsma J.B., van Smeden M. (2024). TRIPOD+AI statement: Updated guidance for reporting clinical prediction models that use regression or machine learning methods. BMJ.

[25] International Medical Device Regulators Forum (2017). Software as a Medical Device (SaMD): Clinical Evaluation.

[26] U.S. Food and Drug Administration (2023). Digital Health Technologies for Remote Data Acquisition in Clinical Investigations: Guidance for Industry, Investigators, and Other Stakeholders. Final Guidance. https://www.fda.gov/regulatory-information/search-fda-guidance-documents/digital-health-technologies-remote-data-acquisition-clinical-investigations.

[27] Li T., Higgins J.P.T., Deeks J.J., Higgins J.P.T., Thomas J., Chandler J., Cumpston M., Li T., Page M.J., Welch V., Flemyng E. (2024). Chapter 5: Collecting data. Cochrane Handbook for Systematic Reviews of Interventions, Version 6.5.

[28] International Medical Device Regulators Forum (2025). Good Machine Learning Practice for Medical Device Development: Guiding Principles.

[29] Liu X., Rivera S.C., Moher D., Calvert M.J., Denniston A.K. (2020). Reporting guidelines for clinical trial reports for interventions involving artificial intelligence: The CONSORT-AI extension. Nat. Med..

[30] Rivera S.C., Liu X., Chan A.W., Denniston A.K., Calvert M.J. (2020). Guidelines for clinical trial protocols for interventions involving artificial intelligence: The SPIRIT-AI extension. Nat. Med..

[31] Bland J.M., Altman D.G. (2007). Agreement between methods of measurement with multiple observations per individual. J. Biopharm. Stat..

[32] Carstensen B., Simpson J., Gurrin L.C. (2008). Statistical models for assessing agreement in method comparison studies with replicate measurements. Int. J. Biostat..

[33] Zou G.Y. (2013). Confidence interval estimation for the Bland-Altman limits of agreement with multiple observations per individual. Stat. Methods Med. Res..

[34] Parker R.A., Weir C.J., Rubio N., Rabinovich R., Pinnock H., Hanley J., McCloughan L., Drost E.M., Mantoani L.C., MacNee W. (2016). Application of mixed effects limits of agreement in the presence of multiple sources of variability: Exemplar from the comparison of several devices to measure respiratory rate in COPD patients. PLoS ONE.

[35] Field C.A., Welsh A.H. (2007). Bootstrapping clustered data. J. R. Stat. Soc. Ser. B Stat. Methodol..

[36] Zinzuwadia A., Singh J.P. (2022). Wearable devices-addressing bias and inequity. Lancet Digit. Health.

[37] International Medical Device Regulators Forum (2024). Guiding Principles to Support Medical Device Health Equity.

[38] International Council for Harmonisation of Technical Requirements for Pharmaceuticals for Human Use (2023). ICH Harmonised Guideline Q2(R2): Validation of Analytical Procedures. Final Version.

[39] Heikenfeld J. (2016). Non-invasive analyte access and sensing through eccrine sweat: Challenges and outlook circa 2016. Electroanalysis.

[40] Baker L.B. (2019). Physiology of sweat gland function: The roles of sweating and sweat composition in human health. Temperature.

[41] Sonner Z., Wilder E., Heikenfeld J., Kasting G., Beyette F., Swaile D., Sherman F., Joyce J., Hagen J., Kelley-Loughnane N. (2015). The microfluidics of the eccrine sweat gland, including biomarker partitioning, transport, and biosensing implications. Biomicrofluidics.

[42] Basu A., Dube S., Slama M., Errazuriz I., Amezcua J.C., Kudva Y.C., Peyser T., Carter R.E., Cobelli C., Basu R. (2013). Time lag of glucose from intravascular to interstitial compartment in humans. Diabetes.

[43] Cengiz E., Tamborlane W.V. (2009). A tale of two compartments: Interstitial versus blood glucose monitoring. Diabetes Technol. Ther..

[44] Horvath I., Hunt J., Barnes P.J., Alving K., Antczak A., Baraldi E., Becher G., van Beurden W.J., Corradi M., Dekhuijzen R. (2005). Exhaled breath condensate: Methodological recommendations and unresolved questions. Eur. Respir. J..

[45] Koh A., Kang D., Xue Y., Lee S., Pielak R.M., Kim J., Hwang T., Min S., Banks A., Bastien P. (2016). A soft, wearable microfluidic device for the capture, storage, and colorimetric sensing of sweat. Sci. Transl. Med..

[46] Nyein H.Y.Y., Tai L.C., Ngo Q.P., Chao M., Zhang G.B., Gao W., Bariya M., Bullock J., Kim H., Fahad H.M. (2018). A wearable microfluidic sensing patch for dynamic sweat secretion analysis. ACS Sens..

[47] Bandodkar A.J., Gutruf P., Choi J., Lee K., Sekine Y., Reeder J.T., Jeang W.J., Aranyosi A.J., Lee S.P., Model J.B. (2019). Battery-free, skin-interfaced microfluidic/electronic systems for simultaneous electrochemical, colorimetric, and volumetric analysis of sweat. Sci. Adv..

[48] Emaminejad S., Gao W., Wu E., Davies Z.A., Nyein H.Y.Y., Challa S., Ryan S.P., Fahad H.M., Chen K., Shahpar Z. (2017). Autonomous sweat extraction and analysis applied to cystic fibrosis and glucose monitoring using a fully integrated wearable platform. Proc. Natl. Acad. Sci. USA.

[49] Tehrani F., Teymourian H., Wuerstle B., Kavner J., Patel R., Furmidge A., Aghavali R., Hosseini-Toudeshki H., Brown C., Zhang F. (2022). An integrated wearable microneedle array for the continuous monitoring of multiple biomarkers in interstitial fluid. Nat. Biomed. Eng..

[50] Ranamukhaarachchi S.A., Padeste C., Dübner M., Hafeli U.O., Stoeber B., Cadarso V.J. (2016). Integrated hollow microneedle-optofluidic biosensor for therapeutic drug monitoring in sub-nanoliter volumes. Sci. Rep..

[51] Nightingale A.M., Leong C.L., Burnish R.A., Hassan S.-U., Zhang Y., Clough G.F., Boutelle M.G. (2019). Monitoring biomolecule concentrations in tissue using a wearable droplet microfluidic-based sensor. Nat. Commun..

[52] Chen L., Su B., Zhong G., Wang J., Ji Y., Zhou Z., He X., Xu T. (2026). Controlled sweat generation via ultrasound stimulation integrated in a wearable device. Nat. Commun..

[53] Mena-Bravo A., de Castro M.D.L. (2014). Sweat: A sample with limited present applications and promising future in metabolomics. J. Pharm. Biomed. Anal..

[54] Song H., Hong Y.-M., Kim D., Seo H., Park W., Paek J., Lee D., Cho S.K., Ahn S.S., Kim J. (2026). Smart contact lens-trained digital twin for device-free personalized uric acid prediction. Sci. Adv..

[55] Kim J., Valdés-Ramírez G., Bandodkar A.J., Jia W., Martinez A.G., Ramírez J., Mercier P., Wang J. (2014). Non-invasive mouthguard biosensor for continuous salivary monitoring of metabolites. Analyst.

[56] Kim J., Imani S., de Araujo W.R., Warchall J., Valdés-Ramírez G., Paixao T.R.L.C., Mercier P.P., Wang J. (2015). Wearable salivary uric acid mouthguard biosensor with integrated wireless electronics. Biosens. Bioelectron..

[57] Arakawa T., Kuroki Y., Nitta H., Chouhan P., Toma K., Sawada S.-I., Takeuchi S., Sekita T., Akiyoshi K., Minakuchi S. (2016). Mouthguard biosensor with telemetry system for monitoring of saliva glucose: A novel cavitas sensor. Biosens. Bioelectron..

[58] Yao H., Liao Y., Lingley A.R., Afanasiev A., Lahdesmaki I., Otis B.P., Parviz B.A. (2012). A contact lens with integrated telecommunication circuit and sensors for wireless and continuous tear glucose monitoring. J. Micromech. Microeng..

[59] Kim J., Kim M., Lee M.S., Kim K., Ji S., Kim Y.T., Park J., Na K., Bae K.-H., Kim H.K. (2017). Wearable smart sensor systems integrated on soft contact lenses for wireless ocular diagnostics. Nat. Commun..

[60] García-Carmona L., Martín A., Sempionatto J.R., Moreto J.R., González M.C., Wang J., Escarpa A. (2019). Pacifier biosensor: Toward noninvasive saliva biomarker monitoring. Anal. Chem..

[61] Tseng P., Napier B., Garbarini L., Kaplan D.L., Omenetto F.G. (2018). Functional, RF-trilayer sensors for tooth-mounted, wireless monitoring of the oral cavity and food consumption. Adv. Mater..

[62] Maier D., Laubender E., Basavanna A., Schumann S., Güder F., Urban G.A., Dincer C. (2019). Toward continuous monitoring of breath biochemistry: A paper-based wearable sensor for real-time hydrogen peroxide measurement in simulated breath. ACS Sens..

[63] Pleil J.D., Stiegel M.A., Risby T.H. (2013). Clinical breath analysis: Discriminating between human endogenous compounds and exogenous (environmental) chemical confounders. J. Breath Res..

[64] Bobacka J., Ivaska A., Lewenstam A. (2008). Potentiometric ion sensors. Chem. Rev..

[65] Bandodkar A.J., Wang J. (2014). Non-invasive wearable electrochemical sensors: A review. Trends Biotechnol..

[66] Gao W., Emaminejad S., Nyein H.Y.Y., Challa S., Chen K., Peck A., Fahad H.M., Ota H., Shiraki H., Kiriya D. (2016). Fully integrated wearable sensor arrays for multiplexed in situ perspiration analysis. Nature.

[67] Jia W., Bandodkar A.J., Valdés-Ramírez G., Windmiller J.R., Yang Z., Ramírez J., Chan G., Wang J. (2013). Electrochemical tattoo biosensors for real-time noninvasive lactate monitoring in human perspiration. Anal. Chem..

[68] Parrilla M., Cuartero M., Crespo G.A. (2019). Wearable potentiometric ion sensors. TrAC Trends Anal. Chem..

[69] Schazmann B., Morris D., Slater C., Beirne S., Fay C., Reuveny R., Moyna N., Diamond D. (2010). A wearable electrochemical sensor for the real-time measurement of sweat sodium concentration. Anal. Methods.

[70] Baker B.R., Lai R.Y., Wood M.S., Doctor E.H., Heeger A.J., Plaxco K.W. (2006). An electronic, aptamer-based small-molecule sensor for the rapid, label-free detection of cocaine in adulterated samples and biological fluids. J. Am. Chem. Soc..

[71] Lai R.Y., Plaxco K.W., Heeger A.J. (2007). Aptamer-based electrochemical detection of picomolar platelet-derived growth factor directly in blood serum. Anal. Chem..

[72] Ferguson B.S., Hoggarth D.A., Maliniak D., Ploense K., White R.J., Woodward N., Hsieh K., Bonham A.J., Eisenstein M., Kippin T.E. (2013). Real-time, aptamer-based tracking of circulating therapeutic agents in living animals. Sci. Transl. Med..

[73] Arroyo-Currás N., Somerson J., Vieira P.A., Ploense K.L., Kippin T.E., Plaxco K.W. (2017). Real-time measurement of small molecules directly in awake, ambulatory animals. Proc. Natl. Acad. Sci. USA.

[74] Lubin A.A., Plaxco K.W. (2010). Folding-based electrochemical biosensors: The case for responsive nucleic acid architectures. Acc. Chem. Res..

[75] BelBruno J.J. (2019). Molecularly imprinted polymers. Chem. Rev..

[76] Haupt K., Mosbach K. (2000). Molecularly imprinted polymers and their use in biomimetic sensors. Chem. Rev..

[77] Nakatsuka N., Yang K.A., Abendroth J.M., Cheung K.M., Xu X., Yang H., Zhao C., Zhu B., Rim Y.S., Yang Y. (2018). Aptamer-field-effect transistors overcome Debye length limitations for small-molecule sensing. Science.

[78] Rivnay J., Inal S., Salleo A., Owens R.M., Berggren M., Malliaras G.G. (2018). Organic electrochemical transistors. Nat. Rev. Mater..

[79] Khodagholy D., Doublet T., Quilichini P., Gurfinkel M., Leleux P., Ghestem A., Ismailova E., Hervé T., Sanaur S., Bernard C. (2013). In vivo recordings of brain activity using organic transistors. Nat. Commun..

[80] Inal S., Rivnay J., Suiu A.O., Malliaras G.G., McCulloch I. (2018). Conjugated polymers in bioelectronics. Acc. Chem. Res..

[81] Mogera U., Guo H., Namkoong M., Rahman M.S., Nguyen T., Tian L. (2022). Wearable plasmonic paper-based microfluidics for continuous sweat analysis. Sci. Adv..

[82] Jeon J., Lee S., Chae S., Lee J.H., Kim H., Yu E.-S., Na H., Kang T., Park H.-S., Lee D. (2025). All-flexible chronoepifluidic nanoplasmonic patch for label-free metabolite profiling in sweat. Nat. Commun..

[83] Kim J., Jeerapan I., Imani S., Cho T.N., Bandodkar A., Cinti S., Mercier P.P., Wang J. (2016). Noninvasive alcohol monitoring using a wearable tattoo-based iontophoretic-biosensing system. ACS Sens..

[84] Bandodkar A.J., Jia W., Yardımcı C., Wang X., Ramirez J., Wang J. (2015). Tattoo-based noninvasive glucose monitoring: A proof-of-concept study. Anal. Chem..

[85] Wang J. (2008). Electrochemical glucose biosensors. Chem. Rev..

[86] Spinelli J.C., Suleski B.J., Wright D.E., Grow J.L., Fagans G.R., Buckley M.J., Yang D.S., Yang K., Beil S.M., Wallace J.C. (2025). Wearable microfluidic biosensors with haptic feedback for continuous monitoring of hydration biomarkers in workers. npj Digit. Med..

[87] Moonla C., Reynoso M., Casanova A., Chang A.-Y., Djassemi O., Balaje A., Abbas A., Li Z., Mahato K., Wang J. (2024). Continuous ketone monitoring via wearable microneedle patch platform. ACS Sens..

[88] Kjær S.K., Ridder L.O.R., Svart M., Rittig N., Bjerg L.N., Sandfeld-Paulsen B., Thomsen H.H. (2026). Continuous ketone monitoring: Data from a randomized controlled trial. JMIR Diabetes.

[89] Djassemi O., Chang A.-Y., McGuire W.C., Mitchell E., Saha T., Fernandes T., Yang J., Miller M., Wurster C., Morales-Fermin S. (2026). Clinical evaluation of microneedle biosensors for continuous lactate monitoring in critically ill patients. ACS Sens..

[90] Bariya M., Nyein H.Y.Y., Javey A. (2018). Wearable sweat sensors. Nat. Electron..

[91] Yu Y., Nyein H.Y.Y., Gao W., Javey A. (2020). Flexible electrochemical bioelectronics: The rise of in situ bioanalysis. Adv. Mater..

[92] Heller A., Feldman B. (2008). Electrochemical glucose sensors and their applications in diabetes management. Chem. Rev..

[93] Wang M., Yang Y., Min J., Song Y., Tu J., Mukasa D., Ye C., Xu C., Heflin N., McCune J.S. (2022). A wearable electrochemical biosensor for the monitoring of metabolites and nutrients. Nat. Biomed. Eng..

[94] Chen L., Wang X., Lu W., Wu X., Li J. (2016). Molecular imprinting: Perspectives and applications. Chem. Soc. Rev..

[95] Imani S., Bandodkar A.J., Mohan A.M.V., Kumar R., Yu S., Wang J., Mercier P.P. (2016). A wearable chemical-electrophysiological hybrid biosensing system for real-time health and fitness monitoring. Nat. Commun..

[96] Barnett N.P., Celio M.A., Tidey J.W., Murphy J.G., Colby S.M., Swift R.M. (2017). A preliminary randomized controlled trial of contingency management for alcohol use reduction using a transdermal alcohol sensor. Addiction.

[97] Alva S., Castorino K., Cho H., Ou J. (2021). Feasibility of continuous ketone monitoring in subcutaneous tissue using a ketone sensor. J. Diabetes Sci. Technol..

[98] Ye C., Wang M., Min J., Tay R.Y., Lukas H., Sempionatto J.R., Li J., Xu C., Gao W. (2024). A wearable aptamer nanobiosensor for non-invasive female hormone monitoring. Nat. Nanotechnol..

[99] Pei X., Ghandehari A., Chakoma S., Rajendran J., Tavares-Negrete J.A., Esfandyarpour R. (2026). A quantitative, multimodal wearable bioelectronic device for comprehensive stress assessment and sub-classification. Nat. Commun..

[100] Rawson T.M., Gowers S.A.N., Freeman D.M.E., Wilson R.C., Sharma S., Gilchrist M., MacGowan A., Lovering A., Bayliss M., Kyriakides M. (2019). Microneedle biosensors for real-time, minimally invasive drug monitoring of phenoxymethylpenicillin: A first-in-human evaluation in healthy volunteers. Lancet Digit. Health.

[101] Choi Y., Jin P., Lee S., Song Y., Tay R.Y., Kim G., Yoo J., Han H., Yeom J., Cho J.H. (2025). All-printed chip-less wearable neuromorphic system for multimodal physicochemical health monitoring. Nat. Commun..

[102] Fang L., Ren H., Mao X., Zhang S., Cai Y., Xu S., Zhang Y., Li L., Ye X., Liang B. (2022). Differential amperometric microneedle biosensor for wearable levodopa monitoring of Parkinson’s disease. Biosensors.

[103] Wishart D.S. (2019). Metabolomics for investigating physiological and pathophysiological processes. Physiol. Rev..

[104] Patti G.J., Yanes O., Siuzdak G. (2012). Metabolomics: The apogee of the omics trilogy. Nat. Rev. Mol. Cell Biol..

[105] Goud K.Y., Moonla C., Mishra R.K., Yu C., Narayan R., Litvan I., Wang J. (2019). Wearable electrochemical microneedle sensor for continuous monitoring of levodopa: Toward Parkinson management. ACS Sens..

[106] Wu Y., Tehrani F., Teymourian H., Mack J., Shaver A., Reynoso M., Kavner J., Huang N., Furmidge A., Duvvuri A. (2022). Microneedle aptamer-based sensors for continuous, real-time therapeutic drug monitoring. Anal. Chem..

[107] Ito Y., Inagaki Y., Kobuchi S., Takada K., Sakaeda T. (2016). Therapeutic drug monitoring of vancomycin in dermal interstitial fluid using dissolving microneedles. Int. J. Med. Sci..

[108] Rogers J.A., Someya T., Huang Y. (2010). Materials and mechanics for stretchable electronics. Science.

[109] Kim D.-H., Lu N., Ma R., Kim Y.-S., Kim R.-H., Wang S., Wu J., Won S.M., Tao H., Islam A. (2011). Epidermal electronics. Science.

[110] Xu S., Zhang Y., Jia L., Mathewson K.E., Jang K.I., Kim J., Fu H., Huang X., Chava P., Wang R. (2014). Soft microfluidic assemblies of sensors, circuits, and radios for the skin. Science.

[111] Chortos A., Liu J., Bao Z. (2016). Pursuing prosthetic electronic skin. Nat. Mater..

[112] Bandodkar A.J., Jia W., Wang J. (2015). Tattoo-based wearable electrochemical devices: A review. Electroanalysis.

[113] Prausnitz M.R. (2004). Microneedles for transdermal drug delivery. Adv. Drug Deliv. Rev..

[114] Arwani R.T., Tan S.C.L., Sundarapandi A., Goh W.P., Liu Y., Leong F.Y., Yang W., Zheng X.T., Yu Y., Jiang C. (2024). Stretchable ionic–electronic bilayer hydrogel electronics enable in situ detection of solid-state epidermal biomarkers. Nat. Mater..

[115] Dagdeviren C., Joe P., Tuzman O.L., Park K.I., Lee K.J., Shi Y., Huang Y., Rogers J.A. (2016). Recent progress in flexible and stretchable piezoelectric devices for mechanical energy harvesting, sensing and actuation. Extrem. Mech. Lett..

[116] Van Calster B., McLernon D.J., van Smeden M., Wynants L., Steyerberg E.W. (2019). Calibration: The Achilles heel of predictive analytics. BMC Med..

[117] Varma S., Simon R. (2006). Bias in error estimation when using cross-validation for model selection. BMC Bioinform..

[118] Luo W., Phung D., Tran T., Gupta S., Rana S., Karmakar C., Shilton A., Yearwood J., Dimitrova N., Ho T.B. (2016). Guidelines for developing and reporting machine learning predictive models in biomedical research: A multidisciplinary view. J. Med. Internet Res..

[119] Bland J.M., Altman D.G. (1986). Statistical methods for assessing agreement between two methods of clinical measurement. Lancet.

[120] Kottner J., Audigé L., Brorson S., Donner A., Gajewski B.J., Hróbjartsson A., Roberts C., Shoukri M., Streiner D.L. (2011). Guidelines for reporting reliability and agreement studies (GRRAS) were proposed. J. Clin. Epidemiol..

[121] Abadi M., Chu A., Goodfellow I., McMahan H.B., Mironov I., Talwar K., Zhang L. (2016). Deep learning with differential privacy. Proceedings of the 2016 ACM SIGSAC Conference on Computer and Communications Security.

[122] Rieke N., Hancox J., Li W., Milletarì F., Roth H.R., Albarqouni S., Bakas S., Galtier M.N., Landman B.A., Maier-Hein K. (2020). The future of digital health with federated learning. npj Digit. Med..

[123] Kaissis G.A., Makowski M.R., Rückert D., Braren R.F. (2020). Secure, privacy-preserving and federated machine learning in medical imaging. Nat. Mach. Intell..

[124] Roberts M., Driggs D., Thorpe M., Gilbey J., Yeung M., Ursprung S., Aviles-Rivero A.I., Etmann C., McCague C., Beer L. (2021). Common pitfalls and recommendations for using machine learning to detect and prognosticate for COVID-19 using chest radiographs and CT scans. Nat. Mach. Intell..

[125] Moons K.G.M., Damen J.A.A., Kaul T., Hooft L., Navarro C.A., Dhiman P., Beam A.L., Van Calster B., Celi L.A., Denaxas S. (2025). PROBAST+AI: An updated quality, risk of bias, and applicability assessment tool for prediction models using regression or artificial intelligence methods. BMJ.

[126] Kelly C.J., Karthikesalingam A., Suleyman M., Corrado G., King D. (2019). Key challenges for delivering clinical impact with artificial intelligence. BMC Med..

[127] Wiens J., Saria S., Sendak M., Ghassemi M., Liu V.X., Doshi-Velez F., Jung K., Heller K., Kale D., Saeed M. (2019). Do no harm: A roadmap for responsible machine learning for health care. Nat. Med..

[128] Ghassemi M., Naumann T., Schulam P., Beam A.L., Chen I.Y., Ranganath R. (2019). Practical guidance on artificial intelligence for health-care data. Lancet Digit. Health.

[129] Yu K.H., Beam A.L., Kohane I.S. (2018). Artificial intelligence in healthcare. Nat. Biomed. Eng..

[130] Rajkomar A., Dean J., Kohane I. (2019). Machine learning in medicine. N. Engl. J. Med..

[131] Topol E.J. (2019). High-performance medicine: The convergence of human and artificial intelligence. Nat. Med..

[132] U.S. Food and Drug Administration (2025). Marketing Submission Recommendations for a Predetermined Change Control Plan for Artificial Intelligence-Enabled Device Software Functions; Guidance for Industry and Food and Drug Administration Staff. https://www.fda.gov/regulatory-information/search-fda-guidance-documents/marketing-submission-recommendations-predetermined-change-control-plan-artificial-intelligence.

[133] Bossuyt P.M., Reitsma J.B., Bruns D.E., Gatsonis C.A., Glasziou P.P., Irwig L., Lijmer J.G., Moher D., Rennie D., de Vet H.C.W. (2015). STARD 2015: An updated list of essential items for reporting diagnostic accuracy studies. BMJ.

[134] U.S. Food and Drug Administration (2016). Applying Human Factors and Usability Engineering to Medical Devices; Guidance for Industry and Food and Drug Administration Staff. https://www.fda.gov/regulatory-information/search-fda-guidance-documents/applying-human-factors-and-usability-engineering-medical-devices.

[135] U.S. Food and Drug Administration (2025). Pulse Oximeters for Medical Purposes-Non-Clinical and Clinical Performance Testing, Labeling, and Premarket Submission Recommendations: Draft Guidance for Industry and Food and Drug Administration Staff. https://www.fda.gov/regulatory-information/search-fda-guidance-documents/pulse-oximeters-medical-purposes-non-clinical-and-clinical-performance-testing-labeling-and.

[136] Mulholland A.M., MacDonald H.V., Aguiar E.J., Wingo J.E. (2026). Influence of skin pigmentation on the accuracy and data quality of photoplethysmographic heart rate measurement during exercise. Eur. J. Appl. Physiol..

[137] Baker L.B., Model J.B., Barnes K.A., Anderson M.L., Lee S.P., Lee K.A., Brown S.D., Reimel A.J., Roberts T.J., Nuccio R.P. (2020). Skin-interfaced microfluidic system with personalized sweating rate and sweat chloride analytics for sports science applications. Sci. Adv..

[138] Song R., Cho S., Kim G., Shin S., Yin S., Liu R., Zeng R., Tu J., Xiong Y., Park I. (2026). Wireless, battery-free wearable optoelectronic-colorimetric microfluidic sensor for multiplex sweat analysis. Device.

[139] Notley S.R., Park J., Tagami K., Ohnishi N., Taylor N.A.S. (2017). Variations in body morphology explain sex differences in thermoeffector function during compensable heat stress. Exp. Physiol..

[140] Schmidt M.D., Notley S.R., Meade R.D., Akerman A.P., Rutherford M.M., Kenny G.P. (2022). Revisiting regional variation in the age-related reduction in sweat rate during passive heat stress. Physiol. Rep..

[141] Klous L., De Ruiter C., Alkemade P., Daanen H., Gerrett N. (2020). Sweat rate and sweat composition during heat acclimation. J. Therm. Biol..

[142] World Health Organization (2019). Recommendations on Digital Interventions for Health System Strengthening. WHO Guideline.

[143] World Health Organization (2026). Scaling Innovations in Public Health Systems: Guidance and Toolkit.

[144] U.S. Food and Drug Administration (2026). Cybersecurity in Medical Devices: Quality Management System Considerations and Content of Premarket Submissions; Guidance for Industry and Food and Drug Administration Staff. https://www.fda.gov/regulatory-information/search-fda-guidance-documents/cybersecurity-medical-devices-quality-management-system-considerations-and-content-premarket.

[145] Lekadir K., Frangi A.F., Porras A.R., Glocker B., Cintas C., Langlotz C.P., Weicken E., Asselbergs F.W., Prior F., Collins G.S. (2025). FUTURE-AI: International consensus guideline for trustworthy and deployable artificial intelligence in healthcare. BMJ.

[146] U.S. Food and Drug Administration (2026). General Wellness: Policy for Low Risk Devices: Guidance for Industry and Food and Drug Administration Staff. Final Guidance. https://www.fda.gov/regulatory-information/search-fda-guidance-documents/general-wellness-policy-low-risk-devices.

[147] Dervisevic M., Dervisevic E., Esser L., Easton C.D. (2017). Wearable microneedle array-based sensor for transdermal monitoring of pH levels in interstitial fluid. Biosensors and Bioelectronics.

[148] Pal A., Goswami D., Cuellar H.E., Castro B., Kuang S., Martinez R.V. (2018). Early detection and monitoring of chronic wounds using low-cost, omniphobic paper-based smart bandages. Biosens. Bioelectron..

[149] Sharifuzzaman M., Chhetry A., Zahed M.A., Yoon S.H., Park C.I., Zhang S., Barman S.C., Sharma S., Yoon H., Park J.Y. (2020). Smart bandage with integrated multifunctional sensors based on MXene-functionalized porous graphene scaffold for chronic wound care management. Biosens. Bioelectron..

[150] Mostafalu P., Tamayol A., Rahimi R., Ochoa M., Khalilpour A., Kiaee G., Yazdi I.K., Bagherifard S., Dokmeci M.R., Ziaie B. (2018). Smart bandage for monitoring and treatment of chronic wounds. Small.

[151] Tang N., Zheng Y., Cui D., Jiang X., Zhou C., Jin H., Jin K., Wu W., Haick H. (2021). Wearable sensors and systems for wound healing-related pH and temperature detection. Micromachines.

[152] Guinovart T., Valdés-Ramírez G., Windmiller J.R., Andrade F.J., Wang J. (2014). Bandage-based wearable potentiometric sensor for monitoring wound pH. Electroanalysis.

[153] Kassal P., Kim J., Kumar R., de Araujo W.R., Steinberg I.M., Steinberg M.D., Wang J. (2015). Smart bandage with wireless connectivity for uric acid biosensing as an indicator of wound status. Electrochem. Commun..

[154] Mariani F., Serafini M., Gualandi I., Arcangeli D., Decataldo F., Possanzini L., Tessarolo M., Tonelli D., Fraboni B., Scavetta E. (2021). Advanced wound dressing for real-time pH monitoring. ACS Sens..

[155] Arcangeli D., Gualandi I., Mariani F., Tessarolo M., Ceccardi F., Decataldo F., Melandri F., Tonelli D., Fraboni B., Scavetta E. (2023). Smart Bandaid integrated with fully textile OECT for uric acid real-time monitoring in wound exudate. ACS Sens..

[156] Cheng S., Gu Z., Zhou L., Hao M., An H., Song K., Wu X., Zhang K., Zhao Z., Dong Y. (2021). Recent progress in intelligent wearable sensors for health monitoring and wound healing based on biofluids. Front. Bioeng. Biotechnol..

[157] Tottoli E.M., Dorati R., Genta I., Chiesa E., Pisani S., Conti B. (2020). Skin wound healing process and new emerging technologies for skin wound care and regeneration. Pharmaceutics.

[158] McEwen B.S. (1998). Protective and damaging effects of stress mediators. N. Engl. J. Med..

[159] Reinke H., Asher G. (2019). Crosstalk between metabolism and circadian clocks. Nat. Rev. Mol. Cell Biol..

[160] Adam E.K., Kumari M. (2009). Assessing salivary cortisol in large-scale, epidemiological research. Psychoneuroendocrinology.

[161] Hellhammer D.H., Wust S., Kudielka B.M. (2009). Salivary cortisol as a biomarker in stress research. Psychoneuroendocrinology.

[162] Kirschbaum C., Hellhammer D.H. (1994). Salivary cortisol in psychoneuroendocrine research: Recent developments and applications. Psychoneuroendocrinology.

[163] Chrousos G.P. (2009). Stress and disorders of the stress system. Nat. Rev. Endocrinol..

[164] von Elm E., Altman D.G., Egger M., Pocock S.J., Gøtzsche P.C., Vandenbroucke J.P. (2007). The Strengthening the Reporting of Observational Studies in Epidemiology (STROBE) statement: Guidelines for reporting observational studies. PLoS Med..

[165] Schulz K.F., Altman D.G., Moher D., CONSORT Group (2010). CONSORT 2010 statement: Updated guidelines for reporting parallel group randomised trials. BMJ.

[166] Sounderajah V., Guni A., Liu X., Collins G.S., Karthikesalingam A., Markar S.R., Golub R.M., Denniston A.K., Shetty S., Moher D. (2025). The STARD-AI reporting guideline for diagnostic accuracy studies using artificial intelligence. Nat. Med..

[167] Whiting P.F., Rutjes A.W.S., Westwood M.E., Mallett S., Deeks J.J., Reitsma J.B., Leeflang M.M.G., Sterne J.A.C., Bossuyt P.M.M., QUADAS-2 Group (2011). QUADAS-2: A revised tool for the quality assessment of diagnostic accuracy studies. Ann. Intern. Med..

[168] Moher D., Liberati A., Tetzlaff J., Altman D.G., PRISMA Group (2009). Preferred reporting items for systematic reviews and meta-analyses: The PRISMA statement. PLoS Med..

[169] Vayena E., Blasimme A., Cohen I.G. (2018). Machine learning in medicine: Addressing ethical challenges. PLoS Med..

